# Modular Chemical Construction of IgG-like Mono- and Bispecific Synthetic Antibodies (SynAbs)

**DOI:** 10.1021/acscentsci.2c01437

**Published:** 2023-02-21

**Authors:** Fabien Thoreau, Peter A. Szijj, Michelle K. Greene, Léa N. C. Rochet, Ioanna A. Thanasi, Jaine K. Blayney, Antoine Maruani, James R. Baker, Christopher J. Scott, Vijay Chudasama

**Affiliations:** † Department of Chemistry, University College London, 20 Gordon Street, London WC1H 0AJ, U.K.; ‡ Patrick G Johnston Centre for Cancer Research, School of Medicine, Dentistry and Biomedical Sciences, Queen’s University Belfast, Belfast BT9 7AEU.K.

## Abstract

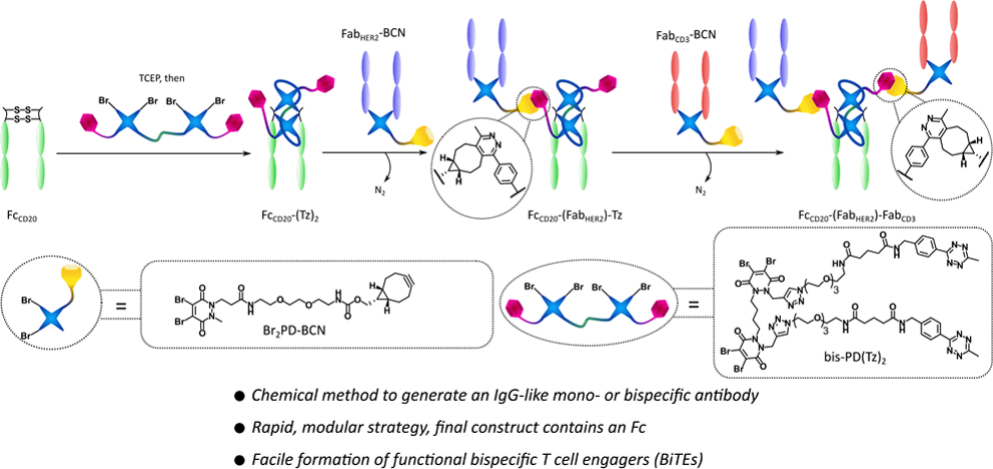

In recent years there has been rising interest in the field of protein–protein conjugation, especially related to bispecific antibodies (bsAbs) and their therapeutic applications. These constructs contain two paratopes capable of binding two distinct epitopes on target molecules and are thus able to perform complex biological functions (mechanisms of action) not available to monospecific mAbs. Traditionally these bsAbs have been constructed through protein engineering, but recently chemical methods for their construction have started to (re)emerge. While these have been shown to offer increased modularity, speed, and for some methods even the inherent capacity for further functionalization (e.g., with small molecule cargo), most of these approaches lacked the ability to include a fragment crystallizable (Fc) modality. The Fc component of IgG antibodies offers effector function and increased half-life. Here we report a first-in-class disulfide rebridging and click-chemistry-based method for the generation of Fc-containing, IgG-like mono- and bispecific antibodies. These are in the Fc_Z_-(Fab_X_)-Fab_Y_ format, i.e., two distinct Fabs and an Fc, potentially all from different antibodies, attached in a homogeneous and covalent manner. We have dubbed these molecules synthetic antibodies (SynAbs). We have constructed a T cell-engager (TCE) SynAb, Fc_CD20_-(Fab_HER2_)-Fab_CD3_, and have confirmed that it exhibits the expected biological functions, including the ability to kill HER2^+^ target cells in a coculture assay with T cells.

## Abstract 2

In this article, we report a first-in-class method to produce a “synthetic antibody” (SynAb) from the fragments of three different parent antibodies using chemical methods.

## Introduction

Antibodies are symmetrical proteins composed of two identical fragment antigen-binding (Fab) domains responsible for their binding to a specific target, and one fragment crystallizable (Fc) conferring them immune-effector capacity and increased half-life. Bispecific antibodies (bsAbs) are (usually) artificial proteins containing two different binding elements (not necessarily Fab moieties) enabling their interaction with two epitopes of the same target or, in most cases, interaction with epitopes of two different targets.^1^ The capacity of bsAbs to simultaneously interact with two targets/receptors offers various applications: 1) redirection of immune cells such as T cells, NK cells, or macrophages toward tumor cells in order to trigger or improve immunosuppression of the tumor (such bsAbs are referred to as “immune cell engagers” and have had a huge impact on the immunotherapy landscape);^2^ 2) the simultaneous modulation of two different pathways in pathogenesis;^3^ 3) increasing selectivity and/or avidity for a target cell by interacting with two different antigens at the cell surface (or two epitopes of the same antigen);^4^ 4) holding effector proteins together as a substitute for an inactivated or faulty scaffold protein.^5,6^ The majority of reported bsAbs in the literature and clinical trials are T cell engagers (TCEs).^2^ These TCEs are designed to recruit immune cells to the tumor site by combining affinity for a receptor on the surface of T cells (usually CD3, a T cell coreceptor involved in T cell activation) and a tumor associated antigen (TAA). While one side of the bsAb interacts with a T cell, activating it, the other side can interact with a target cell, bringing them into close vicinity and leading to formation of an immunological synapse which allows target cell destruction by the activated T cell.

Following initial clinical success in the early 2000s, bsAbs have been extensively studied, and multiple bispecific antibody formats have been designed—more than a hundred—where the binding elements can either be complete Fab moieties, or only portions of a Fab moiety such as scFv, DART, or diabody. This multitude of bispecific antibody formats has been extensively reviewed elsewhere.^7−9^ Interestingly, some bsAb formats possess an Fc moiety (“IgG-like bispecific antibodies”), while others do not (“bispecific antibody fragments”).

Among the wide variety of bispecific antibody formats developed, fuelled by the search for optimal efficacy, geometry, half-life, stability, solubility, scalability or reproducibility, or the production of new intellectual property (IP), the method employed to produce them is almost exclusively protein bioengineering, generating so-called recombinant or fusion proteins.^8,9^ Indeed, as early as in the 1960s, the first attempts at the chemical production of bispecific formats suffered from poor yields and complicated purification protocols.^10,11^ Quickly, the production of bsAbs evolved to make use of quadroma cell line technology. Later on, several technologies improved the bioengineered production of bsAbs, including the “knobs-into-holes” (KIH),^12^ the CrossMab,^13^ and the FORCE (“Format Chain Exchange”)^14^ approaches. Indeed, asymmetric, IgG-like formats made up the majority (60%) of the clinical pipeline as of 2019.^15^ Despite obvious benefits, notably in terms of efficiency and high scalability, the use of bioengineering for bsAb production suffers from a lack of modularity—each new bsAb requires creation of new recombinant DNA sequences and expression of the related recombinant protein. Furthermore, the expression titers of bsAbs are often lower than for monospecific antibodies, and isolation of the bsAb requires extensive purification, further lowering yields.^16^ The process can thus be time- and cost-intensive.

With recent improvements in the field, chemical and bioconjugation-based methods for the production of bsAbs have started appearing as valuable complementary approaches and potential alternatives to genetic engineering.^17^ Indeed, organic chemistry/bioconjugation allows for the introduction of complementary chemical handles on two or more distinct proteins (native or recombinant) to enable their covalent assembly and thus generate a new protein construct, such as a bsAb. This strategy has been empowered by recent progress in selective protein modification,^18,19^ and the development of ultrafast, metal-free, and bioorthogonal click reactions,^20^ including strain-promoted azide–alkyne cycloaddition (SPAAC),^21^ strain-promoted alkyne-nitrone cycloaddition (SPANC),^22^ or inverse electron-demand Diels–Alder (iEDDA) reactions involving partners such as tetrazine with trans-cyclooctene or strained alkynes, or strained alkyne with fluorosydnone.^23−25^ The possibility to modify individual proteins and quickly assemble them into a bsAb could offer benefits over bioengineering regarding the modularity and speed of production. Furthermore, the chemical tools employed for protein modification and assembly also offer the opportunity to introduce additional functionality on the protein construct (e.g., toxins, fluorophores, sensitizers for bsAb-conjugates, or masking moieties) similarly to how ADCs or probodies are produced.^18,26−28^ This approach could also allow for varying of the nature, length, flexibility, and stability of the linkers between the protein fragments. If the “chemical approach” was to become more mainstream, it could also facilitate access to these constructs for chemistry research teams without ready access to bioengineering. Nonetheless, the bioengineering and chemical methods to generate bsAbs and other protein constructs are not necessarily meant to compete. While the high scalability of bioengineering is well optimized, this aspect of chemical methods still needs to be investigated. However, chemical approaches applied at milligram to gram scales have demonstrated high speed and modularity, which are beneficial traits for high throughput screening processes not afforded by protein engineering. This makes chemical strategies a valuable complementary tool to recombinant technologies, e.g., by rapid chemical hit candidate-identification before an efficient fusion-based scale up.

The most modular methods in the field of bioconjugation strive to selectively introduce a chemical handle into the protein of interest—allowing subsequent modification. This handle can then be functionalized with an effector of choice (drug, fluorophore, or other protein) through a bioorthogonal click reaction. Due to the site-selective nature of the initial protein modification and the specificity of the click reaction employed, homogeneous protein–conjugates can be generated. Several tools have been developed for the selective chemical modification of proteins, exploiting solvent accessible (usually nucleophilic) amino acid side-chains (e.g., lysine, tyrosine, or cysteine) for functionalization.^19,26,29,30^ Other methods based on *N*-terminal modification, enzymatic or chemoenzymatic processes, or the introduction of noncanonical amino acids with modification tags incorporated have also been developed.^19^ A subset of cysteine-reactive modalities, disulfide rebridging reagents, rely on the reduction of accessible disulfide bridges followed by their covalent reconnection via a small molecule—indeed these strategies have been used for the generation of protein–protein conjugates.^31,32^ For this purpose, the Chudasama and Baker groups developed the pyridazinedione (PD) scaffold,^33−36^ a chemical platform bearing: 1) two leaving groups across the double bond (generally bromides) capable of reacting with the two liberated sulfhydryl groups generated via disulfide reduction, allowing for the covalent rebridging of the disulfide; 2) up to two chemical handles for orthogonal click reactions, allowing selective dual-modification of the protein. In a three-step protocol (reduction, rebridging, and click reaction), the PD platform allows for the generation of protein-conjugates with controlled and homogeneous conjugate/protein ratios. As an example, Maruani et al. could generate a dually functionalized antibody,^37^ and a dually functionalized Fab_X_-Fab_Y_ bispecific antibody construct (note the lack of an Fc fragment in this case).^27^ PD platforms, combined with efficient click reactions, have thus proved to be valuable tools for protein modification and protein construct assembly.

In this paper we attempted a first-in-class purely chemical construction of full antibody-like constructs, dubbed SynAbs (synthetic antibodies) from the Fab and Fc fragments of commercially available antibodies. We described the generation of both a monospecific and bispecific SynAb in this manner. All the fragments were obtained by enzymatic digestion from commercially available native mAbs, and the process relied completely on PD-mediated bioconjugation and Cu-free click chemistry with no protein engineering required. The SynAb constructs were evaluated for their biological activity. The Fc-containing bispecific SynAb was the first strategy described for chemically generating a bsAb with potential access to the biological functionality that could be provided by an Fc such as half-life extension or effector function.^38,39^ Thus, this work represents a major contribution to the field of chemical bsAb-production.

## Results

Prompted by our recent advances developing a pyridazinedione-based chemical method to produce a Fab-Fab bsAb format,^27^ we decided to evolve the method further and adapt it to the production of Fc-containing IgG-like Abs and bsAbs, dubbed SynAbs (synthetic antibodies). The combination of two Fab targeting modules and an Fc moiety in the same antibody construct is appealing in many situations due to increased half-life and/or Fc-mediated effector function it can provide.^38,39^ This approach is far more challenging than the previously reported Fab-Fab format as it involves the separate selective chemical modification, followed by assembly, of three individual protein modules. We theorized that this would be best achieved with pyridazinedione-based methods as they offer high site-selectivity in addition to excellent modularity due to the two possible functional handles they can bear. Additionally, we have shown previously that this rebridging strategy does not affect the biological activity of the Fc or the Fab.^40^ Our strategy to make IgG-like mono- and bispecific SynAbs was to 1) enzymatically generate and isolate various protein fragments (Fab and Fc) from their parent monoclonal antibodies, and 2) individually modify the isolated fragments with pyridazinedione (PD) molecular platforms bearing different click handles via disulfide rebridging. Importantly, the handle incorporated in the Fab moieties had to be reactive toward the ones introduced on the Fc. 3) These preparations would then culminate in the covalent assembly of the three individual protein fragments into a mono- or bispecific SynAb through selective click ligation.

As demonstrated before in our group,^34,36^ a crucial advantage of the Br_2_PD-based method is the selective rebridging of solvent-accessible disulfides in proteins. This leads to the controlled introduction of only one PD per Fab fragment, which presents only one accessible interchain disulfide; and two PDs on the Fc moiety, which has two accessible disulfides located in its hinge region (in case of an IgG1 parent isoform such as rituximab). Thus, if the introduced Br_2_PD motifs each contain one click handle, they confer only one modification site for the Fab, and two for the Fc, upon click reaction. With this methodology, controlled modification of Fab or Fc fragments with various small molecules (drug, fluorophore, etc.) is possible.^37,41^ However, here, we intended to connect two “mono-clickable Fab” moieties to the “dually-clickable Fc” moiety. If two identical Fabs (two Fab_X_) are chemically connected to Fc_Z_, a monospecific Fc_Z_-(Fab_X_)_2_ SynAb would be generated, while two different Fabs (Fab_X_ and Fab_Y_) chemically connected to the Fc would yield a bispecific Fc_Z_-(Fab_X_)-Fab_Y_ SynAb.

### Generation of HER2/HER2 SynAb **1** and **2**

#### Mono-PD Method

As a proof-of-concept, we first attempted to produce a monospecific SynAb (Fc_Z_-(Fab_X_)_2_). The process consisted of enzymatic digestion of commercially available native anti-HER2 (trastuzumab) and anti-CD20 (rituximab) antibodies to isolate the Fab_HER2_ and Fc_CD20_ fragments, respectively, following previously described procedures (see Supporting Information (SI) for details).^27^ We chose fragments from two different, clinically approved, antibodies to show that the method is not limited to fragments from a single parent mAb. After site-selective modification of the Fab and Fc fragments with Br_2_PD molecules bearing complementary click handles, a click reaction between the mono clickable-Fab and dually clickable-Fc would enable the construction of an IgG-like SynAb construct, Fc_CD20_-(Fab_HER2_)_2_**1**. The click reactions used to connect proteins have to be both bioorthogonal to avoid undesired cross-linking and ultrafast to counteract the steric hindrance of the protein partners that makes protein–protein cross-linking slow. For this purpose, we chose to work with the tetrazine–BCN (bicyclononyne) click ligation to chemically produce the SynAb, as it is fast (up to 10^4^ M^–1^·s^–1^ in MeOH/H_2_O solution),^42^ compatible with aqueous media, and the resulting pyridazine linkage is stable.^17,43^ This strategy required the synthesis of the corresponding Br_2_PD-tetrazine **3** and Br_2_PD-BCN **4** to be incorporated in the Fc and Fab fragments. These molecules were synthesized as before in our group (see SI for details).^27^

Next, we proceeded with the selective modification of Fab_HER2_ and Fc_CD20_. For both, a two-step procedure was employed, consisting of 1) reduction of accessible disulfide bridge(s) with excess TCEP over 1–2 h, followed by removal of the remaining TCEP through ultrafiltration/buffer-exchange; and 2) disulfide rebridging with excess of Br_2_PD-Tz **3** or Br_2_PD-BCN **4** (10 to 20 equiv for 2–4 h) before removal of unreacted Br_2_PD via buffer exchange/ultrafiltration.

**Figure 1:**
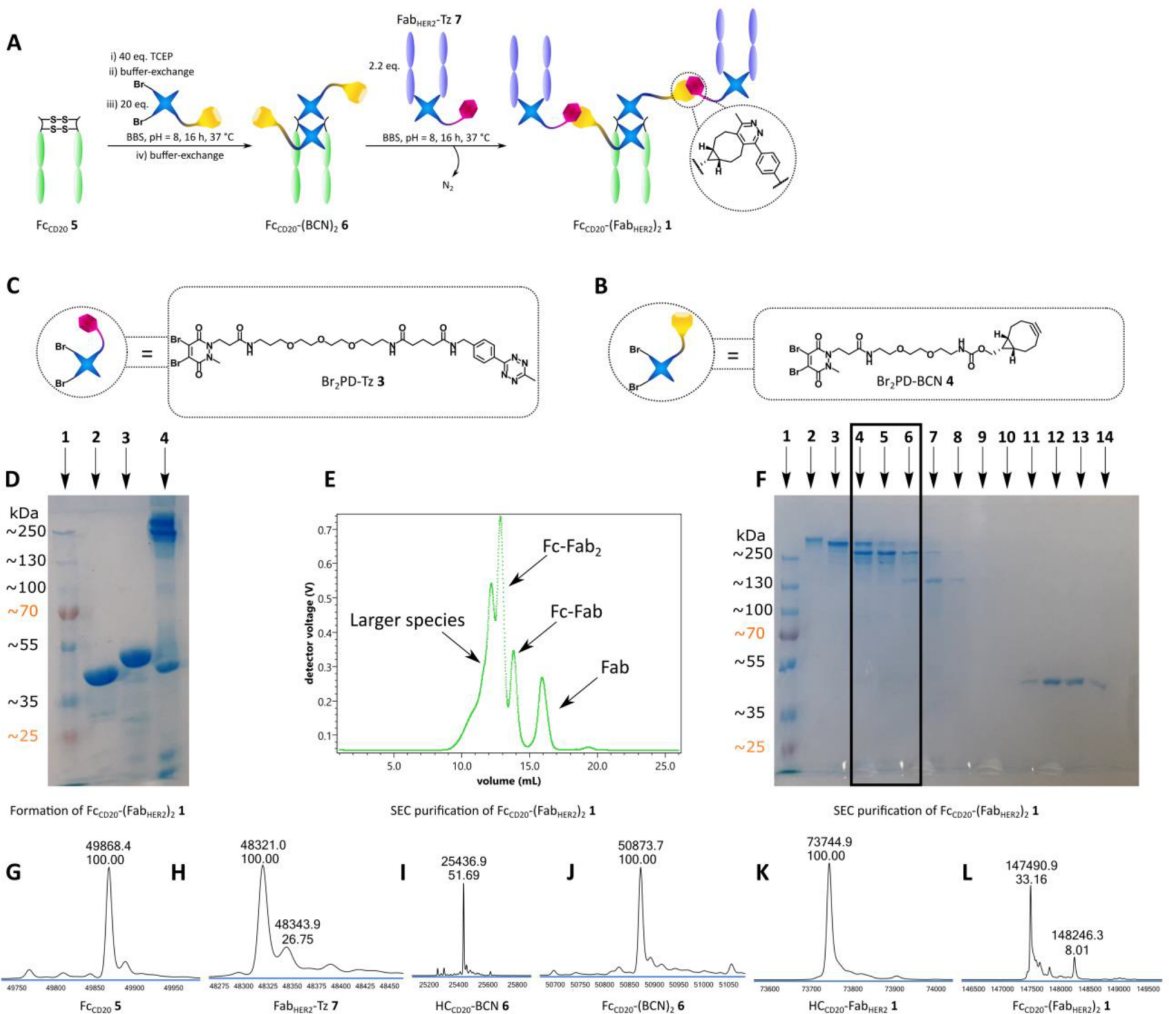
Chemical construction of a full antibody to generate Fc_**CD20**_-(Fab_HER2_)_**2**_SynAb 1. **A** Mono-PD method for the construction of a full antibody Fc_CD20_-(Fab_HER2_)_2_ SynAb **1**. Fc_CD20_**5** is sequentially reduced and rebridged with Br_2_PD-BCN **4**. The resulting Fc_CD20_-(BCN)_2_**6** is then reacted with Fab_HER2_-Tz **7** to generate Fc_CD20_-(Fab_HER2_)_2_ SynAb **1** after SEC purification. **B** Br_2_PD-Tz **3** used for SynAb synthesis. **C** Br_2_PD-BCN **4** used for SynAb synthesis. **D** SDS-PAGE analysis of Fc_CD20_-(Fab_HER2_)_2_ SynAb **1** formation. Lane 1: Ladder. Lane 2: Fab_HER2_**8**. Lane 3: Fc_CD20_**5**. Lane 4: Crude Fc_CD20_-(Fab_HER2_)_2_ SynAb **1**. **E** UV trace of SEC purification of Fc_CD20_-(Fab_HER2_)_2_ SynAb **1**. **F** SDS-PAGE analysis of SEC purification of SynAb **1**. Lane 1: Ladder. Lane 2–3: Aggregates. Lane 4–6: SynAb **1**. Lane 7–8: BCN-Fc_CD20_-Fab_HER2_. Lane 11–14: Fab_HER2_**8**. **G** LC-MS analysis of Fc_CD20_**5**. Observed mass: 49868 Da. **H** LC-MS analysis of Fab_HER2_-Tz **7**. Expected mass: 48334 Da. Observed mass: 48321 Da. **I**, **J** LC-MS analysis of Fc_CD20_-(BCN)_2_**6**. Expected mass: 50873 Da (Fc_CD20_-(BCN)_2_**6**) and 25438 Da (disulfide scrambled Fc_CD20_-(BCN)_2_**6**). Observed mass: 50874 and 25437 Da. **K**, **L** LC-MS analysis of Fc_CD20_-(Fab_HER2_)_2_ SynAb **1**. Expected mass: 147486 Da (Fc_CD20_-(Fab_HER2_)_2_**1**) and 73744 Da (disulfide scrambled Fc_CD20_-(Fab_HER2_)_2_**1**). Observed mass: 147491 Da, 148246 Da (Δ = 755 Da) and 73745 Da.

Successful incorporation of one Br_2_PD-Tz **3** molecule into Fab_HER2_**8** or two Br_2_PD-BCN **4** into the Fc_CD20_**5** to yield Fab_HER2_-Tz **7** and Fc_CD20_-(BCN)_2_**6**, respectively, could be confirmed by LC-MS analysis (Figure 1/H-J). However, as expected, we also observed that rebridging of the Fc with two Br_2_PD-BCN **4** molecules led to some, albeit minimal, disulfide scrambling when each Br_2_-PD-BCN **4** connects the two −SH of the same heavy chain (intrachain rebridging) rather than those of the two heavy chains (interchain rebridging). This phenomenon is well-known in the case of mAb rebridging, and in that case leads to so-called “half antibody” formation. Noncovalent interactions will hold the two heavy chains together in solution, and it has been shown that disulfide scrambling has no impact on antigen binding and minimal impact on Fc-mediated function in the case of an IgG1.^40^ However, under denaturing analytical conditions (SDS-PAGE or LC-MS), the disulfide scrambled species, HC_CD20_-BCN in the case of Fc_CD20_-(BCN)_2_**6**, can be observed (Figure 1/I). It is important to note that while these scrambled species appeared in the LC-MS spectra, this was a major overrepresentation of their actual abundance—SDS-PAGE (Figure 1/D,F) clearly shows that this was a minor species in the case of both Fc_CD20_-(BCN)_2_**6** and Fc_CD20_-(Fab_HER2_)_2_ SynAb **1**. But even if this species was more abundant, as detailed before, it would not be expected to affect the biological function of the construct.^40^

The next step consisted of reacting 2.2 equiv of Fab_HER2_-Tz **7** with the Fc_CD20_-(BCN)_2_**6** to generate Fc_CD20_-(Fab_HER2_)_2_ SynAb **1**. SDS-PAGE analysis showed that after 16 h, all Fc had been consumed in the crude reaction (Figure 1/D). Satisfyingly, Fc_CD20_-(Fab_HER2_)_2_ SynAb **1** was isolated after SEC purification (Figure 1/E) as confirmed by SDS-PAGE (Figure 1/F) and LC-MS analysis (Figure 1/K,L) in 15% yield (calculated from Fc_CD20_**5**). We highlight that, to the best of our knowledge, this is the very first time that an IgG-like antibody construct has been generated exclusively via chemical methods.

#### Bis-PD Method

**Figure 2:**
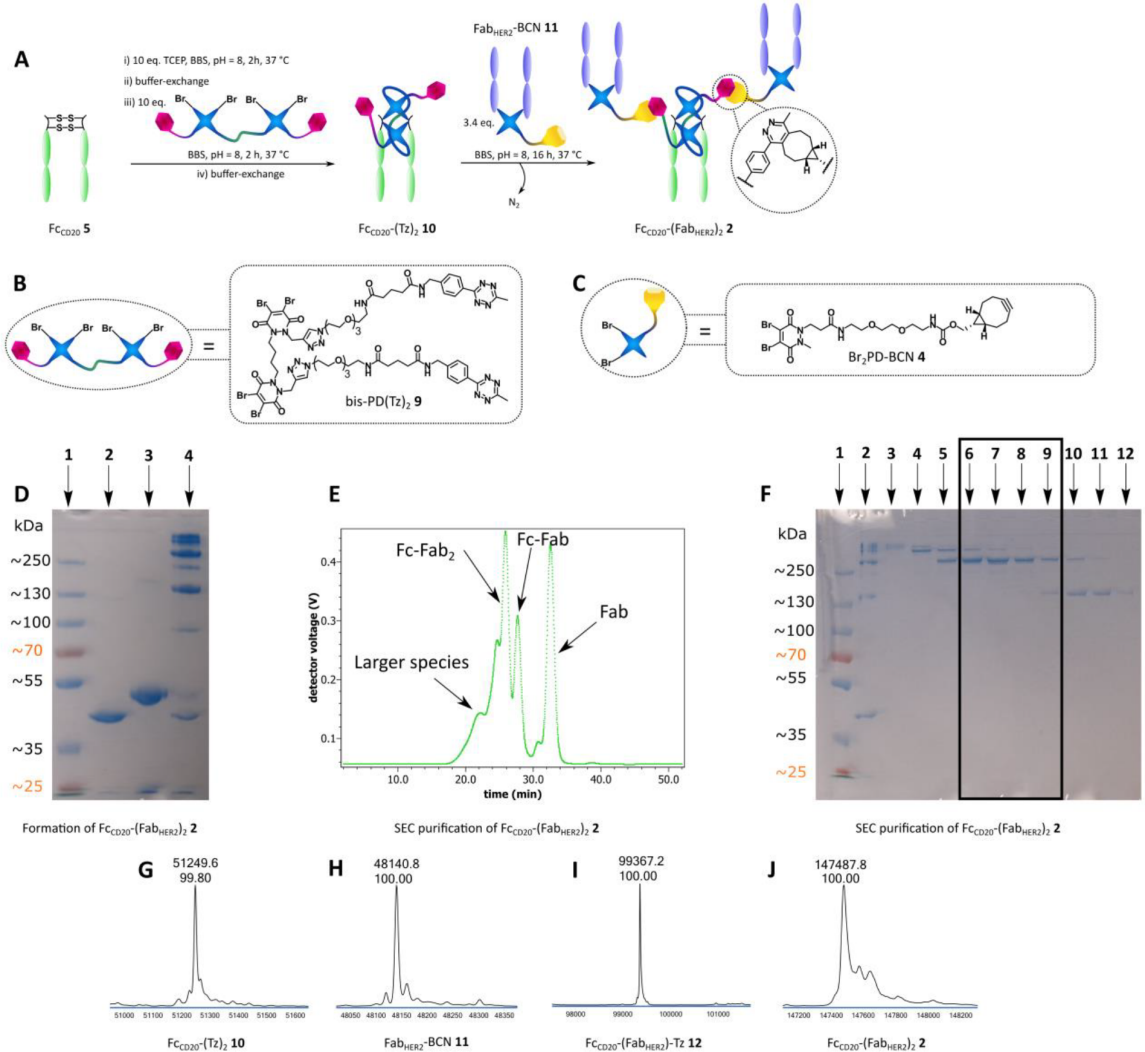
Chemical construction of a full antibody to generate Fc_CD20_-(Fab_HER2_)_**2**_SynAb 2. **A** Bis-PD method for the construction of a full antibody Fc_CD20_-(Fab_HER2_)_2_ SynAb **2**. Fc_CD20_**5** is sequentially reduced and rebridged with bis-PD(Tz)_2_**9**. The resulting Fc_CD20_-(Tz)_2_**10** is then reacted with Fab_HER2_-BCN **11** to generate Fc_CD20_-(Fab_HER2_)_2_ SynAb **2** after SEC purification. **B** Bis-PD(Tz)_2_**9** used for SynAb synthesis. **C** Br_2_PD-BCN **4** used for SynAb synthesis. **D** SDS-PAGE analysis of Fc_CD20_-(Fab_HER2_)_2_ SynAb **2** formation. Lane 1: Ladder. Lane 2: Fab_HER2_-BCN **11**. Lane 3: Fc_CD20_-(Tz)_2_**10**. Lane 4: crude Fc_CD20_-(Fab_HER2_)_2_ SynAb **2**. **E** UV trace of SEC purification of Fc_CD20_-(Fab_HER2_)_2_ SynAb **2**. **F** SDS-PAGE analysis of SEC purification of Fc_CD20_-(Fab_HER2_)_2_ SynAb **2**. Lane 1: Ladder. Lane 2–5: Large species. Lane 6–9: Fc_CD20_-(Fab_HER2_)_2_ SynAb **2**. Lane 10–12: Fc_CD20_-(Fab_HER2_)-Tz **12**. **G** LC-MS analysis of Fc_CD20_-(Tz)_2_**10**. Expected mass: 51246 Da. Observed mass: 51250 Da. **H** LC-MS analysis of Fab_HER2_-BCN **11**. Expected mass: 48141 Da. Observed mass: 48141 Da. **I** LC-MS analysis of Fc_CD20_-(Fab_HER2_)-Tz **12**. Expected mass: 99359 Da. Observed mass: 99367 Da. **J** LC-MS analysis of Fc_CD20_-(Fab_HER2_)_2_ SynAb **2**. Expected mass: 147472 Da. Observed mass: 147488 Da.

With these initial promising results in hand regarding the production of SynAb **1**, we set about developing a more elegant method that eliminated the (admittedly minor) issues with disulfide scrambling. It was proposed that using a bis-PD linker (where two Br_2_PD functionalities are covalently linked) to install click handles onto the Fc could be the solution. While disulfide scrambling would still occur, the covalent linkage between the two heavy chains of the Fc would be maintained regardless. An additional benefit to this approach is making LC-MS analysis simpler as there would be only one expected product mass. Hence, a new synthetic route was developed to generate an appropriate click-enabled bis-PD. The click handle chosen for the bis-PD was tetrazine (Figure 2/B, see SI for details on synthesis), as in our experience it is more stable, which would be needed over a longer 2-step reaction protocol, and thus easier to work with than BCN, especially for the synthesis, handling, and storage of the small molecule. We were a bit tentative on this approach being successful as the use of Br_2_PD-Tz did not lead to full modification of Fc_CD20_ (see SI for LC-MS spectrum), but we felt that the intramolecular nature of the second addition of PD would obviate this issue.

New trials of SynAb production were carried out with similar conditions to those described above, except that 7.5 equiv of bis-PD(Tz)_2_**9** was employed to rebridge Fc_CD20_**5**, and Fab_HER2_**8** was correspondingly functionalized with Br_2_PD-BCN **4** (Figure 2/A). To our delight, good purity for both Fc_CD20_-(Tz)_2_**10** and Fab_HER2_-BCN **11** was obtained as confirmed by LC-MS analysis (Figure 2/G,H). In addition, SDS-PAGE gel analysis revealed only one band corresponding to Fc_CD20_-(Tz)_2_**10**, confirming that the bis-PD compound prevents formation of half-Fc by cross-linking the two heavy chains regardless of the way the disulfides are rebridged by the compound (Figure 2/D). The freshly prepared Fc_CD20_-(Tz)_2_**10** (1 equiv) and Fab_HER2_-BCN **11** (3.4 equiv) were then mixed together overnight at 37 °C in BBS buffer pH 8 to generate the Fc_CD20_-(Fab_HER2_)_2_ SynAb construct **2** via a SPIEDAC reaction. According to SDS-PAGE (Figure 2/D) and SEC UV (Figure 2/E) analysis, complete consumption of Fc_CD20_-(Tz)_2_**10** was achieved after this time. However, more Fc_CD20_-(Fab_HER2_)-Tz **12** monoadduct was observed than with the previous mono-PD strategy, suggesting worse conversion from the monoadduct to the diadduct than previously, even with higher equivalents of Fab_HER2_ used in this case. In any event, LC-MS analysis confirmed the formation and isolation of Fc_CD20_-(Fab_HER2_)_2_ SynAb **2** with satisfactory purity (Figure 2/J) in 11% yield (calculated from Fc_CD20_**5**). Crucially, this time no “half antibody” type species was observed, with the product represented by a single peak in LC-MS analysis, validating the use of a bis-PD strategy for SynAb assembly.

### Generation of the HER2/CD3 Bispecific Fc_CD20_-(Fab_HER2_)-Fab_CD3_ SynAb **13**

The production of the Fc_CD20_-(Fab_HER2_)_2_ SynAbs **1** and **2** was successful. However, the utility of this IgG-like antibody construct is moderate and was merely envisaged as a proof-of-concept. Granted, the ability to vary the Fc compared to the Fab modules can offer some benefits, as the Fc can be extensively engineered to extend or reduce half-life and/or effector function, or to otherwise modulate the bioavailability of the antibody—we can envision a process where a targeting modality could be coupled to an Fc of choice (chosen from a library of various engineered Fc modalities for instance) based on these parameters.^44−46^ But to unlock the full potential of the method, we moved on to our main goal—the chemical production of an IgG-like bispecific SynAb. Since T cell engagers (TCEs) are perhaps the most therapeutically relevant class of bsAbs, we chose to attempt the grafting of a Fab_HER2_ and a Fab_CD3_ on Fc_CD20_, with the aim to produce an “IgG-like TCE”, able to recruit T cells (through CD3-binding) to HER2^+^ tumor cells. While incorporating an Fc moiety into these construct can be crucial for half-life extension,^47−49^ the presence of an Fc with immune-effector function is not beneficial in the context of a TCE strategy, and is in fact detrimental due to the undesired depletion of engaged T cells and due to cytokine release syndrome (CRS) arising from immune overactivation through FcγR engagement.^50^ As in this case the goal was to generate a proof-of-concept bispecific SynAb rather than a therapeutically relevant moiety, we used Fc_CD20_**5** even though as a native Fc it has effector function which is suboptimal for a TCE. For other applications where the dual targeting of a bsAb is exploited, the effector function of an Fc (ADCC, ADCP, etc.)^44,51^ can be beneficial in addition to half-life extension. In either case, the Fc can also be a platform for further modification (e.g., sugar/glutamine modification).^52,53^

**Figure 3:**
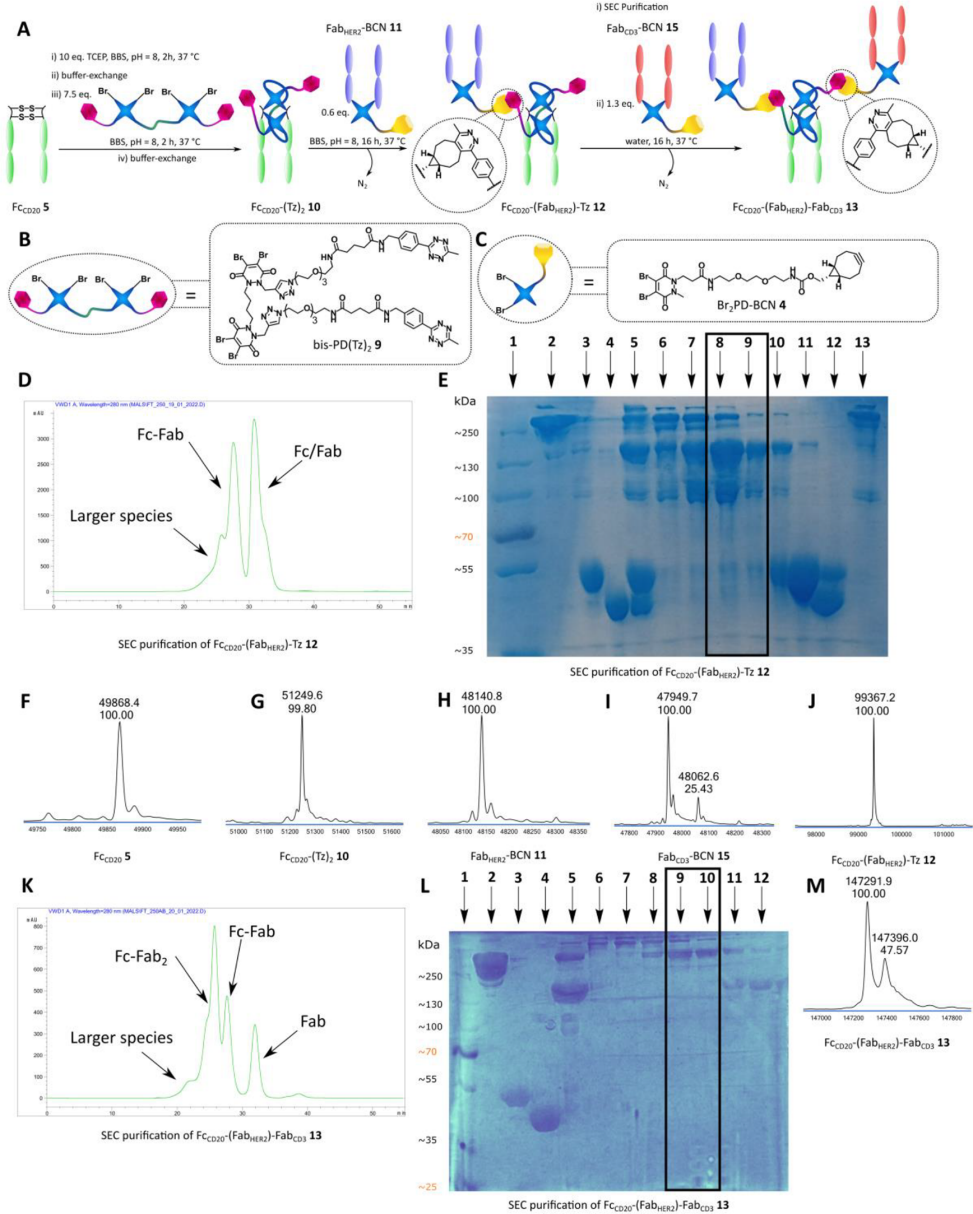
Chemical generation of bispecific Fc_CD20_-(Fab_HER2_)-Fab_CD3_SynAb **13**. **A** Bis-PD method for the construction of bispecific Fc_CD20_-(Fab_HER2_)-Fab_CD3_ SynAb **13**. Fc_CD20_**5** is sequentially reduced and rebridged with bis-PD(Tz)_2_**9**. The resulting Fc_CD20_-(Tz)_2_**10** is then reacted with Fab_HER2_-BCN **11** to generate Fc_CD20_-(Fab_HER2_)-Tz **12** after SEC purification. Fc_CD20_-(Fab_HER2_)-Tz **12** is reacted further with Fab_CD3_-BCN **15** to generate bispecific Fc_CD20_-(Fab_HER2_)-Fab_CD3_ SynAb **13** after SEC purification. **B** The bis-PD(Tz)_2_**9** used for bispecific SynAb synthesis. **C** Br_2_PD-BCN **4** used for bispecific SynAb synthesis. **D** UV trace of SEC purification of Fc_CD20_-(Fab_HER2_)-Tz **12**. **E** SDS-PAGE analysis of Fc_CD20_-(Fab_HER2_)-Tz **12** intermediate formation and SEC purification. Lane 1: Ladder. Lane 2: Anti-HER2 mAb (trastuzumab). Lane 3: Fc_CD20_-(Tz)_2_**10**. Lane 4: Fab_HER2_-BCN **11**. Lane 5: Crude Fc_CD20_-(Fab_HER2_)-Tz **12**. Lane 6–7 and 13: Fc_CD20_-(Fab_HER2_)_2_**2** byproduct/larger species. Lane 8–9: Purified Fc_CD20_-(Fab_HER2_)-Tz **12**. Lane 10–12: Left-over Fab and Fc species. **F** LC-MS analysis of Fc_CD20_**5**. Observed mass: 49868 Da. **G** LC-MS analysis of Fc_CD20_-(Tz)_2_**10**. Expected mass: 51246 Da. Observed mass: 51250 Da. **H** LC-MS analysis of Fab_HER2_-BCN **11**. Expected mass: 48141 Da. Observed mass: 48141 Da. **I** LC-MS analysis of Fab_CD3_-BCN **15**. Expected mass: 47948 Da. Observed mass: 47950 Da. **J** LC-MS analysis of Fc_CD20_-(Fab_HER2_)-Tz **12**. Expected mass: 99359 Da. Observed mass: 99367 Da. **K** UV trace of SEC purification of bispecific Fc_CD20_-(Fab_HER2_)-Fab_CD3_ SynAb **13**. **L** SDS-PAGE analysis of SEC purification of Fc_CD20_-(Fab_HER2_)-Fab_CD3_ SynAb **13**. Lane 1: Ladder. Lane 2: Anti-HER2 mAb (trastuzumab). Lane 3: Fc_CD20_-(Tz)_2_**10**. Lane 4: Fab_HER2_-BCN **11**. Lane 5: Fc_CD20_-(Fab_HER2_)-Tz **12** intermediate. Lane 6–8: Large species. Lane 9–10: Fc_CD20_-(Fab_HER2_)-Fab_CD3_ SynAb **13**. Lane 11–12: Left-over Fc_CD20_-(Fab_HER2_)-Tz **12**. **M** LC-MS analysis of bispecific Fc_CD20_-(Fab_HER2_)-Fab_CD3_ SynAb **13**. Expected mass: 147284 and 147398 Da. Observed mass: 147292 and 147396 Da.

We thus attempted the production of an IgG-like TCE to exemplify the potential of our method. The strategy was based on the bis-PD method described above for monospecific SynAb production, albeit with some slight modifications. A symmetric bis-PD, with two equivalent tetrazine handles, was used to functionalize Fc_CD20_**5**. Thus, Fab_HER2_**8** and Fab_CD3_**14** had to be introduced sequentially for maximum homogeneity. This strategy necessitated an intermediate SEC purification step after the addition of Fab_HER2_-BCN **11** to Fc_CD20_-(Tz)_2_**10**, to ensure isolation of Fc_CD20_-(Fab_HER2_)-Tz **12** (Figure 3/A).

Digestion of anti-CD3 antibody was carried out successfully to generate Fab_CD3_**14** (see SI for details). With the fragments in hand, optimization of the previous SynAb procedure allowed reduction (10 equiv of TCEP) and rebridging of Fc_CD20_**5** with 7.5 equiv of bis-PD(Tz)_2_**9** in a two-step procedure (4 h overall). Both Fab_HER2_**8** and Fab_CD3_**14** were reduced (10 equiv of TCEP) and rebridged with 10 equiv of Br_2_PD-BCN **4** in a two-step procedure (4 h overall). Important to note that both these species were used fresh to avoid oxidation of the BCN moiety, thus Fab_CD3_-BCN **15** was only prepared after the intermediate purification step. These smaller equivalents and shorter reaction times still allowed the generation of the corresponding Fc_CD20_-(Tz)_2_**10**, Fab_HER2_-BCN **11**, and Fab_CD3_-BCN **15** species with full conversion and high purity as confirmed by LC-MS (Figure 3/G–I). The initial click reaction was carried out by mixing 50 nmol of Fc_CD20_-(Tz)_2_**10** with 30 nmol of Fab_HER2_-BCN **11** (0.6 equiv), which was introduced in substoichiometric quantity in order to favor the monoaddition product Fc_CD20_-(Fab_HER2_)-Tz **12**. After 16 h of incubation at 37 °C, SDS-PAGE analysis confirmed the generation of a protein of the expected size of Fc_CD20_-(Fab_HER2_)-Tz **12** but also some unwanted presence of Fc_CD20_-(Fab_HER2_)_2_**2**, as well as left over Fc_CD20_-(Tz)_2_**10** and Fab_HER2_-BCN **11** (Figure 3/E).

SEC purification allowed for the isolation of Fc_CD20_-(Fab_HER2_)-Tz **12**, although with some unwanted Fc_CD20_-(Fab_HER2_)_2_ SynAb **2** remaining according to SDS-PAGE (Figure 3/E). N.B.: Due to the overly high concentration of the gel loading we believe (based on LC-MS analysis, Figure 3/J, see SI for complete spectrum, showing no other species in the 50–100 kDa range) the product is eluting over two bands, and the “satellite” band beneath the product is thus not an impurity. Carrying out the purification on a larger SEC column should improve the separation of these species. Even so, SynAb **2** did not appear in the LC-MS spectrum where the Fc_CD20_-(Fab_HER2_)-Tz **12** intermediate appeared to be by far the major species (Figure 3/J, see SI for complete spectrum). Purified Fc_CD20_-(Fab_HER2_)-Tz **12** was then mixed with an excess of freshly prepared Fab_CD3_-BCN **15** (1.3 equiv) in water at 37 °C for 16 h. Satisfyingly, SEC purification of the crude mixture allowed for isolation of the desired bispecific Fc_CD20_-(Fab_HER2_)-Fab_CD3_ SynAb **13** as confirmed by SDS-PAGE and LC-MS analysis (Figure 3/K–M) in 12% yield (calculated from Fab_HER2_**8**). SDS-PAGE analysis did show some large molecular weight impurities, but these did not appear in the LC-MS spectrum (see SI for full spectrum). The additional peak visible in the LC-MS spectrum is due to Fab_CD3_ being digested to two species with a single amino acid difference by papain (see SI for details). To the best of our knowledge, this result constitutes the first example of an IgG-like bsAb produced via purely chemical methods from the corresponding Fab and Fc fragments. The overall process including protein digestion, protein reduction, and rebridging, both protein–protein click reactions and purifications, was carried out in only 5 days. Importantly, due to the highly modular nature of this method, it could easily be adapted to generate many other Fc_Z_-(Fab_X_)-Fab_Y_ IgG-like bispecific SynAbs with various Fab/Fc combinations. Only the initial parent antibodies would need to be changed, but all other steps (reduction, rebridging, protein–protein click) would still function, likely requiring only minor or no optimization. The speed and modularity of this chemical method to produce IgG-like bsAbs could make it a complement (or perhaps even alternative) to bioengineering after further optimization for yield and purity, with characteristics particularly suited for high-throughput screening and quick identification of hits.

### Biological Evaluation of SynAb **2** and Bispecific SynAb **13**

**Figure 4:**
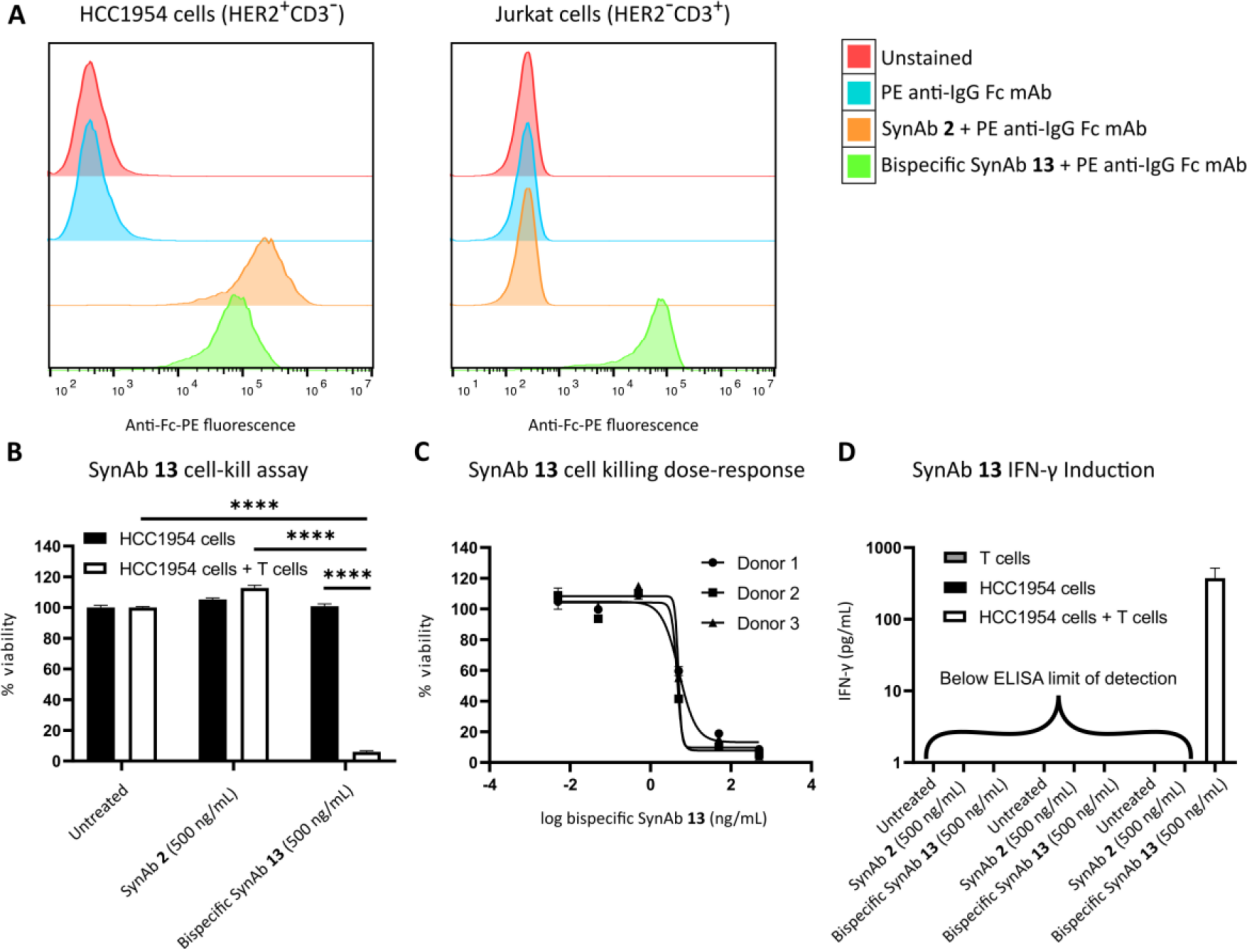
Biological testing of SynAbs 2 and 13. **A** Binding of SynAb **2** and bispecific SynAb **13** to HCC1954 (HER2^+^CD3^–^) and Jurkat (HER2^–^CD3^+^) cells. Flow cytometry histograms representative of three independent experiments. Cells were incubated with SynAb **2** or bispecific SynAb **13** followed by incubation with PE-labeled anti-IgG Fc antibody. PE fluorescence was measured by flow cytometry. **B** Cytotoxicity assay of SynAb **2** and bispecific SynAb **13**. HCC1954 (HER2^+^CD3^–^) cells alone or HCC1954/T cell cocultures (E:T ratio of 10:1) were either not treated or incubated with 500 ng/mL (3.4 nM) SynAb **2** or 500 ng/mL (3.4 nM) bispecific SynAb **13**. HCC1954 viability was assessed by CellTiter-Glo at 48 h following treatment. Data from three biologically independent experiments (three different blood donors) with three replicates each. **C** Cytotoxicity dose–response curve of bispecific SynAb **13**. HCC1954/T cell cocultures (E:T ratio of 10:1) were incubated with varying concentrations of bispecific SynAb **13** (serial dilutions ranging from 0.005 to 500 ng/mL, 0.034 pM to 3.4 nM). HCC1954 viability was assessed by CellTiter-Glo at 48 h following treatment. Data from three biologically independent experiments (three different blood donors) with three replicates each. **D** Induction of IFN-γ production by bispecific SynAb **13**. T cells alone, HCC1954 (HER2^+^CD3^–^) cells alone, or HCC1954/T cell cocultures (E:T ratio of 10:1) were either not treated or treated with 500 ng/mL (3.4 nM) SynAb **2** or 500 ng/mL (3.4 nM) bispecific SynAb **13**. Culture supernatant IFN-γ was quantified by ELISA at 48 h following treatment. Data from three biologically independent experiments (three different blood donors) with three replicates each. Data represented as means + SEM. For statistical analysis, two-way ANOVA was used followed by posthoc Tukey’s honestly significant difference multiple comparisons test with multiplicity-adjusted P values with α = 0.05. ****P < 0.0001. Curves in **C** fitted with nonlinear regression model “Sigmoidal, 4PL, X is log(concentration)”.

To further validate our chemical method for production of IgG-like proteins, we evaluated the biological functions of the Fc_CD20_-(Fab_HER2_)_2_ SynAb **2** and bispecific Fc_CD20_-(Fab_HER2_)-Fab_CD3_ SynAb **13** in binding and cell viability assays *in vitro*. Binding of both constructs to HCC1954 (HER2^+^CD3^–^) cancer cells and Jurkat (CD3^+^HER2^–^) cells was investigated by flow cytometry (three independent experiments in both cases, Figure 4/A). SynAb **2** or bispecific SynAb **13** compounds were incubated with HCC1954 (HER2^+^CD3^–^) cells and then stained with PE-labeled anti-IgG Fc. PE fluorescence was analyzed via flow cytometry. The assay revealed no increase in fluorescence for cells incubated with PE-labeled anti-IgG Fc alone in comparison to untreated cells, while a clear increase in fluorescence was observed in the case cells that were preincubated with either SynAb. This experiment indicated that both antibody constructs exhibited binding to HER2^+^ cells, as expected. Not unexpectedly, the monospecific Fc_CD20_-(Fab_HER2_)_2_ SynAb **2** exhibited increased binding to HER2^+^ cells compared to the bispecific SynAb **13**. This observation could be explained by the monospecific construct being bivalent for HER2-binding as opposed to the bispecific construct which is monovalent. Thus, the avidity effect would dictate stronger binding of the bivalent but monospecific SynAb **2.** These results confirm that the HER2 binding capacity of the constructs is retained after the digestion, reduction, rebridging, and assembly steps used during SynAb production. While the function of the Fc moiety was not directly tested, the results show that it retains its epitope for the PE-labeled anti-IgG Fc, the secondary antibody used for fluorescent detection of the constructs. As we have previously demonstrated that natively rebridging the Fc of an IgG1 with PDs does not affect FcRn-binding, CD16a kinetics, and ADCC activity, no change in the Fc-activity of SynAb **2** is expected.^40^

Binding to Jurkat (CD3^+^HER2^–^) cells was carried out under similar conditions, by incubating cells with SynAb **2** or bispecific SynAb **13**, followed by staining with PE-labeled anti-IgG Fc (Figure 4/A). As expected, only the bispecific Fc_CD20_-(Fab_HER2_)-Fab_CD3_ SynAb **13** induced an increase in fluorescence when compared to untreated cells, confirming the capacity of the bispecific SynAb **13** to bind to CD3^+^ cells, driven by the presence of the Fab_CD3_ moiety present in the construct. Again, these results suggest that the affinity of the Fab_CD3_ for the CD3 receptor was not abrogated by previous digestion, reduction, rebridging, and assembly steps. Monospecific Fc_CD20_-(Fab_HER2_)_2_ SynAb **2** staining, on the contrary, and as expected, led to no increase in PE fluorescence compared to untreated cells, due to the lack of any CD3 binding module.

After validation of the target-binding ability of the SynAb constructs, the capacity of the bispecific SynAb **13** to recruit T cells to HCC1954 (HER2^+^) cells and induce T cell-mediated cell death was evaluated on three blood donor samples. T cell/HCC1954 cocultures (E:T ratio of 10:1) or HCC1954 monocultures were treated with 500 ng/mL of bispecific SynAb **13** or SynAb **2** (as a control), or not treated, and HCC1954 viability was assessed after 48 h. As expected, cell viability did not decrease for cells treated with Fc-(Fab_HER2_)_2_ SynAb **2** with or without T cells, and for Fc-(Fab_HER2_)-Fab_CD3_ bispecific SynAb **13** without T cells, when compared to untreated cells. On the contrary, to our delight, HCC1954 cell viability was reduced when both bispecific SynAb **13** and T cells were present (Figure 4/B). This reduction in viability was also confirmed to be dose-dependent (Figure 4/C). This suggests that the construct exhibited T cell engagement activity through simultaneous binding of CD3 and HER2 in trans (i.e., on different cells). To further confirm that target cell-killing was due to increased T cell activation, expression of IFN-γ (a T cell activation marker) was evaluated. For this purpose, T cell/HCC1954 cocultures (E:T ratio of 10:1), HCC1954 monocultures, or T cell monocultures were treated with 500 ng/mL bispecific SynAb **13** or SynAb **2.** Culture supernatant IFN-γ was quantified by ELISA at 48 h following treatment (Figure 4/D). As expected, only the condition where bispecific SynAb **13** was incubated with HCC1954 cells cocultured with T cells induced expression of IFN-γ confirming that the construct drove T cell activation in the presence of target cells. It is also important to note that no IFN-γ production was observed when the construct was incubated with T cells alone, suggesting that immune activation would be primarily localized to the tumor environment, decreasing the potential for side effects, such as cytokine release syndrome (CRS), and increasing the therapeutic window.^54^ Based on the three blood donor samples, the HCC1954 cell viability IC50 for bispecific SynAb **13** was determined to be 4.9 ± 0.2 ng/mL (33 ± 1 pM). This value is similar to IC50 values reported in the literature for engineered (HER2 × CD3) TCEs—with or without an Fc—and evaluated *in vitro* with a similar E:T ratio on high HER2 expressing cell lines such as SKBR3, BT-474, Colo205-luc, and SKOV-3 cell lines (reported IC_50_ vary from 6 to 60 pM).^38,39,55,56^ Overall, these biological results demonstrate potent TCE activity for the chemically produced bispecific SynAb **13**.

## Conclusion and Perspectives

We have described a novel, modular, and rapid, purely chemical approach for the assembly of IgG-like constructs, dubbed SynAbs, from parent Fc and Fab modalities. This first-in-class strategy allows for the generation of both mono- and bispecific constructs, via pyridazinedione-mediated disulfide rebridging followed by Cu-free click chemistry for protein–protein ligation without the need for any protein engineering. As an example, an IgG-like bispecific T cell engager SynAb, Fc_CD20_-(Fab_HER2_)-Fab_CD3_**13**, was generated in only 5 days starting from commercial parent anti-CD20, anti-HER2, and anti-CD3 mAbs. Biological evaluation of the bispecific SynAb **13** confirmed the preservation of the binding capacities of the Fab_HER2_ and Fab_CD3_ modalities for their respective HER2 and CD3 receptors, while the Fc moiety was recognized by an anti-IgG Fc antibody. Prior work suggests that the activity of the Fc is not impaired by PD-modification.^40^ Importantly, the construct was able to redirect T cells to HCC1954 (HER2^+^) cancer cells to induce T cell activation and T cell-dependent cancer cell death (IC_50_ = 4.9 ± 0.2 ng/mL). The method could be applied to any mAb with a single solvent-accessible disulfide in the Fab region, to investigate a wide range of Fab pairs as targeted arms for an IgG-like bsAb. This versatility, combined with the speed and site-selectivity of the method could make it a valuable tool for the high-throughput production of a wide range of IgG-like bsAbs, with the potential to speed up the screening and hit identification processes. Certainly, the method is in its early stages and would need further optimization of yield and purity, and there is also room for further improvements/additions to the strategy. The linkers between the components could be altered/tuned (e.g., length, rigidity, etc.) to assess effect on activity, and cleavage could be introduced between the component proteins. Additionally, the constructs would need not be limited to construction solely from antibody fragments—the Fc could be used as a platform to attach additional functionality through click-chemistry such as immunomodulatory enzymes (e.g., sialidase),^57^ cytokines (e.g., IL2),^58^ or immunomodulatory molecules (e.g., CTLA-4)^59^ to unlock novel mechanisms of action. Furthermore, IgG-like bsAb-payload conjugates—Fc_Z_-(Fab_X_-payload)-Fab_Y_-payload—can also be envisioned if a Br_2_PD having two orthogonal click handles (e.g., Br_2_PD-(BCN)-DBCO) is used to rebridge the Fab moieties. This would give access to a postassembly functionalization step via a click reaction (e.g., with an azide-linked small payload) to introduce drugs and/or fluorophores on the IgG-like bispecific construct. As a further potential improvement to the method, the synthesis of an asymmetric, bifunctional bis-Br_2_PD linker for the rebridging of the Fc is under investigation in our group, in order to allow for the carrying out of the three-protein assembly in one orthogonally controlled step. Moving away from sequential assembly this way would help avoid the intermediate SEC purification step. We believe that the numerous assets of the described chemical method could make it a valuable complementary tool to bioengineering, especially for the initial, high-throughput, low scale screening stages of IgG-like bsAb discovery. While scalability remains to be explored, its validation could highlight this chemical method as an attractive alternative to bioengineering for the production of IgG-like bsAbs, due to the rapidity and modularity it affords, as well as the potential for an in-built platform for further bsAb functionalization. In the meantime, the SynAb strategy constitutes a quick and versatile way to access IgG-like bsAbs for researchers across disciplines having a restricted access to bioengineering technology. Hopefully, this approach will be fertile ground for the future production and evaluation of numerous IgG-like bsAb, IgG-like bsAb–payload conjugates and Fc-bearing antibody–protein conjugates and assist in the discovery of new therapies.

## Supporting Information Available

Synthetic chemistry experimental details, including synthetic procedures and compound characterizations. Chemical biology experimental details, including bioconjugation procedures, details on antibody digestion and LC-MS methodology. Biological evaluation experimental details including details on statistical analysis. ^1^H and ^13^C NMR spectra. Full LC-MS spectra including TIC and raw data (PDF)
