# Supplementary material for: Modular Chemical Construction of IgG-like Mono- and Bispecific Synthetic Antibodies (SynAbs)
Source: ACS Cent Sci. 2023 Feb 21;9(3):476–87. doi: 10.1021/acscentsci.2c01437 (PMC10037451; doi:10.1021/acscentsci.2c01437)

Electronic Supporting Information: Modular chemical construction of IgG-like mono- and bispecific synthetic antibodies (SynAbs)

Fabien Thoreau,[a,†] Peter A. Szijj,[a,†] Michelle K. Greene,[b] Léa N. C. Rochet,[a] Ioanna A. Thanasi,[a] Jaine K. Blayney,[b] Antoine Maruani,[a] James R. Baker,[a*] Christopher J. Scott,[b*] Vijay Chudasama[a*]

a: Department of Chemistry, University College London, 20 Gordon Street, London, WC1H 0AJ, UK.

b: Patrick G Johnston Centre for Cancer Research, School of Medicine, Dentistry and Biomedical Sciences, Queen's University Belfast, Belfast, UK.

*: Correspondence: v.chudasama@ucl.ac.uk, c.scott@qub.ac.uk, j.r.baker@ucl.ac.uk

†: These authors contributed equally to this work. As F.T. and P.S. contributed equally to this work, they are permitted to list their names as first on the author list on any C.V. or grant and fellowship application, etc. Their names were merely listed in this order arbitrarily, determined by a game of Keyforge.

Synthesis of bis-PD(Tz)$_2$ **9**

First, di-*tert*-butyl hydrazine-1,2-dicarboxylate was mono-alkylated using an excess of propargyl bromide in a phase transfer reaction to yield compound **S10** in 80% yield (Scheme S1). Compound **S10** was then reacted with a slight excess (1.5 eq.) of 1,4-dibromobutane to yield protected hydrazine dimer **S11** in 34% yield. *In situ* Boc deprotection and acylation of hydrazine dimer **S11** upon reaction in acetic acid with 2,3-dibromomaleic acid afforded desired bis-PD linker **S12** in 93% yield after purification. In house synthesis of Me-tetrazine-PEG$_3$-N$_3$ **S5** (five steps, see ESI for details), based on previously described procedure,[1,2] enabled subsequent double functionalisation of compound **S12** *via* CuAAC to afford the dually tetrazine-modified bis-PD(Tz)$_2$ **9** in 17% yield (Scheme S1).

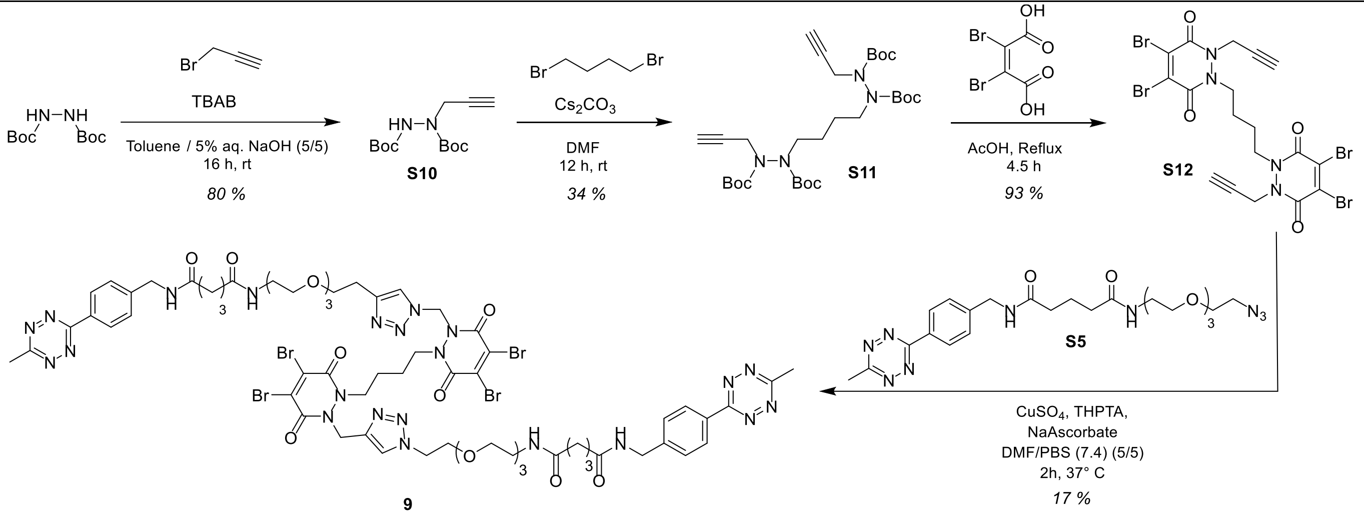

Scheme S1 | **Synthetic route for the generation of "bis-PD(Tz)$_2$" 9, a linker encompassing two dibromopyridazinedione platforms each bearing a tetrazine handle.**

Experimental Section

1. Synthetic chemistry section

1.1 General experimental details for synthetic chemistry

Chemicals were purchased from Sigma-Aldrich, Santa Cruz Biotechnology, or AlfaAesar, and were used as received unless otherwise stated. Solvents were used as supplied. Where described below, petrol refers to petroleum ether (b.p. 40-60 °C). All reactions were monitored by thin-layer chromatography (TLC) on pre-coated silica gel plates. Flash column chromatography was carried out with either pre-loaded Biotage® SNAP column chromatography cartridges or pre-loaded GraceResolv™ flash cartridges on a Biotage® Isolera Spektra One flash chromatography system. All reaction mixtures were stirred magnetically unless stated otherwise. Al reactions involving moisture sensitive compounds or procedures were carried out in flame-dried flask under an atmosphere of argon. Room temperature (RT) is defined as 16-23 °C. Reactions at 0 °C were cooled with an ice/water bath. Removal of solvent and concentration *in vacuo* was carried out on a Büchi rotary evaporator followed by evaporation under high vacuum.

^{1}H NMR spectra were obtained at 400, 500, 600 or 700 MHz. ^{13}C NMR spectra were obtained at 100, 125, 150 or 175 MHz. All results were obtained using Bruker NMR instruments, the models are as follows: Avance III 400, Avance 500, Avance III 600, Avance Neo 700. The chemical shifts (δ) for ^{1}H and ^{13}C are quoted relative to residual signals of the solvent on the ppm scale. ^{1}H NMR peaks are reported as singlet (s), doublet (d), triplet (t), quint. (quintet), m (multiplet), br. (broad), dd (doublet of doublet). Coupling constants (J values) are reported in Hertz (Hz) and are H-H coupling constants unless otherwise stated. In the case of amide rotamers, and when possible, only the major rotamer has been assigned for chemical shifts, and areas underneath all rotameric peaks have been considered for integration calculations. Peak assignments were carried out with the aid of ^{1}H COSY and ^{1}H–^{13}C HSQC experiments where necessary. Infrared spectra were obtained on a Perkin Elmer Spectrum 100 FTIR Spectrometer operating in ATR mode. For synthetic products mass spectra were obtained from the UCL mass spectrometry service on a Thermo Orbitrap Exactive Plus (ESI) mass spectrometer.

1.2 Compound **S1**, *tert*-butyl (4-cyanobenzyl)carbamate[3]

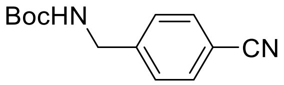

To a stirring solution of NaOH (3.6 g, 89.1 mmol) and di-*tert*-butyl dicarbonate (7.1 g, 32.6 mmol) in H_2O (30 mL) was added, at room temperature, a pre-dissolved solution of 4-(aminomethyl)benzonitrile (5.0 g, 29.7 mmol) in H_2O (30 mL). The mixture was stirred for 16 h, after which time a white precipitate had

formed. The mixture was then filtered, the solid washed with H_2O (100 mL), and the resulting solid dried under vacuum to yield compound **S1** as a white solid (6.09 g, 26.2 mmol, 88%). ^{1}H NMR (400 MHz, $CDCl_3$) δ 7.62 (d, *J* = 8.3, 2H), 7.38 (d, *J* = 8.3, 2H), 4.96 (br s, 1H), 4.37 (d, *J* = 5.9 Hz, 2H), 1.46 (s, 9H); ^{13}C NMR (100 MHz, $CDCl_3$) δ 144.7 (C), 132.5 (C), 127.9 (C), 118.8 (C), 111.1 (C), 80.1 (C), 44.3 (CH_2), 28.4 (CH_3); IR (solid) 3350, 2974, 2927, 2226, 1692 cm^{-1}.

1.3 Compound **S2**, *tert*-butyl (4-(6-methyl-1,2,4,5-tetrazin-3-yl)benzyl)carbamate[4]

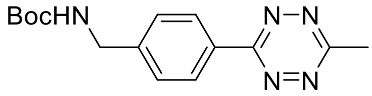

The following procedure was adapted from work by Lang *et al.*[3] To a stirring suspension of *tert*-butyl carbamate **S1** (3.0 g, 12.9 mmol), acetonitrile (6.72 mL, 12.9 mmol), and $Zn(OTf)_2$ (2.34 g, 6.46 mmol) in 1,4-dioxane (6 mL) was added, at room temperature, hydrazine hydrate (80% w/w, 39.5 mL, 646 mmol). The reaction was heated to 65 °C and stirred for 72 h. After this time, the reaction was cooled to room temperature and diluted with EtOAc (50 mL). The mixture was washed with 1 M HCl (50 mL), and the aqueous phase extracted with EtOAc (2 × 30 mL). The organic phase was dried ($MgSO_4$), filtered and solvent was then removed *in vacuo*. The resulting crude residue was dissolved in a mixture of DCM and acetic acid (1:1, 200 mL), and to this was added $NaNO_2$ (17.8 g, 258 mmol) slowly over a period of 15 min, during which time the reaction turned bright red. The reaction was then diluted with DCM (200 mL), washed with sodium bicarbonate (sat. aq., 200 mL) and the aqueous phase extracted with DCM (2 × 100 mL). The organic phases were combined and then dried ($MgSO_4$), filtered and the solvent was then removed *in vacuo*. The resulting residue was purified by flash column chromatography (20% EtOAc/petrol) to yield tetrazine **S2** as a pink solid (1.07 g, 3.55 mmol, 28%). ^{1}H NMR (400 MHz, $CDCl_3$) δ 8.55 (d, *J* = 8.4 Hz, 2H), 7.50 (d, *J* = 8.3 Hz, 2H), 4.97 (br s, 1H), 4.44 (d, *J* = 5.8 Hz, 2H), 3.09 (s, 3H), 1.48 (s, 9H); ^{13}C NMR (100 MHz, $CDCl_3$) δ 167.3 (C), 164.0 (C), 144.0 (C), 130.1 (C), 128.3 (C), 128.1 (C), 80.1 (C), 28.5 (CH_2), 21.1 (CH_3); IR (solid) 3339, 2974, 2928, 1696, 1516 cm^{-1}.

1.4 Compound **S3**, 5-((4-(6-methyl-1,2,4,5-tetrazin-3-yl)benzyl)amino)-5-oxopentanoic acid[5]

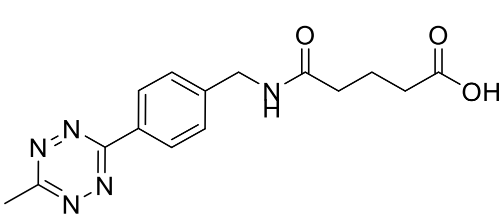

tert-Butyl (4-(6-methyl-1,2,4,5-tetrazin-3-yl)benzyl)carbamate **S2** (800 mg, 2.65 mmol) was dissolved in a mixture of TFA and DCM (1:4, 20 mL) and the solution was stirred at room temperature for 2 h. The solvent was then removed *in vacuo* and the mixture re-dissolved in THF (50 mL). To this solution was added

glutaric anhydride (605 mg, 5.31 mmol) and the mixture stirred at 55 °C for 16 h. The solvent was removed *in vacuo* and the mixture re-dissolved in sat. aq. K_2CO_3 solution (100 mL). The mixture was then acidified with 15% HCl aq. solution until the mixture stopped producing $CO_{2(g)}$ on addition of acid. The mixture was then extracted with EtOAc (3 × 50 mL) and the combined organic phases washed with H_2O (4 × 30 mL) and brine (30 mL), and then dried ($MgSO_4$). Any precipitate formed during extraction was re-dissolved in sat. aq. K_2CO_3 solution (30 mL) and the work-up was repeated on this solution and the dried organic phases were combined, filtered and the solvent removed *in vacuo* to yield compound **S3** as a purple powder (691 mg, 2.2 mmol, 83%) without further purification. ^{1}H NMR (400 MHz, $CDCl_3$) δ 8.41 (d, *J* = 8.4, 2H), 7.51 (d, *J* = 8.5 Hz, 2H), 4.38 (d, *J* = 6.0 Hz, 2H), 2.98 (s, 3H), 4.27 (q, *J* = 7.4 Hz, 4H), 1.76 (quint., *J* = 7.4 Hz, 2 H); ^{13}C NMR (100 MHz, $CDCl_3$) δ 174.2 (C), 171.9 (C), 167.1 (C), 163.2 (C), 144.5 (C), 130.4 (C), 128.0 (C), 127.5 (C), 41.9 (CH_2), 34.4 (CH_2), 33.0 (CH_2), 20.8 (CH_3), 20.7 (CH_2); IR (solid) 3271, 3025, 2973, 2923, 2880, 1694, 1630, 1523 cm^{-1}.

1.5 Compound **S4**, *N*^{1}-(3-(2-(2-(3-aminopropoxy)ethoxy)ethoxy)propyl)-*N*^{5}-(4-(6-methyl-1,2,4,5-tetrazin-3-yl)benzyl)glutaramide[6]

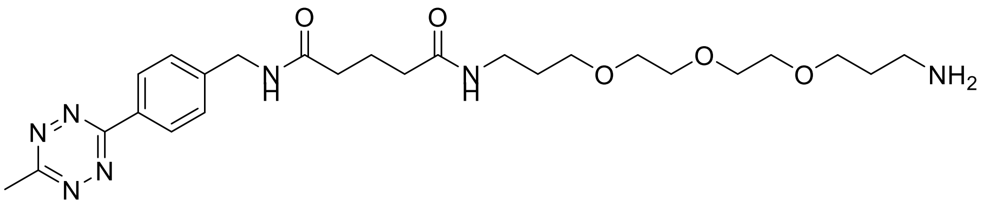

To a solution of *O,O'*-bis(3-aminopropyl)diethylene glycol (280 mg, 1.27 mmol) in DCM (5 mL) was added slowly, at room temperature, a solution of 5-((4-(6-methyl-1,2,4,5-tetrazin-3-yl)benzyl)amino)-5-oxopentanoic acid **S3** (200 mg, 0.63 mmol), HATU (240 mg, 0.63 mmol), and NEt_3 (64 mg, 0.63 mmol) in DCM (5 mL). The resulting solution was stirred at room temperature for 16 h. The solvent was then removed *in vacuo*, and the mixture re-dissolved in 1 M HCl solution (20 mL) and washed with DCM (3 × 20 mL) to remove unreacted 5-((4-(6-methyl-1,2,4,5-tetrazin-3-yl)benzyl)amino)-5-oxopentanoic acid **S3**. The aqueous phase was then basified with sat. aq. K_2CO_3 solution until $CO_{2(g)}$ evolution stopped, and then extracted with DCM (3 × 20 mL). The combined organic phases were extracted with 1 M HCl solution (20 mL). The aqueous phase was basified with sat. aq. K_2CO_3 solution until $CO_{2(g)}$ evolution stopped, and then extracted with DCM (3 × 20 mL). The combined organic phases were washed with brine (20 mL), dried ($MgSO_4$), filtered and the solvent removed *in vacuo*. The crude residue was purified by flash column chromatography (10–30% MeOH in DCM) to afford compound **S4** (47.6 mg, 0.09 mmol, 15%) as a purple oil. ^{1}H NMR (600 MHz, $CDCl_3$) δ 8.49 (d, *J* = 8.4 Hz, 2H), 8.35 (t, *J* = 5.8 Hz, 1H), 7.57 (d, *J* = 8.5 Hz, 2H), 6.67 (t, *J* = 6.1 Hz, 1H), 4.52 (d, *J* = 6.2 Hz, 2H), 3.71 (t, *J* = 5.3 Hz, 2H), 3.65–3.61

(m, 4H), 3.61–3.57 (m, 4H), 3.50 (t, J = 5.6 Hz, 2H), 3.32 (q, J = 6.8 Hz, 2H), 3.19–3.15 (m, 2H), 3.08 (s, 3H), 2.40 (t, J = 7.5 Hz, 2H), 2.30 (t, J = 6.9 Hz, 2H), 2.03–1.96 (m, 4H), 1.77 (quint., J = 6 Hz, 2H); ^{13}C NMR (150 MHz, $CDCl_3$) δ 174.0 (C), 173.3 (C), 167.2 (C), 164.2 (C), 144.6 (C), 130.4 (C), 128.8 (CH), 128.1 (CH), 70.9 (CH_2), 70.7 (CH_2), 69.8 (CH_2), 69.7 (CH_2), 69.6 (CH_2), 68.3 (CH_2), 43.0 (CH_2), 40.7 (CH_2), 36.5 (CH_2), 35.4 (CH_2), 35.1 (CH_2), 30.0 (CH_2), 26.1 (CH_2), 21.9 (CH_2), 21.3 (CH_3). IR (thin film) 3302, 2945, 2831, 1642, 1630, 1542 cm^{-1}.

1.6 Compound **S5**, N^1-(2-(2-(2-(2-azidoethoxy)ethoxy)ethoxy)ethyl)-N^5-(4-(6-methyl-1,2,4,5-tetrazin-3-yl)benzyl)glutaramide[2]

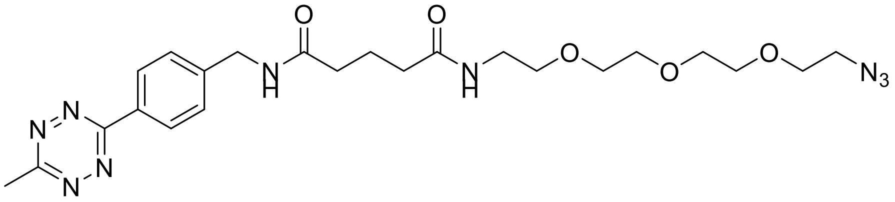

To a solution of 5-((4-(6-methyl-1,2,4,5-tetrazin-3-yl)benzyl)amino)-5-oxopentanoic acid **S3** (0.20 g, 0.63 mmol) in DCM (5 mL) was added, at room temperature, HATU (0.24 g, 0.63 mmol), and NEt_3 (87.80 µL, 0.63 mmol), and the reaction stirred for 5 min. Subsequently, to this solution was added, at room temperature, a solution of 2-(2-(2-(2-azidoethoxy)ethoxy)ethoxy)ethan-1-amine (387.50 µL, 1.96 mmol) in DCM (5 mL), and the resulting solution was stirred at room temperature for 16 h. The reaction was then diluted with EtOAc (25 mL) and H_2O (25 mL) and the phases separated. The aqueous phase was extracted with EtOAc (3 × 25 mL) and the combined organic phases were washed with H_2O (3 × 25 mL), brine (20 mL), dried ($MgSO_4$), filtered and the solvent removed *in vacuo*. The crude residue was purified by flash column chromatography (0-10% MeOH in DCM) to afford N^1-(2-(2-(2-(2-azidoethoxy)ethoxy)ethoxy)ethyl)-N^5-(4-(6-methyl-1,2,4,5-tetrazin-3-yl)benzyl)glutaramide **S5** (0.21 g, 0.51 mmol, 64%) as a purple solid. ^{1}H NMR (400 MHz, $CDCl_3$) δ 8.55 (d, J = 8.4 Hz, 2H), 7.51 (d, J = 8.5 Hz, 2H), 6.61 (br. s, 1H), 6.16 (br. s, 1H), 4.55 (d, J = 6.0 Hz, 2H), 3.68–3.59 (m, 10 H), 3.56–3.52 (m, 2H), 3.45–3.36 (m, 4H), 3.09 (s, 3H), 2.35 (t, J = 7.1 Hz, 2H), 2.26 (t, J = 6.9 Hz, 2H), 2.01 (quint., J = 6.9 Hz, 2 H); ^{13}C NMR (100 MHz, $CDCl_3$) δ 172.7 × 2 (C), 167.3 (C), 164.0 (C), 143.6 (C), 131.0 (C), 128.6 (CH), 128.3 (CH), 70.8 (CH_2), 70.7 (CH_2), 70.6 (CH_2), 70.3 (CH_2), 70.1(CH_2), 69.8 (CH_2), 50.7 (CH_2), 43.3 (CH_2), 39.3 (CH_2), 35.3 (CH_2), 35.2 (CH_2), 22.0 (CH_2), 21.2 (CH_3). IR (solid) 3298, 3076, 2868, 2101, 1637, 1541 cm^{-1}. HRMS (ESI) calcd for $C_{23}H_{34}N_9O_5$ $[M+H]^+$ 516.2677; observed 516.2677.

1.7 Compound **S6**, di-*tert*-butyl 1-methylhydrazine-1,2-dicarboxylate[7]

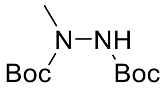

To a solution of methyl hydrazine (1.14 mL, 21.70 mmol) in propan-2-ol (16 mL), was added drop-wise over 30 min a solution of di-*tert*-butyl dicarbonate (11.40 g, 52.10 mmol, pre-dissolved in DCM (12 mL)). The reaction was then stirred at 21 °C for 16 h. After this time, the solvents were removed *in vacuo* and the crude residue purified by flash column chromatography (0% to 15% EtOAc/petrol) to afford di-*tert*-butyl-1-methylhydrazine-1,2-dicarboxylate **S6** (4.67 g, 19.10 mmol, 88%) as a white solid. ^{1}H NMR (400 MHz, $CDCl_3$, rotamers) δ 6.41–6.15 (m, 1H) 3.10 (s, 3H), 1.46–1.45 (m, 18H); ^{13}C NMR (150 MHz, $CDCl_3$, rotamers) δ 155.9 (C), 81.3 (C), 37.6 (CH_3), 28.3 (CH_3); IR (solid) 3299, 2974, 2929, 1703 cm^{-1}.

1.8 Compound **S7**, di-*tert*-butyl 1-(3-(*tert*-butoxy)-3-oxopropyl)-2-methylhydrazine-1,2-dicarboxylate[7]

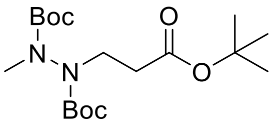

To a solution of di-*tert*-butyl 1-methylhydrazine-1,2-dicarboxylate **S6** (3.00 g, 12.20 mmol) in *tert*-butanol (15 mL) was added 10% aq. NaOH (0.5 mL) and the reaction mixture stirred at 21 °C for 10 min. After this time, *tert*-butyl acrylate (5.31 mL, 36.60 mmol) was added to the solution and the reaction mixture was heated at 60 °C for 24 h. Following this, the solvent was removed *in vacuo* and the crude residue was dissolved in EtOAc (150 mL) and washed with water (3 × 50 mL). The organic layer was then dried ($MgSO_4$), filtered and the solvent removed *in vacuo*. Purification of the crude residue by flash column chromatography (0% to 20% EtOAc/petrol) afforded di-*tert*-butyl-1-(3-(*tert*-butoxy)-3-oxopropyl)-2-methylhydrazine-1,2-dicarboxylate **S7** (3.33 g, 8.91 mmol, 73%) as a clear oil. ^{1}H NMR (600 MHz, $CDCl_3$, rotamers) δ 3.82–3.47 (m, 2H), 3.03–2.94 (m, 3H), 2.47 (t, J = 7.1 Hz, 2H), 1.48–1.37 (m, 27H); ^{13}C NMR (150 MHz, $CDCl_3$, rotamers) δ 171.0 (C), 155.4 (C), 154.4 (C), 81.0 (C), 44.6 (CH_3), 36.6 (CH_2), 34.1 (CH_2), 28.3 (CH_3); IR (thin film) 2974, 2931, 1709 cm^{-1}.

1.9 Compound **S8**, 3-(4,5-dibromo-2-methyl-3,6-dioxo-3,6-dihydropyridazin-1(2*H*)-yl)propanoic acid[7]

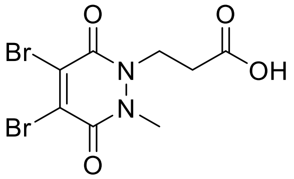

Dibromomaleic anhydride (4.00 g, 14.61 mmol) was dissolved in AcOH (80 mL) and heated under reflux for 30 min. To this solution was added di-*tert*-butyl 1-(3-(*tert*-butoxy)-3-oxopropyl)-2-methylhydrazine-1,2-dicarboxylate **S7** (4.77 g, 12.75 mmol) and the reaction heated under reflux for a further 4 h. The solvent was removed *in vacuo* by co-evaporation with toluene and the crude residue purified by flash column chromatography (50-100% EtOAc 1% AcOH/petrol) to yield 3-(4,5-dibromo-2-methyl-3,6-dioxo-3,6-dihydropyridazin-1(2*H*)-yl)propanoic acid **S8** (3.22 g, 9.00 mmol, 71%) as a yellow powder. ^{1}H NMR (700 MHZ, $CDCl_3$) δ 4.41 (t, *J* = 7.1 Hz, 2H), 3.69 (s, 3H), 2.77 (t, *J* = 7.1 Hz, 2H); ^{13}C NMR (150 MHz, $CDCl_3$) δ 173.9 (C), 153.5 (C), 153.2 (C), 136.4 (C), 135.7 (C), 43.5 (C), 35.3 (C), 31.2 (C); IR (solid) 3300–2700, 2924, 1726, 1617, 1570, 1442, 1396 cm^{-1}.

1.10 Compound **S9**, 2,5-dioxopyrrolidin-1-yl 3-(4,5-dibromo-2-methyl-3,6-dioxo-3,6-dihydropyridazin-1(2*H*)-yl)propanoate[7]

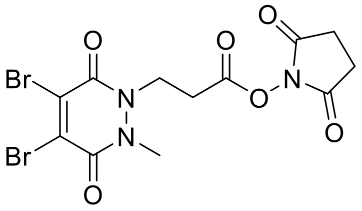

To a solution of 3-(4,5-dibromo-2-methyl-3,6-dioxo-3,6-dihydropyridazin-1(2*H*)-yl)propanoic acid **S8** (2.00 g, 5.62 mmol) in dry THF (40 mL) at 0 °C was added DCC (1.28 g, 6.20 mmol). The solution was stirred at 0 °C for 30 min. Following this, was added NHS (718 mg, 6.2 mmol) and the reaction stirred at room temperature for a further 16 h. The solvent was then removed *in vacuo* and the crude residue purified by flash column chromatography (20-100% EtOAc/petrol) to yield pyridazinedione **S9** (0.80 g, 1.76 mmol, 31%) as a white powder. ^{1}H NMR (700 MHZ, $CDCl_3$) δ 4.48 (t, J= 6.9, 2H), 3.68 (s, 3H), 3.10 (t, *J* = 6.9, 2H), 2.85 (br. s, 4H); ^{13}C NMR (175 MHz, $CDCl_3$) δ 168.7 (C), 166.0 (C), 153.3 (C), 153.1 (C), 136.9 (C), 135.5 (C), 43.5 (CH_2), 35.3 (CH_3), 30.5 (CH_2), 25.7 (CH_2); IR (solid) 2927, 2851, 1733, 1632, 1571, 1203 cm^{-1}.

1.11 Compound **3**, *N*[1]-(17-(4,5-dibromo-2-methyl-3,6-dioxo-3,6-dihydropyridazin-1*(2H)-yl*)-15-oxo-4,7,10-trioxa-14-azaheptadecyl)-*N*[5]-(4-(6-methyl-1,2,4,5-tetrazin-3-yl)benzyl)glutaramide[6]

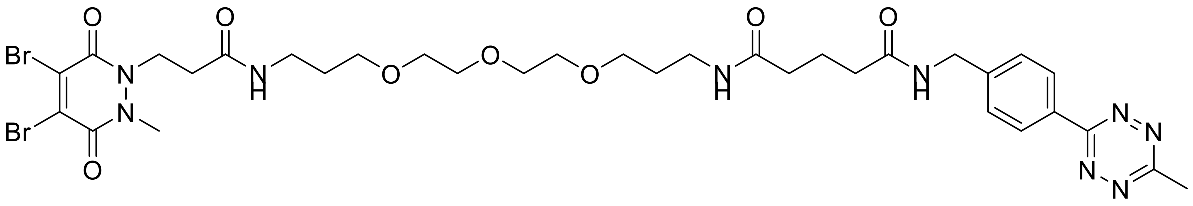

To a solution of 2,5-dioxopyrrolidin-1-yl 3-(4,5-dibromo-2-methyl-3,6-dioxo-3,6-dihydropyridazin-1(2*H*)-yl) propanoate **S9** (61.2 mg, 140.0 µmol) in DCM (5 mL), was added N^1-(3-(2-(2-(3-aminopropoxy)ethoxy)ethoxy)propyl)-N^5-(4-(6-methyl-1,2,4,5-tetrazin-3-yl)benzyl)glutaramide **S4** (27.6 mg, 58.0 µmol) and NEt_3 (7.4 µL, 5.4 mg, 53.0 µmol), and the reaction mixture was stirred at room temperature for 3 h. After this time, the reaction was concentrated *in vacuo* and the crude residue dissolved in $CHCl_3$ (25 mL) and washed with water (2 × 15 mL) and sat. aq. K_2CO_3 (15 mL). The organic layer was then dried ($MgSO_4$), filtered and the solvent removed *in vacuo* . Purification of the crude residue by flash column chromatography (5% to 20% MeOH/EtOAc) afforded compound **3** (15.8 mg, 18.0 µmol, 32%) as a purple oil. ^{1}H NMR (600 MHz, $CDCl_3$) δ 8.53 (d, *J* = 8.4 Hz, 2H), 7.50 (d, *J* = 8.5 Hz, 2H), 7.04 (t, *J* = 5.2 Hz, 1H), 6.80 (t, *J* = 5.8 Hz, 1H), 6.46 (t, *J* = 5.3 Hz, 1H), 4.55 (d, *J* = 6.0 Hz, 2H), 4.39 (t, *J* = 6.9 Hz, 2H), 3.69 (s, 3H), 3.63–3.50 (m, 12H), 3.36–3.26 (m, 4H), 3.09 (s, 3H), 2.80 (d, *J* = 4.8 Hz, 1H), 2.56 (t, *J* = 6.9 Hz, 2H), 2.36 (t, *J* = 7.3 Hz, 2H), 2.26 (t, *J* = 6.9 Hz, 2H), 1.99 (app. quint., *J* = 7.1 Hz, 2H), 1.78–1.69 (m, 4H); ^{13}C NMR (150 MHz, $CDCl_3$) δ 173.0 (C), 172.8 (C), 169.2 (C), 167.4 (C), 164.0 (C), 153.1 (C), 152.9 (C), 143.6 (C), 136.4 (C), 135.4 (C), 131.0 (C), 128.6 (2×CH), 128.3 (2×CH), 70.6 (2×CH_2), 70.1 (2×CH_2), 70.0 (2×CH_2), 44.6 (CH_2), 43.3 (CH_2), 38.2 (CH_2), 38.0 (CH_2), 35.6 (CH_2), 35.4 (CH_2), 35.2 (CH_3), 34.1 (CH_2), 29.1 (CH_2), 28.9 (CH_2), 22.2 (CH_2), 21.3 (CH_3); IR (thin film) 3310, 2923, 2851, 1734, 1631, 1543 cm^{-1}; LRMS (ESI). 858 (50, [M^{81}Br^{81}Br+H]$^+$), 856 (100, [M^{79}Br^{81}Br+H]$^+$), 854 (50, [M^{79}Br^{79}Br+H]$^+$); HRMS (ESI) calcd for $C_{33}H_{46}Br_2N_9O_8Na$ [M^{79}Br^{81}Br+Na]$^+$ 878.1630; observed 878.1639.

1.12 Compound **4**, ((1R,8S,9s)-bicyclo[6.1.0]non-4-yn-9-yl)methyl (2-(2-(2-(3-(4,5-dibromo-2-methyl-3,6-dioxo-3,6-dihydropyridazin-1*(2H)*-*yl*)propanamido)ethoxy)ethoxy)ethyl)carbamate[7]

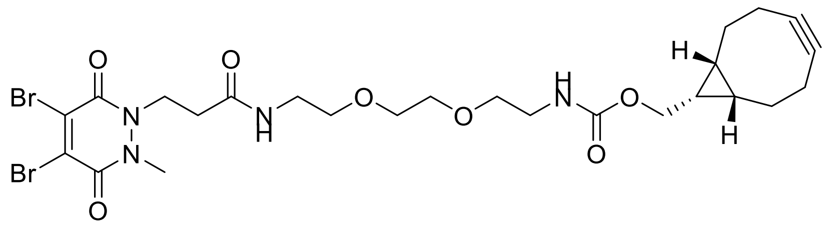

To a solution of 2,5-dioxopyrrolidin-1-yl-3-(4,5-dibromo-2-methyl-3,6-dioxo-3,6-dihydropyridazin-1(*2H*)-*yl*) propanoate **S9** (45.0 mg, 96.0 µmol) in acetonitrile (10 mL) was added *N*-[(1*R*, 8*S*, 9*S*)-bicyclo[6.1.0]non-4-yn-9-ylmethyloxycarbonyl]-1,8-diamino-3,6-dioxaoctane (21.0 mg, 64.0 µmol) and the reaction stirred at 21 °C for 16 h. After this time, solvent was removed *in vacuo* and the crude residue dissolved in $CHCl_3$ (30 mL) and washed with water (2 × 20 mL) followed by saturated aq. K_2CO_3 (20 mL). The organic layer was then dried ($MgSO_4$) and concentrated *in vacuo.* Purification of the crude residue by flash column chromatography (0 to 20% MeOH/EtOAc) afforded ((1*R*, 8*S*, 9*S*)-bicyclo[6.1.0]non-4-yn-9-yl)methyl(2-(2-(2-(3-(4,5-dibromo-2-methyl-3,6-dioxo-dihydropyridazin-1(*2H*)-yl)propanamido)ethoxy)ethoxy)ethyl)carbamate **4** (31.0 mg, 50.0 µmol, 52%). ^{1}H NMR (600 MHz, $CDCl_3$, rotamers) δ 7.84 (s, 0.3H), 6.54 (s, 0.5H), 5.77 (s, 0.3H), 5.26 (s, 0.5H), 4.43 (t, *J* = 6.6 Hz, 2H), 4.14–4.12 (m, 2H), 3.72 (s, 3H), 3.60–3.57 (m, 6H), 3.53–3.52 (m, 2H), 3.44–3.43 (m, 2H), 3.39–3.35 (m, 2H), 2.62 (t, *J* = 6.6 Hz, 2H), 2.28–2.22 (m, 6H), 1.58–1.57 (m, 2H), 1.35–1.32, 1.24 (s, 1H), 0.96–0.94 (m, 2H); ^{13}C NMR (150 MHz, $CDCl_3$, rotamers) δ 169.2 (C), 157.0 (C), 153.1 (C), 153.0 (C), 136.4 (C), 135.5 (C), 99.0 (C), 70.6 (CH_2), 70.6 (CH2) 70.3 (CH_2), 69.7 (CH_2), 63.0 (CH_2), 44.6 (CH_2), 40.8 (CH_2), 39.5 (CH_2), 35.2 (CH_3), 34.1 (CH_2), 29.9 (CH), 29.2 (CH_2), 22.8 (CH_2), 21.6 (CH_2), 20.2 (CH_2), 17.9 (CH), 14.3 (CH); IR (thin film) 3243, 2946, 1707, 1618, 1569, 1069, 648 cm^{-1}; LRMS (ESI), 687 (50, [M^{81}Br^{81}Br+Na]$^+$) 685 (100, [M^{79}Br^{81}Br+Na]$^+$), 683 (50, [M^{79}Br^{79}Br+Na]$^+$), 663 (60, [M^{79}Br^{81}Br+H]$^+$); HRMS (ESI) calcd for $C_{25}H_{35}Br_2N_4O_7$ [M^{79}Br^{81}Br+H]$^+$ 663.0847; observed 663.0846.

1.13 Compound **S10,** Di-*tert*-butyl 1-(prop-2-yn-1-yl)hydrazine-1,2-dicarboxylate[6,8]

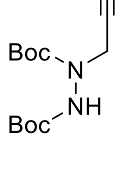

To a solution of di-*tert*-butyl hydrazine-1,2-dicarboxylate (2.00 g, 8.60 mmol) in a mixture of toluene (10 mL) and 5 % aq. NaOH (10 mL) were added tetra-*n*-butylammonium bromide (0.09 g, 0.22 mmol) and

propargyl bromide (3.08 g, 26.00 mmol). The reaction mixture was stirred at 20 °C for 16 h. After this time, H_2O (100 mL) was added, and the mixture was extracted with EtOAc (3 × 50 mL). The combined organic layers were washed with brine (50 mL), dried ($MgSO_4$), and concentrated under vacuum. Purification by silica column chromatography (90% cyclohexane/EtOAc) yielded di-*tert*-butyl 1-(prop-2-yn-1-yl)hydrazine-1,2-dicarboxylate **S10** (1.85 g, 6.87 mmol, 80%) as a white solid: m.p. 101–103 °C (lit. m.p. 103.1–103.4 °C);[8] ^{1}H NMR (600 MHz, $CDCl_3$): δ 6.61 (br. s, 0.75H), 6.14 (br. s, 0.25H), 4.19 (br. s, 2H), 2.20 (t, *J* = 2.44 Hz, 1H), 1.43–1.38 (m, 18H); ^{13}C NMR (125 MHz, $CDCl_3$) δ 154.7 (C), 82.0 (C), 81.6 (C), 78.8 (C), 72.1 (CH), 39.3 (CH_2), 28.3 (CH_3), 28.2 (CH_3); IR (solid): 3311, 2979, 2112, 1709 cm^{-1}.

1.14 Compound **S11,** Tetra-tert-butyl 2,2'-(butane-1,4-diyl)bis(1-(prop-2-yn-1-yl)hydrazine-1,2-dicarboxylate)

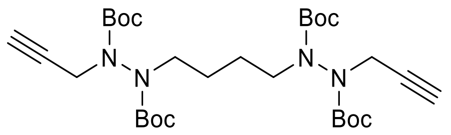

To a solution of di-*tert*-butyl 1-(prop-2-yn-1-yl)hydrazine-1,2-dicarboxylate **S10** (0.40 g, 1.48 mmol) and Cs_2CO_3 (0.97 g, 2.96 mmol) in DMF (10 mL) was added 1,4-dibromobutane (0.11 g, 0.06 mL, 0.49 mmol). The resulting solution was stirred at room temperature for 12 h. After this time, another portion of 1,4-dibromobutane (0.05 g, 0.03 mL, 0.25 mmol) was added and the resulting solution was stirred for another 6 h. After this time, H_2O was added (80 mL) and the mixture was extracted with EtOAc (3 × 60 mL), dried (Na_2SO_4), and concentrated *in vacuo*. Purification by silica column chromatography (95% Cyclohex/EtOAc) afforded tetra-*tert*-butyl 2,2'-(butane-1,4-diyl)bis(1-(prop-2-yn-1-yl)hydrazine-1,2-dicarboxylate) **S11** (0.15 g, 0.25 mmol, 34%) as a colourless oil. TLC: 50% cyclohex/EtOAc, Rf = 0.95; ^{1}H NMR (400 MHz, $CDCl_3$): δ 4.61–3.87 (m, 4H) 3.58–3.32 (m, 4H), 2.23 (br. s, 2H), 1.70–1.63 (m, 4H), 1.38–1.46 (m, 36H); ^{13}C NMR (100 MHz, $CDCl_3$) δ 155.6 (C), 154.6 (C), 81.9 (C), 81.2 (C), 78.6 (C), 78.5 (C), 72.9 (CH), 72.9 (CH), 49.7 (CH_2), 49.7 (CH_2), 39.7 (CH2), 39.6 (CH_2), 28.3 (CH_3), 28.3 (CH_3), 28.2 (CH_3), 27.0 (CH_3), 25.4 (CH_2); IR (thin film): 3400-3250, 2975, 2933, 1704 cm^{-1}; HRMS (ESI) calcd for $C_{30}H_{50}N_4O_8$ $[M+H]^+$ 595.3701; observed 595.3696.

1.15 Compound **S12**, 2,2'-(butane-1,4-diyl)bis(4,5-dibromo-1-(prop-2-yn-1-yl)-1,2-dihydropyridazine-3,6-dione)

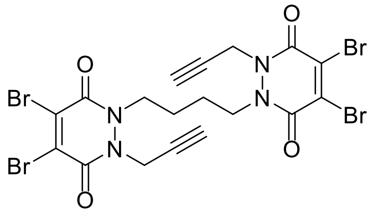

Dibromomaleic acid (0.14 g, 0.53 mmol) was dissolved in AcOH (75 mL) and heated under reflux for 30 min. After this time, tetra-*tert*-butyl 2,2'-(butane-1,4-diyl)bis(1-(prop-2-yn-1-yl)hydrazine-1,2-dicarboxylate) **S11** (0.13 g, 0.21 mmol) was added and the resultant mixture was heated under reflux for a further 12 h (solution turned brown). After this time, the reaction mixture was diluted with H_2O (100 mL) and extracted with EtOAc (3 × 75 mL). The combined organic layers were washed with brine (50 mL), dried (Na_2SO_4), and concentrated *in vacuo* with toluene co-evaporation (as an azeotrope). Purification of the crude residue by silica column chromatography (30% Cyclohex/EtOAc) afforded 2,2'-(butane-1,4-diyl)bis(4,5-dibromo-1-(prop-2-yn-1-yl)-1,2-dihydropyridazine-3,6-dione) **S12** (0.13 mg, 0.20 mmol, 93%) as a yellow viscous oil. ^{1}H NMR (600 MHz, $CDCl_3$): δ 4.88 (d, *J* = 2.4 Hz, 4H), 4.24 (t, *J* = 6.6 Hz, 4H), 2.45 (t, *J* = 2.4 Hz, 2H), 1.80–1.76 (m, 4H); ^{13}C NMR (100 MHz, $CDCl_3$) δ 153.6 (C), 153.3 (C), 135.5 (C), 77.2 (C), 75.5 (CH), 75.2 (CH), 46.9 (CH_2), 37.4 (CH_2), 25.0 (CH_2); IR (thin film): 3255, 2974, 2929, 1709, 1638 cm^{-1}; $C_{18}H_{14}Br_4N_4O_4$ [M^{79}Br^{79}Br^{79}Br^{79}Br+H]$^+$ 666.7821; observed 666.7820

1.16 Compound **9**, N^1,$N^{1'}$-(((((((((butane-1,4-diylbis(4,5-dibromo-3,6-dioxo-3,6-dihydropyridazine-2,1(2*H*)-diyl))bis(methylene))bis(1*H*-1,2,3-triazole-4,1-diyl))bis(ethane-2,1-diyl))bis(oxy))bis(ethane-2,1-diyl))bis(oxy))bis(ethane-2,1-diyl))bis(oxy))bis(ethane-2,1-diyl))bis(N^5-(4-(6-methyl-1,2,4,5-tetrazin-3-yl)benzyl)glutaramide)

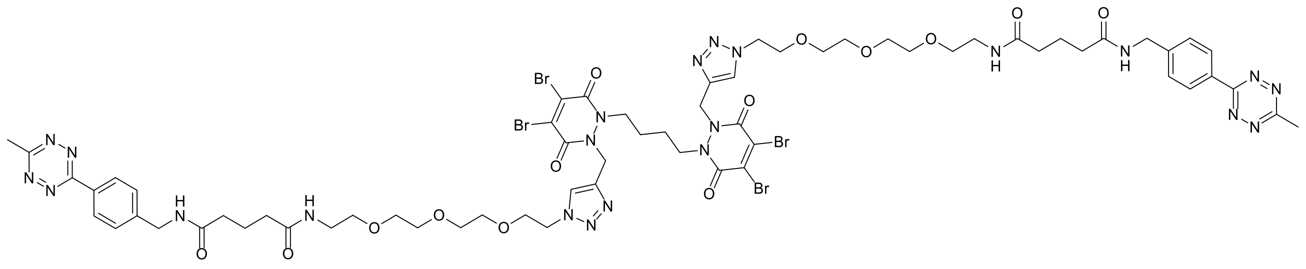

Tetrazine-azide **S5** (50.0 mg, 94.0 µmol) and alkyne **S12** (27.0 mg, 40.5 µmol) were solubilised in DMF (1.5 mL) under an argon atmosphere. $CuSO_4$ (4.1 mg, 16.3 µmol) and THPTA (21.0 mg, 48.9 µmol) were solubilised in 1 mL of PBS (1 M, pH 7.4) under an argon atmosphere (blue solution). The copper solution was added to the azide/alkyne solution followed by argon bubbling. Sodium ascorbate was solubilised in H_2O (500 µL) under argon and added to the azide/alkyne/copper mixture followed by argon bubbling. The

resulting mixture was stirred for 2 h at 37 °C. After this time, the solution was concentrated *in vacuo* and then purified by silica column chromatography (2 to 20 % of MeOH in DCM), to afford bis-tetrazine compound **9** as a pink oil (12.0 mg, 6.9 µmol, 17%).

^{1}H NMR (600 MHz, $CDCl_3$) δ 8.50 (d, *J* = 8.2 Hz, 4H), 7.48 (d, *J* = 8.2 Hz, 4H), 7.89 (s, 2H), 6.68 (t, *J* = 5.9 Hz, 2H), 6.57 (t, *J* = 5.0 Hz, 2H), 5.31 (s, 4H), 4.52–4.48 (m, 8H), 4.45–4.40 (m, 4H), 3.83 (t, *J* = 5 Hz, 4H), 3.58–3.56 (m, 16H), 3.53–3.52 (m, 4H), 3.41–3.39 (m, 4H), 3.08 (s, 6H), 2.33 (t, *J* = 7.1 Hz, 4H), 2.25 (t, *J* = 7.1 Hz, 4H), 2.00 - 1.97 (m, 4H), 1.76–1.73 (m, 4H); ^{13}C NMR (150 MHz, $CDCl_3$) δ 173.0 (C), 172.9 (C), 167.4 (C), 163.9 (C), 153.3 (C), 152.8 (C), 143.7 (C), 140.9 (C), 136.7 (C), 135.4 (C), 130.9 (C), 128.5 (CH), 128.3 (CH), 125.6 (C), 70.6 (CH_2), 70.5 (CH_2), 70.2 (CH_2), 69.8 (CH_2), 69.3 (CH_2), 50.5 (CH_2), 47.4 (CH_2), 43.2 (CH_2), 43.0 (CH_2), 39.3 (CH_2), 35.4 (CH_2), 35.3 (CH_2), 25.0 (CH_2), 22.1 (CH_2), 21.3 (CH_3); IR (thin film): 3500-3250, 2974, 2931, 1708, 1636, 1549 cm^{-1}; HRMS (ESI) calcd for $C_{64}H_{80}Br_4N_{22}O_{14}$ $[M^{79}Br^{79}Br^{79}Br^{79}Br+H]^+$ 1697.3031; observed 1697.3044.

2. Chemical biology section

2.1 General experimental details for chemical biology

Conjugation experiments were carried out in standard 1.5 mL Eppendorf tubes. All buffer solutions were prepared with double-deionised water and filter-sterilized for long-term storage. BBS refers to borate buffered saline (25 mM borate, 25 mM NaCl, pH 8.0, 2 mM EDTA), 5 × BBS refers to borate buffered saline (125 mM borate, 125 mM NaCl, pH 8.0, 10 mM EDTA) and PBS refers to phosphate buffered saline (10 mM phosphate, 2.7 mM KCl, 137 mM NaCl, pH 7.4) unless otherwise stated. Buffer exchange was carried out using sample concentrators (Sartorius Stedim, Vivaspin, MWCO 3, 5 or 10 kDa) or desalting columns (Zeba™ Spin, ThermoFisher Scientific, 7k MWCO). In case where all traces of small molecule had to be removed (for the synthesis of protein–protein conjugates *via* click chemistry) the concentration of small molecule in the sample was diluted to 1 in 10 by Vivaspin, then the remainder removed by Zeba Spin. Rituximab (anti-CD20) and Ontruzant® (tradename of trastuzumab, anti-HER2) antibodies were purchased from UCLH. Anti-CD3 antibody (OKT3) was purchased from BioLegend. For protein A purification Pierce™ Protein A IgG binding Buffer was used. Purification by size exclusion chromatography (SEC) was carried out on an Agilent 1100 HPLC system (column: Superdex 200 increase, 10/300 GL) with a MALS system attached (Optilab T-rEX, Dawn8$^+$ Heleos, Wyatt Technology), equilibrated in PBS pH 7.5 with 0.05% NaN_3 at the ISMB biophysics institute. Detection was by absorption at 280 nm.

2.2 Protein LC-MS

Protein conjugates were prepared for analysis by desalting into distilled water (Zeba™ Spin, ThermoFisher Scientific, 7k MWCO) to achieve approximate concentrations of 4-5 µM (1.0 mg × mL^{-1}) and submitted to

the UCL Chemistry Mass Spectrometry Facility at the Chemistry Department, UCL for analysis on the Agilent 6510 QTOF LC-MS system (Agilent, UK). 10-20 µL of each sample was injected onto a PLRP-S, 1000 Å, 8 µM, 150 mM × 2.1 mM column (Agilent, UK), which was maintained at 60 °C. The separation was achieved using mobile phase A (5% MeCN in 0.1% formic acid) and B (95% MeCN, 5% water 0.1% formic acid) using a gradient elution (Table S1). The column effluent was continuously electrosprayed into the capillary ESI source of the Agilent 6510 QTOF mass spectrometer and ESI mass spectra were acquired in positive electrospray ionisation (ESI) mode using the m/z range 1,000−8,000 in profile mode with Quad AMU set to 500. The raw data was converted to zero charge mass spectra using a maximum entropy deconvolution algorithm, over the appropriate regions as identified *via* the LC trace, with the software, MassHunter (version B.07.00).

Table S1 | **Gradient for LC-MS elution.**

System used: Agilent 6510 QTOF LC-MS (Agilent, UK). Column: PLRP-S, 1000 Å, 8 µM, 150 mM × 2.1 mM (Agilent, UK).

Time (min)	%A (H_2O 0.1% FA)	%B (MeCN 0.1% FA)
0	85	15
2	85	15
3	68	32
4	68	32
14	50	50
18	5	95
20	5	95
22	85	15
25	85	15

2.3 SDS-PAGE gels

Non-reducing glycine-SDS-PAGE with 10%, 12% or 15% acrylamide gels (based on protein size: 10% for 45-150 kDa, 12% for 40-90 kDa and 15% for 25-45 kDa) were performed following standard lab procedures. A 6% stacking gel was used and a broad-range MW marker (10–250 kDa, PageRuler™ Plus Pre-stained Protein Ladder, Thermo Scientific™) was co-run to estimate protein weights. Samples (6-10 µL at ~10 µM protein) were mixed with loading buffer (1-2 µL, composition for 6 × SDS: 1 g SDS, 3 mL glycerol, 6 mL 0.5 M Tris buffer pH 6.8, 2 mg bromophenol blue in 10 mL) and heated at 75 °C for 3 min. 6-8 µL of the samples was loaded into each well. The gels were run at 80 V for 15 min and 160 V for 40-60 min in 1 × SDS running buffer. The gels were stained with Coomassie Blue dye, and destained with distilled water under microwave irradiation.

2.4 UV-Vis spectroscopy

UV-Vis spectroscopy was used to determine the concentrations of protein constructs using a NanoDrop™ One microvolume UV-Vis spectrophotometer operating at RT. Sample buffer was used as blank for baseline correction. Extinction coefficients (Ɛ) for proteins and small molecules (at 280 nm) are listed below (Table S2). Where possible ε_{280} of proteins was determined from amino acid sequence information with tools such as ProtParam (https://web.expasy.org/protparam/). Where necessary, in lieu of sequence information, the generic value of 70000 $M^{-1}cm^{-1}$ was used for Fabs. The ε_{280} of the protein was totalled with that of the expected small molecules adducts to calculate the total Ɛ of the construct at 280 nm ($\Sigma\varepsilon_{280}$). The concentration (c) of the construct was determined by the following equation:

$$c = \frac{A_{280}}{\Sigma\varepsilon_{280}}$$

Table S2 **| Extinction coefficient (Ɛ) values used for proteins and small molecules at 280 nm.**

**Protein**	**ε_{280} ($M^{-1}cm^{-1}$)**	**Small molecule**	**ε_{280} ($M^{-1}cm^{-1}$)**
Fab_{HER2}	71905	Br_2PD-BCN **4**	2275
Fab_{CD20}	82905	Br_2PD-Tz **3**	28340
$Fab_{Generic}$	70000	bis-PD$(Tz)_2$ **9**	56680
Fc_{CD20}	80000		
$mAb_{Generic}$	220000		

2.5 Digestion of antibodies

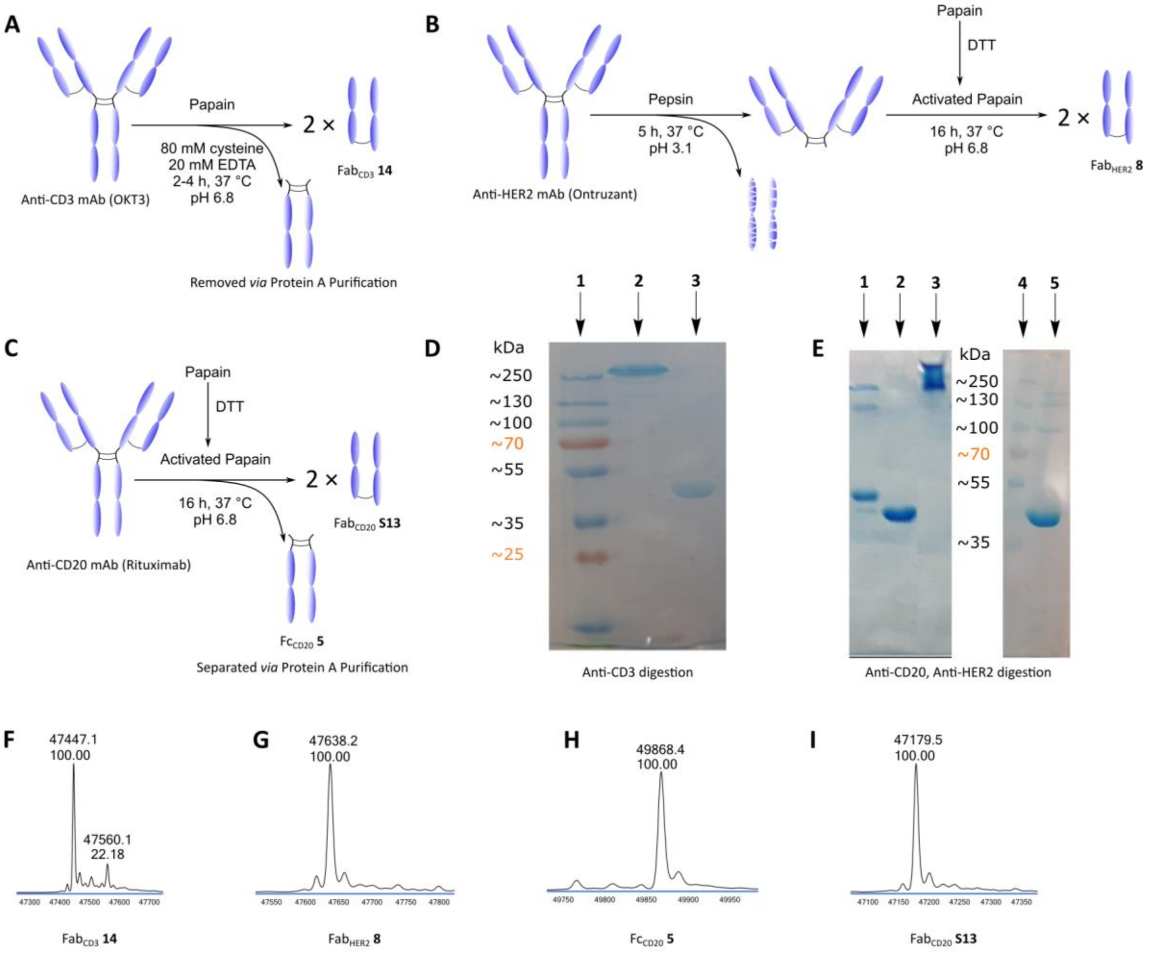

Figure S1 | **Overview of digestion of monoclonal antibodies (mAbs) to their corresponding fragments antigen binding (Fabs).**

A | Protocol used in the case of anti-CD3 mAb (OKT3). Papain, activated with 80 mM cysteine *in situ,* was used to generate Fab and Fc fragments of the parent mAb. These were then separated by protein A purification. **B |** Digestion protocol used in the case of anti-CD20 mAb (Rituximab). Papain, pre-activated with 10 mM DTT, was used to generate Fab and Fc fragments of the parent mAb. These were then separated by protein A purification. **C |** Protocol used for digestion of anti-HER2 mAb (trastuzumab/ Ontruzant®). The Fc fragment was removed by pepsin digestion as the Fab of trastuzumab binds protein A, thus making it impossible to separate the Fab and Fc that way. After the generation of $F(ab')_2$ fragments, pre-activated papain (with DTT) was used to remove the hinge region and furnish clean Fab_{HER2} **8**. **D |** SDS-PAGE of digestion and purification of Fab_{CD3} **14**. Lane 1: Ladder. Lane 2: mAb_{CD3}. Lane 3: Purified Fab_{CD3} **14**. **E |** SDS-PAGE of Fab_{HER2} **8** and Fab_{CD20} **S13** digestion. Lane 1: Fc_{CD20} 5. Lane 2: Fab_{CD20} **S13**. Lane 3: mAb_{CD20} (Rituximab). Lane 4: Ladder. Lane 5: Fab_{HER2} **8**. **F |** LC-MS analysis of Fab_{CD3} **14**. Observed mass: 47449 Da and 47563 Da (Δ = 114 Da, Asn). **G |** LC-MS analysis of Fab_{HER2} **8**. Expected mass: 47638 Da. Observed mass: 47639 Da. **H |** LC-MS analysis of Fab_{CD20} **S13**. Expected mass: 47181 Da. Observed mass: 47180 Da. **I |** LC-MS analysis of Fc_{CD20} **5**. Observed mass: 49868 Da. **| Relevant amino acid residue masses. |**Asparagine: 114.04 Da, Aspartic acid: 115.02 Da.

2.6 Digestion of anti-CD3 mAb (OKT3)

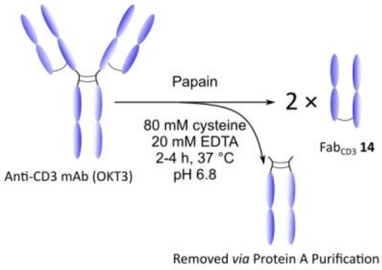

Anti-CD3 antibody (clone: OKT3, 1-20 mg, 10 mg/mL) was buffer exchanged into digest buffer (20 mM NaH_2PO_4, 10 mM EDTA, 80 mM cysteine·HCL, pH 7.0). Immobilized papain (papain/antibody ratio 1:40 w/w) was washed three times with digest buffer and full antibody solution added. The reaction was incubated at 37 °C for 2-4 h under constant agitation (1100 rpm). The resin was separated from the digest using a filter column and washed with Pierce™ Protein A Binding Buffer three times. The digest was combined with the washes and the buffer was exchanged completely for Pierce™ Protein A Binding Buffer and the volume adjusted to 1.5 mL. The Fab and Fc were then separated by protein A purification. Representative yield: 71% Fab$_{CD3}$ **14**.

2.7 Protein A purification

The sample was applied to a NAb protein A column (Thermo Scientific) and incubated at RT with end-over-end mixing for 10 min. The non-bound Fab fraction was eluted four times with Pierce™ Protein A Binding Buffer. The bound Fc fraction was eluted four times with Pierce™ IgG elution buffer or 0.1 M glycine buffer (pH 2.5), which was neutralised with 10% (V/V) of a 1.5 M Tris base, pH 8.8 solution. The Fab and Fc solutions were buffer exchanged into BBS.

2.8 Digestion of anti-HER2 mAb (Ontruzant®/trastuzumab)

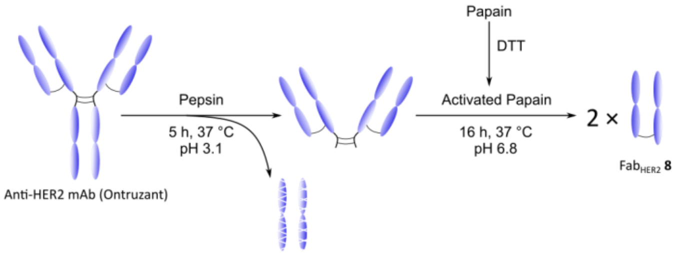

The mAb sample was buffer exchanged into sodium pepsin digest buffer (20 mM NaOAc, pH 3.1). Immobilized pepsin (732 µL) was washed 4 times with pepsin digest buffer and the mAb solution (1 mL, 107 µM) was added to this. The mixture was incubated for 5 h at 37 °C under constant agitation (1100 rpm). The resin was separated from the digest using a filter column and washed 3 times with papain digest buffer (50 mM sodium phosphate, 150 mM NaCl, 1 mM EDTA, pH 6.8). The digest ($F(ab')_2$ solution) was combined with the washes and the volume adjusted to 0.5 mL

Immobilized papain (1.22 mL, 0.25 mg/mL) was activated with 10 mM DTT (in papain digest buffer) with constant agitation (1100 rpm) for 90 min at 37 °C. The resin was washed 4 times with papain digest buffer (without DTT) and the 0.5 mL of F(ab')$_2$ solution was added. The mixture was incubated for 24 h at 37 °C under constant agitation (1100 rpm). The resin was separated from the digest using a filter column, washed 3 times with PBS and the digest combined with the washes. The buffer was exchanged completely for PBS (10 mM phosphate, 2.7 mM KCl, 137 mM NaCl, pH 7.4), and the volume adjusted to 0.5 mL. Representative yield: 56% Fab$_{HER2}$ **8**.

2.9 Digestion of anti-CD20 mAb (Rituximab)

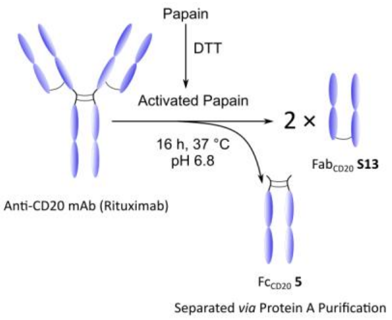

Immobilised papain (0.3 mL, 0.25 mg/mL) was activated with 10 mm DTT in papain digest buffer (50 mM phosphate, 1 mM EDTA, 150 mM NaCl, pH 6.8) under constant agitation (1100 rpm) for 1 h at 25 °C. The resin was washed with papain digest buffer (without DTT) four times and rituximab (3 mg in 0.5 mL of papain digest buffer) was added. The mixture was incubated for 16 h at 37 °C under constant agitation (1100 rpm). Then the resin was separated from the digest using a filter column and washed with Pierce™ Protein A Binding Buffer three times. The digest was combined with the washes and the buffer was exchanged completely for Pierce™ Protein A Binding Buffer and the volume adjusted to 1.5 mL. The Fab and Fc were then separated by protein A purification. Representative yields: 43% Fab$_{CD20}$ **S13**, 31% Fc$_{CD20}$ **5**.

2.10 Reduction of Fab or Fc$_{CD20}$ 5

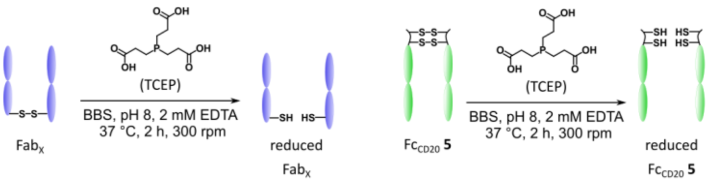

A solution of 20 mM TCEP was prepared by dissolving TCEP-HCl (15 mg) in 5 × BBS (2.6 mL). Fab or Fc$_{CD20}$ **5** (10 µM, 200 µL) was prepared in BBS and 5 × BBS was added (100 µL), followed by addition of 20-60 equivalents of TCEP (20 mM in 5 × BBS, 0.5-6 µL). The mixture was incubated for 120 min at 37 °C under constant agitation (300 rpm). The buffer was then exchanged for BBS to remove excess TCEP (Viva + Zeba spin). NB.: The 5 × BBS was employed to maintain the pH of the solution at 8, as TCEP-HCl is strongly acidic. TCEP-HCl equivalents used for reduction: 60 eq. for Fab$_{CD3}$ **14**, 20 eq. for Fab$_{HER2}$ **8**, and 20 eq. for Fc$_{CD20}$ **5**.

2.11 Re-bridging of Fab and Fc with pyridazinedione

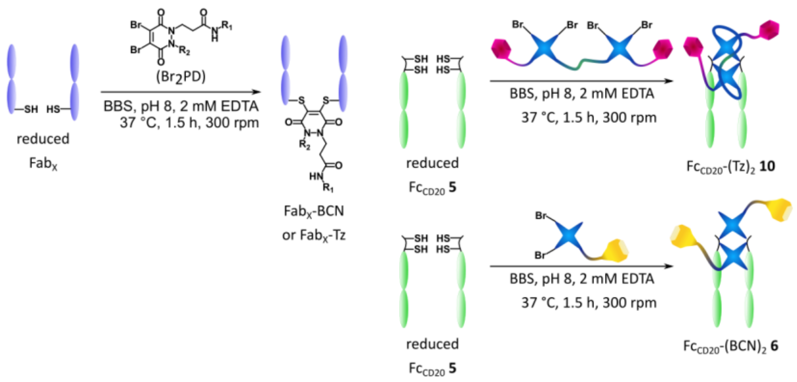

To a solution of reduced Fab_X or Fc_{CD20} **5** (100 µL, 20 µM) in BBS was added 5-20 equivalents of pyridazinedione Br_2PD-Tz **3**, Br_2PD-BCN **4** or bis-PD(Tz)$_2$ **9**. (0.5-2 µL, 20 mM in DMSO) and the mixture incubated at 37 °C with constant agitation (300 RPM) over 120 min. The buffer was then exchanged for BBS to remove excess PD (Viva + Zeba spin). The purity of the sample was assessed by non-reducing SDS-PAGE and high-resolution LC-MS. Representative yields (from the corresponding native Fab or Fc): 76% Fab_{HER2}-BCN **11**, 64% Fc_{CD20}-(BCN)$_2$ **6**, 56% Fab_{HER2}-Tz **7**, 62% Fc_{CD20}-(Tz)$_2$ **10**, and 80% Fab_{CD3}-BCN **15**.

2.12 Preparation of SynAb 1 with the mono-PD method

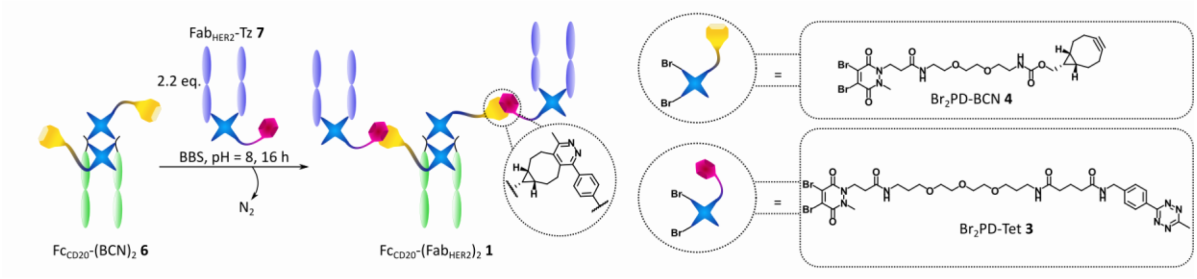

To a solution of Fc_{CD20}-(BCN)$_2$ **6** (105 µL, 60.6 µM, 6.4 nmol, 1 eq.) in BBS was added Fab_{HER2}-Tz **7** (110 µL, 112.8 µM, 12.4 nmol, 1.9 eq.) in BBS. The reaction mixture was concentrated to 80 µL (Viva spin) and incubated at 37 °C for 16 h under an Argon atmosphere. After this time, the mixture was purified by SEC to remove any Fc_{CD20}-(Fab_{HER2})-BCN **S14**. Representative yield: 15% Fc_{CD20}-(Fab_{HER2})$_2$ **1** (from Fc_{CD20}).

2.13 Preparation of SynAb 2 with the bis-PD method

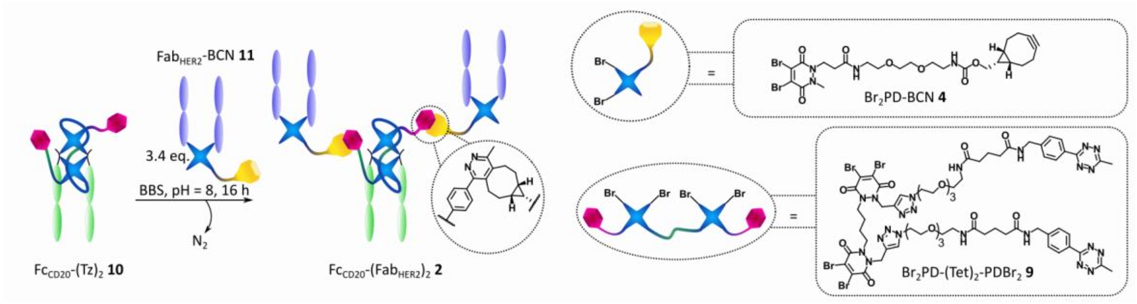

To a solution of Fc_{CD20}-(Tz)$_2$ **10** (117.8 µL, 47.6 µM, 6.2 nmol, 1 eq.) in BBS was added Fab_{HER2}-BCN **11** (116.8 µL, 177.5 µM, 21.3 nmol, 3.4 eq.) in BBS. The reaction mixture was concentrated to 80 µL (Viva

spin) and incubated at 37 °C for 16 h under an Argon atmosphere. After this time, the mixture was purified by SEC to remove any Fc_{CD20}-(Tz)-Fab_{HER2} **12**. Representative yield: 11% Fc_{CD20}-(Fab_{HER2})$_2$ **2** (from Fc_{CD20}).

2.14 Preparation of bispecific SynAb **13** with the bis-PD method

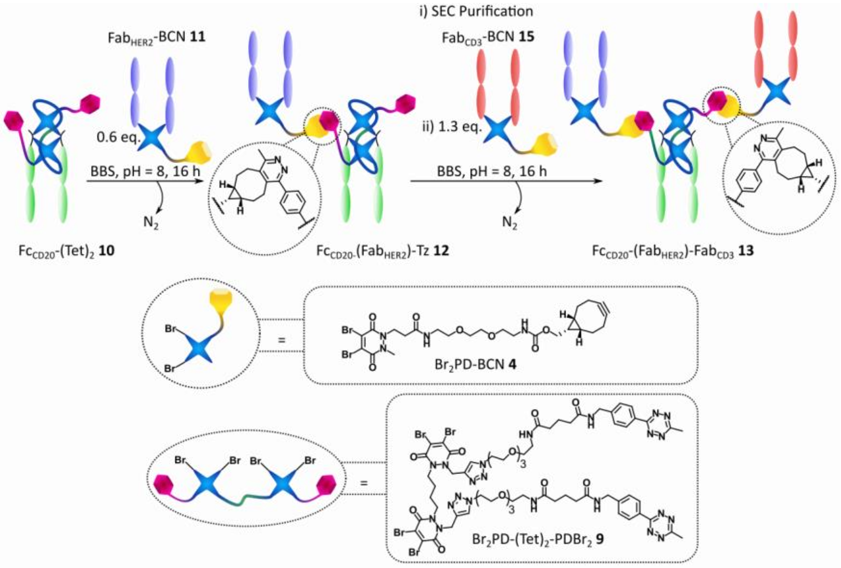

To a solution of Fc_{CD20}-(Tz)$_2$ **10** (120 µL, 430 µM, 51 nmol, 1 eq.) in H_2O was added Fab_{HER2}-BCN **11** (120 µL, 254 µM, 30 nmol, 0.6 eq.) in H_2O. The reaction mixture was concentrated to 80 µL (Viva spin) and incubated at 37 °C for 16 h under an Argon atmosphere. After this time, the mixture was purified by SEC to remove any Fc_{CD20}-(Fab_{HER2})$_2$ **2**. To the resulting Fc_{CD20}-(Fab_{HER2})-Tz **12** solution (50 µL, 180 µM, 9 nmol, 1.0 equiv.) was added Fab_{CD3}-BCN 15 (120 µL, 100 µM, 12 nmol, 1.3 equiv.) in H_2O. The reaction mixture was concentrated to 80 µL (Viva spin) and incubated at 37 °C for 16 h under an Argon atmosphere. After this time, the mixture was purified by SEC to afford Fc_{CD20}-(Fab_{HER2})-Fab_{CD3} bispecific SynAb **13** (6 nmol). Representative yield: 12 % Fc_{CD20}-(Fab_{HER2})-Fab_{CD3} **13** (from Fab_{HER2}).

3. Biological evaluation

3.1 Cell culture

Jurkat cells were cultured in RPMI 1640 supplemented with 10% v/v fetal bovine serum (FBS), 50 units/mL penicillin, 50 µg/mL streptomycin, 1 mM sodium pyruvate and 10 mM HEPES. HCC1954 cells were cultured in RPMI 1640 supplemented with 10% v/v FBS, 50 units/mL penicillin and 50 µg/mL streptomycin. Primary human T cells were isolated from the buffy coat fractions of anonymised healthy blood donors after obtaining ethical approval from the Queen's University Belfast Research Ethics Committee/Northern Ireland Blood Transfusion Service. Purified T cells were cultured in RPMI 1640 supplemented with 10% v/v FBS, 50 units/mL penicillin and 50 µg/mL streptomycin. All cells were maintained in 5% CO_2 at 37°C in a humidified incubator.

3.2 Flow cytometry

A total of 5 x 10^5 HCC1954 cells or Jurkat cells were resuspended in 200 µL FACS buffer (5% v/v FBS in PBS) containing 2.5 µg/mL SynAb **2** or bispecific SynAb **13** and incubated for 45 min at 4°C. Cells were then washed by centrifugation at 300 *x g* for 5 min at 4°C and resuspended in 200 µL FACS buffer containing 2.5 µg/mL PE-conjugated goat anti-human IgG Fc antibody (Thermo Fisher Scientific). Following incubation for 30 min at 4°C in darkness, cells were washed twice in FACS buffer by centrifugation at 300 x g for 5 min at 4°C and PE fluorescence was assessed on a BD Accuri C6 Plus flow cytometer.

3.3 T cell purification

Healthy donor buffy coats were mixed with an equal volume of Hank's Balanced Salt Solution (HBSS) and 30 mL aliquots of diluted blood were gently layered over 15 mL Lymphoprep™ density gradient medium in 50 mL conical tubes, followed by centrifugation at 800 *x g* for 30 min at room temperature without brake. The peripheral blood mononuclear cell (PBMC) fraction was transferred to a fresh tube, resuspended in 50 mL HBSS and centrifuged at 300 *x g* for 10 min at room temperature. To facilitate platelet removal, this HBSS resuspension-centrifugation step was repeated twice at a lower speed of 200 *x g*. T cells were then purified from the PBMC fraction by negative selection using the EasySep™ Human T Cell Isolation Kit (STEMCELL Technologies) in accordance with the manufacturer's instructions.

3.4 T cell/HCC1954 cell coculture

HCC1954 cells were seeded at 5 x 10^3 per well in 96-well plates and left to adhere overnight. Purified T cells were then added to appropriate wells at an effector:target (E:T) ratio of 10:1 +/- SynAb **2** or bispecific SynAb **13** at concentrations ranging from 0.005–500 ng/mL. After 48 h, culture supernatants were

collected and centrifuged to remove cells. Cell-free supernatants were stored at -80°C in preparation for downstream ELISA analysis. Microplate wells were then washed x 2 in PBS and viability of remaining adherent HCC1954 cells was assessed by CellTiter-Glo® assay in accordance with the manufacturer's instructions.

3.5 ELISA

Cell-free culture supernatants were assayed using the human IFN-γ DuoSet ELISA (R&D Systems) in accordance with the manufacturer's instructions.

3.6 Data analysis

FlowJo software (version 10.8.1) was used to construct histograms in Figure 4/A. GraphPad Prism software (version 9.3.1) was used to graph data in Figure 4/B-D. R software (using base functions and the car, dplyr and agricolae packages) was used to perform statistical analysis in Figure 4/B. Statistical significance was determined by two-way analysis of variance (ANOVA). The interaction between cell culture and antibody was also considered. F-ratios, degrees of freedom (df) for each variable and for the residual/error, and p-values were reported. Tukey's HSD (honestly significant difference) test was used for multiple comparisons. Homoscedasticity and normality were assessed both visually and using statistical tests: Levene's and Shapiro-Wilks tests respectively. Simple main effects analysis showed that both cell culture ($F = 853.3$, $df = 1, 48$, $p < 2e-16$) and antibody selection ($F = 1188.8$ $df = 2, 48$, $p < 2e-16$) had a statistically significant effect on cell viability. There was also a statistically significant interaction between the effects of cell culture and antibody ($F = 1086.9$, $df = 2, 48$, $p < 2e-16$). Significance was defined as follows: ****P<0.0001.

4. References

(1) Sarris, A. J. C.; Hansen, T.; de Geus, M. A. R.; Maurits, E.; Doelman, W.; Overkleeft, H. S.; Codée, J. D. C.; Filippov, D. V.; van Kasteren, S. I. Fast and PH-Independent Elimination of Trans-Cyclooctene by Using Aminoethyl-Functionalized Tetrazines. *Chem. - A Eur. J.* **2018**, *24* (68), 18075–18081.

(2) Bahou, C.; Szijj, P. A.; Spears, R. J.; Wall, A.; Javaid, F.; Sattikar, A.; Love, E. A.; Baker, J. R.; Chudasama, V. A Plug-and-Play Platform for the Formation of Trifunctional Cysteine Bioconjugates That Also Offers Control over Thiol Cleavability. *Bioconjugate Chem.* **2021**, *32*, 672–679.

(3) Lang, K.; Davis, L.; Wallace, S.; Mahesh, M.; Cox, D. J.; Blackman, M. L.; Fox, J. M.; Chin, J. W. Genetic Encoding of Bicyclononynes and Trans-Cyclooctenes for Site-Specific Protein Labeling in Vitro and in Live Mammalian Cells via Rapid Fluorogenic Diels-Alder Reactions. *J. Am. Chem. Soc.* **2012**, *134* (25), 10317–10320.

(4) Agramunt, J.; Ginesi, R.; Pedroso, E.; Grandas, A. Inverse Electron-Demand Diels-Alder Bioconjugation Reactions Using 7-Oxanorbornenes as Dienophiles. *J. Org. Chem.* **2020**, *85* (10), 6593–6604.

(5) Hernández-Gil, J.; Braga, M.; Harriss, B. I.; Carroll, L. S.; Leow, C. H.; Tang, M. X.; Aboagye, E. O.; Long, N. J. Development of 68Ga-Labelled Ultrasound Microbubbles for Whole-Body PET Imaging. *Chem. Sci.* **2019**, *10* (21), 5603–5615.

(6) Maruani, A.; Szijj, P. A.; Bahou, C.; Nogueira, J. C. F.; Caddick, S.; Baker, J. R.; Chudasama, V. A Plug-and-Play Approach for the de Novo Generation of Dually Functionalized Bispecifics. *Bioconjugate Chem.* **2020**, *31* (3), 520–529.

(7) Bahou, C.; Richards, D. A.; Maruani, A.; Love, E. A.; Javaid, F.; Caddick, S.; Baker, J. R.; Chudasama, V. Highly Homogeneous Antibody Modification through Optimisation of the Synthesis and Conjugation of Functionalised Dibromopyridazinediones. *Org. Biomol. Chem.* **2018**, *16* (8), 1359–1366.

(8) Rasmussen, L. K. Facile Synthesis of Mono-, Di-, and Trisubstituted Alpha-Unbranched Hydrazines. *J. Org. Chem.* **2006**, *71* (9), 3627–3629.

5. NMR spectra

5.1 Compound **S1**, *tert*-butyl (4-cyanobenzyl)carbamate[3]

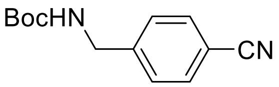

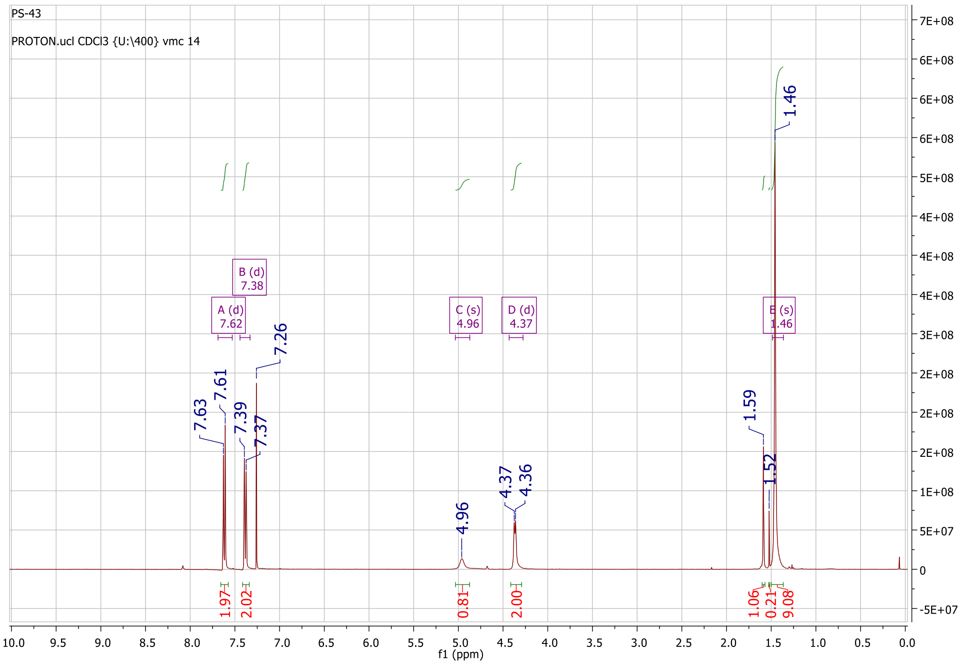

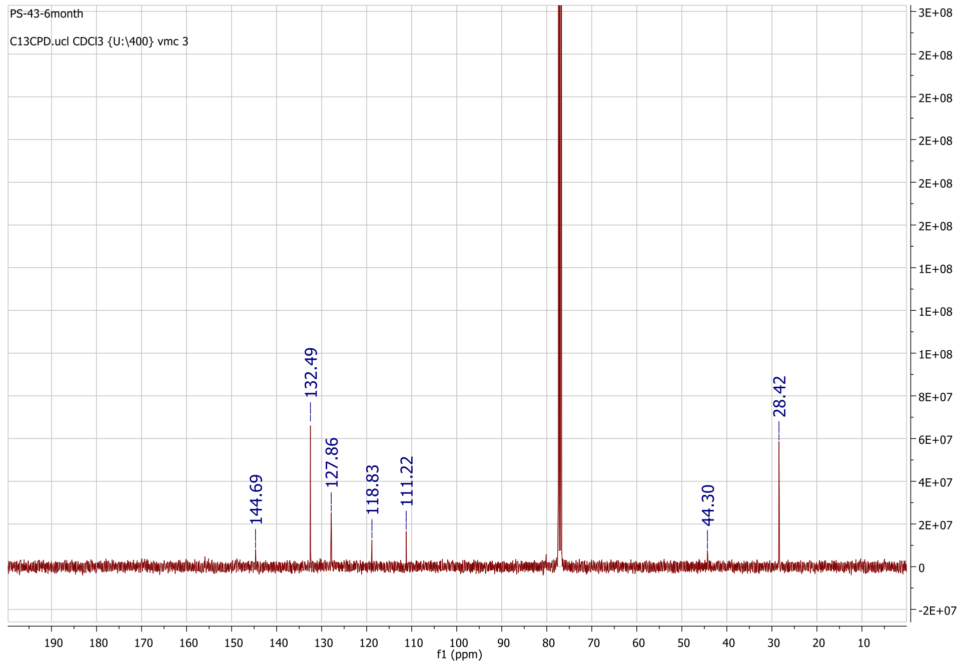

5.2 Compound **S2**, *tert*-butyl (4-(6-methyl-1,2,4,5-tetrazin-3-yl)benzyl)carbamate[3]

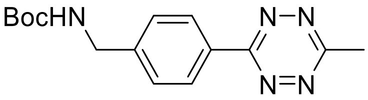

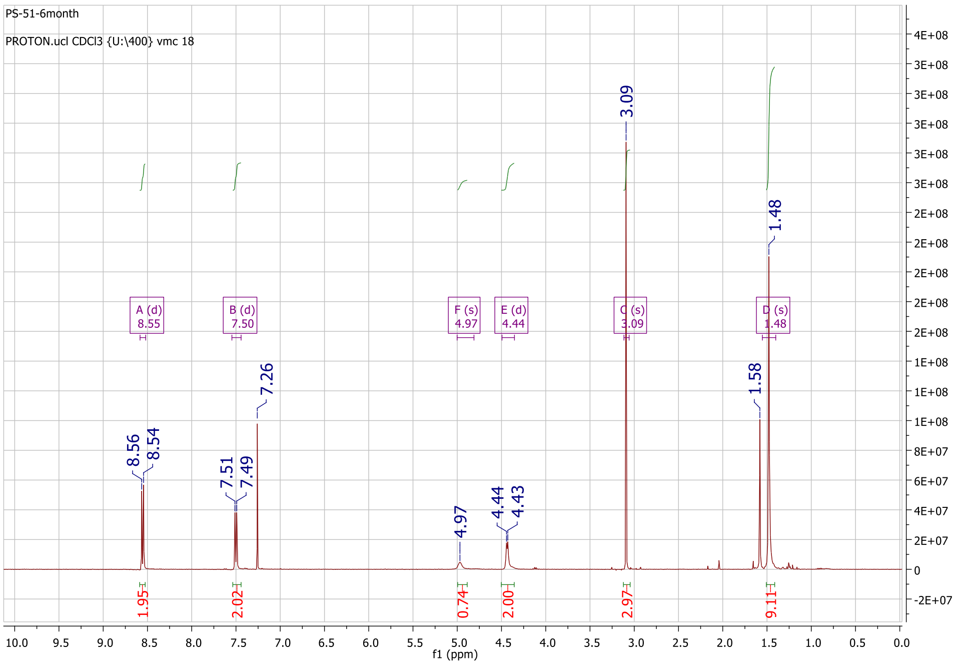

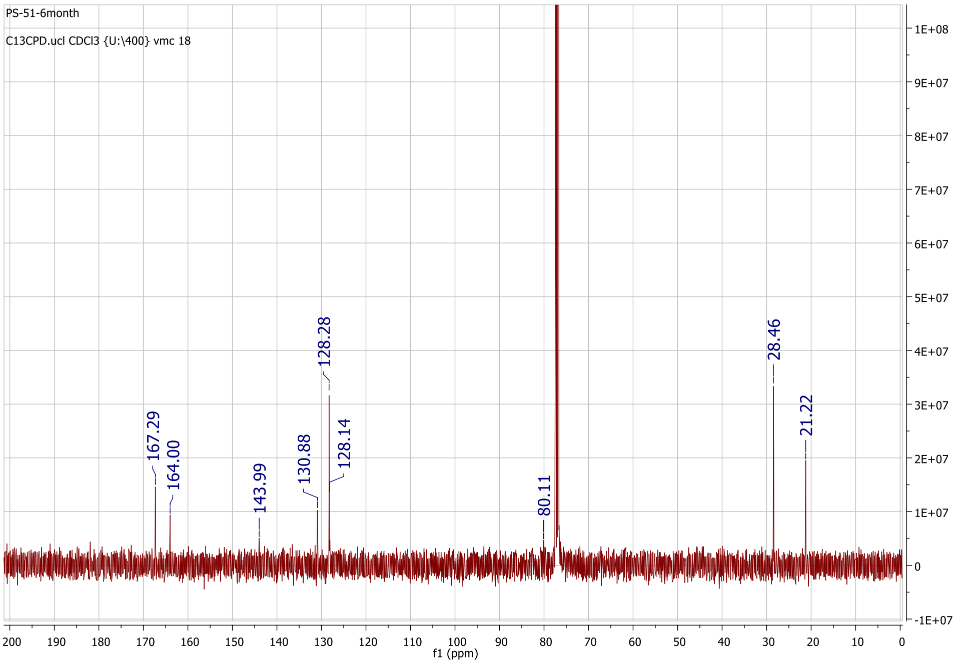

5.3 Compound **S3**, 5-((4-(6-methyl-1,2,4,5-tetrazin-3-yl)benzyl)amino)-5-oxopentanoic acid[5]

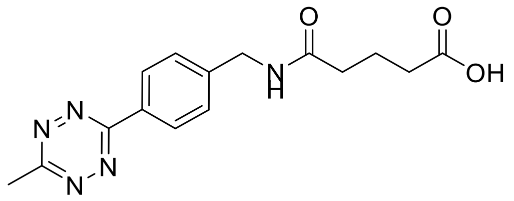

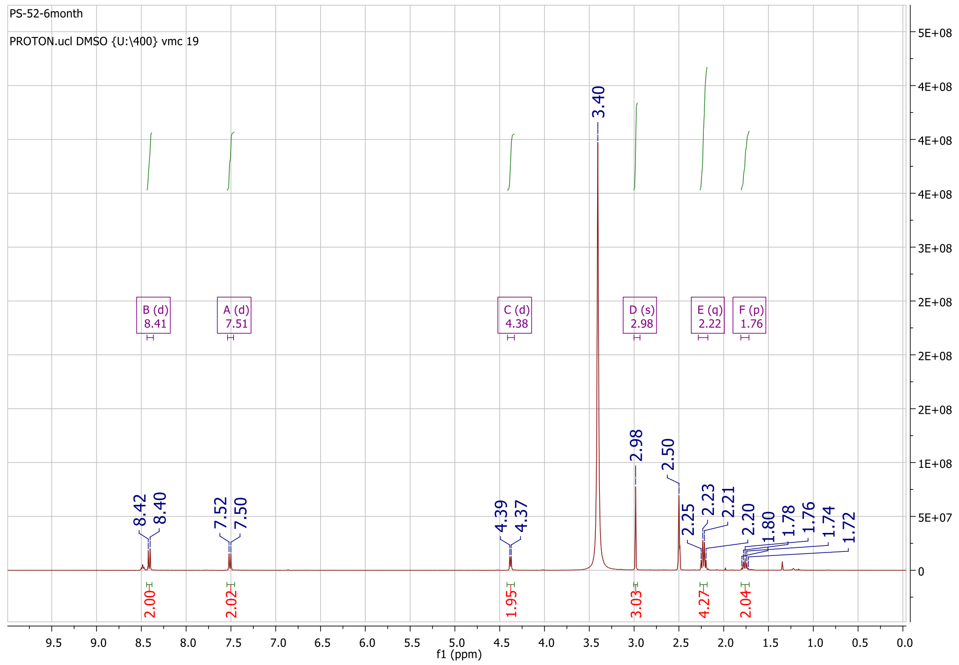

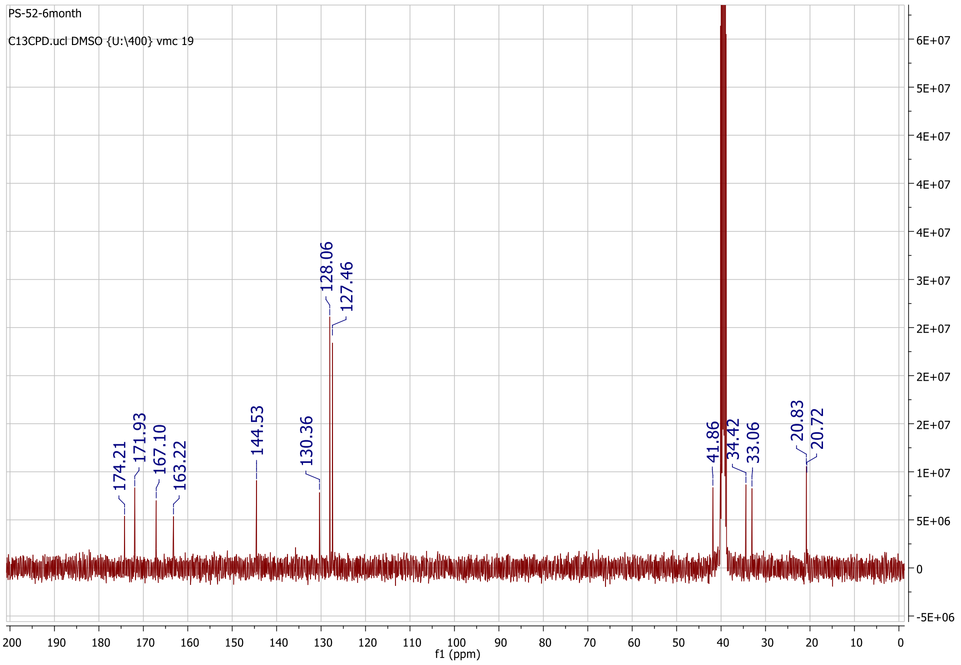

5.4 Compound **S4**, *N*[1]-(3-(2-(2-(3-aminopropoxy)ethoxy)ethoxy)propyl)-*N*[5]-(4-(6-methyl-1,2,4,5-tetrazin-3-yl)benzyl)glutaramide[6]

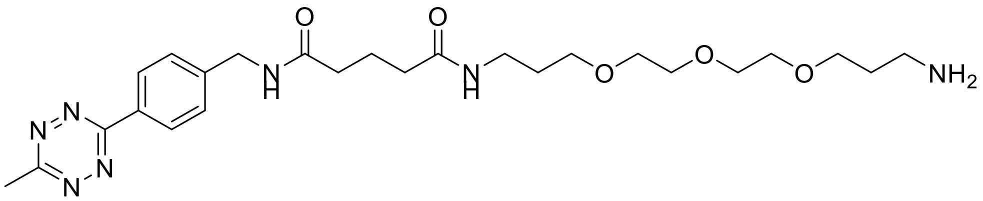

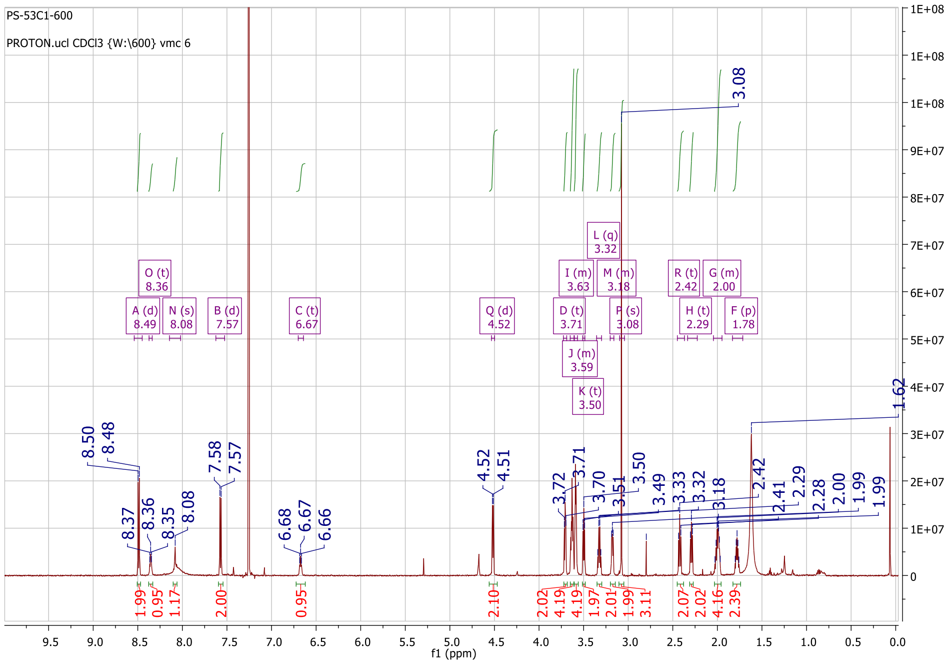

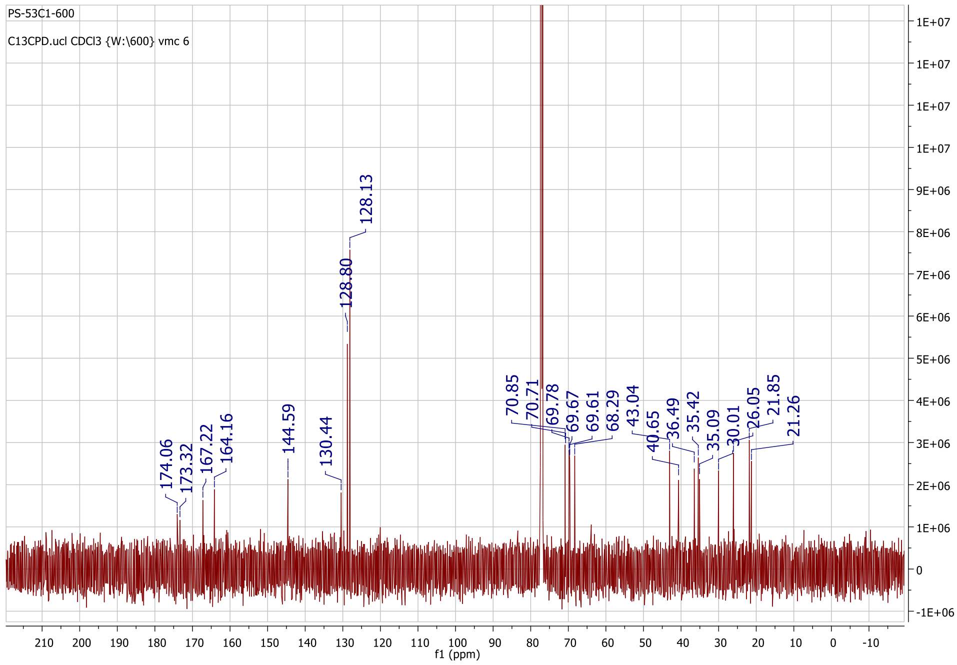

5.5 Compound **S5**, *N*[1]-(2-(2-(2-(2-azidoethoxy)ethoxy)ethoxy)ethyl)-*N*[5]-(4-(6-methyl-1,2,4,5-tetrazin-3-yl)benzyl)glutaramide[2]

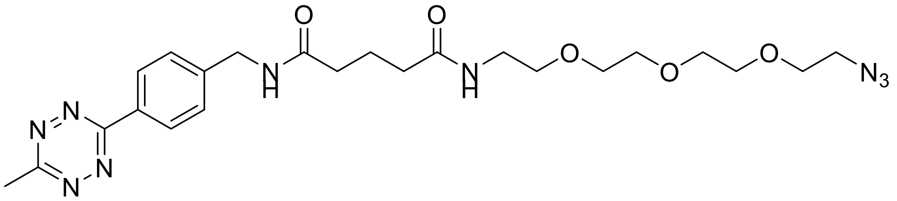

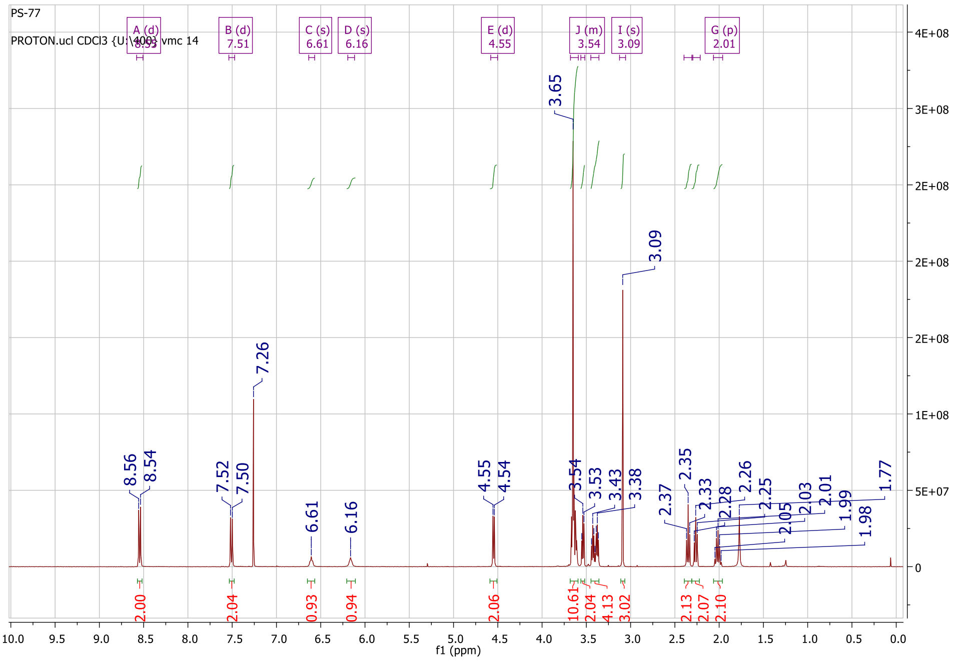

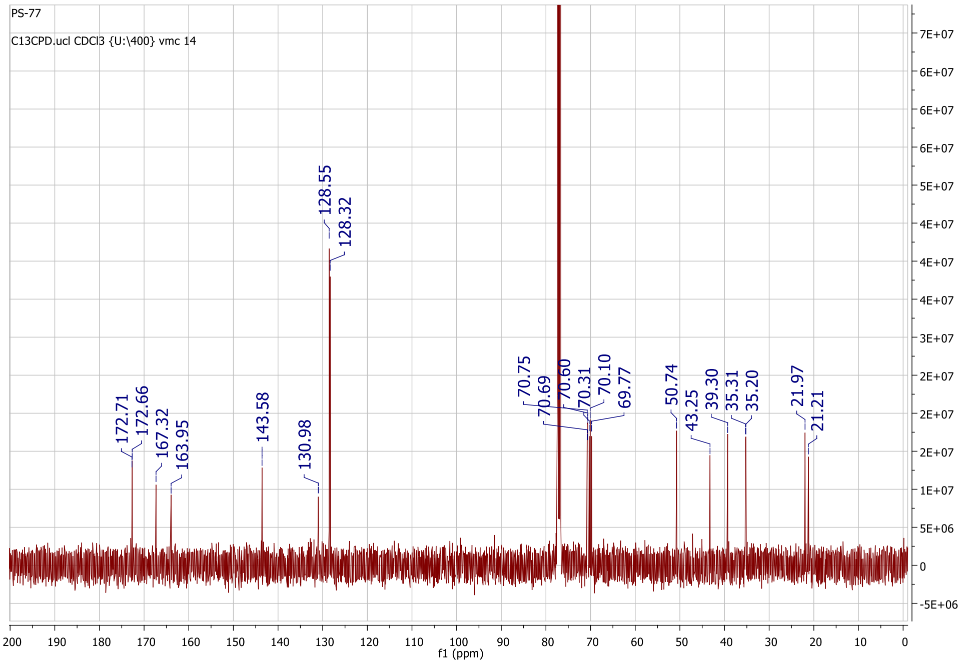

5.6 Compound **S6**, di-*tert*-butyl 1-methylhydrazine-1,2-dicarboxylate[7]

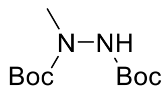

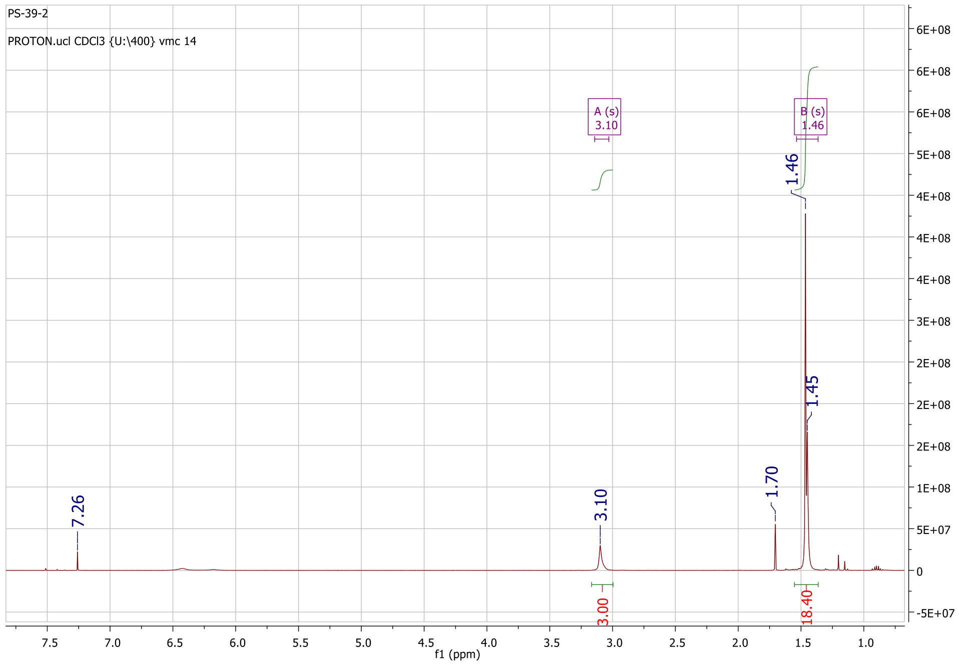

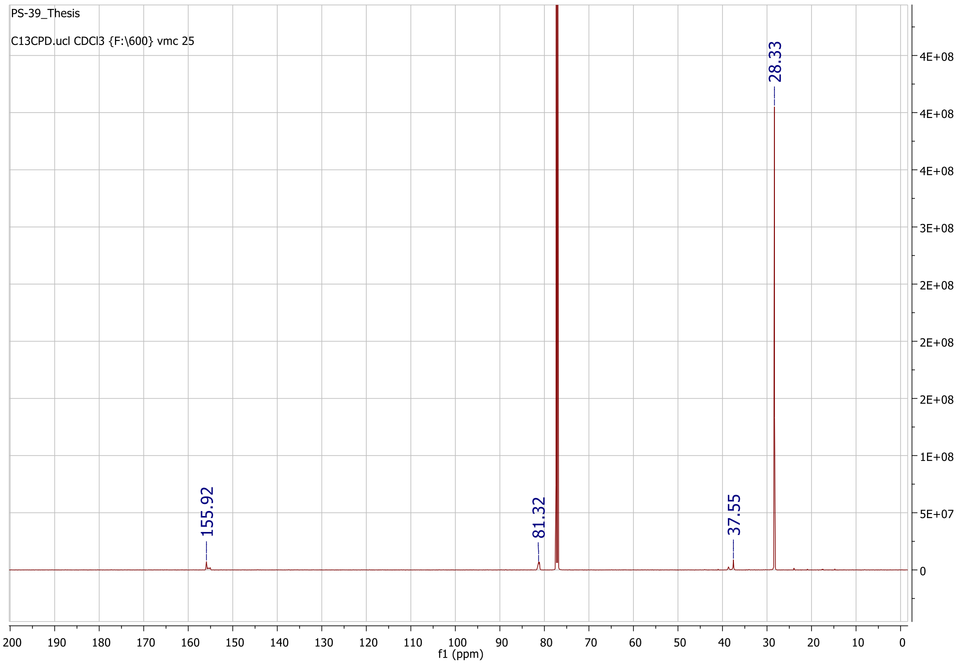

5.7 Compound **S7**, di-*tert*-butyl 1-(3-(tert-butoxy)-3-oxopropyl)-2-methylhydrazine-1,2-dicarboxylate[7]

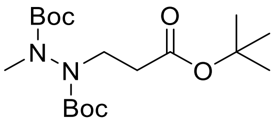

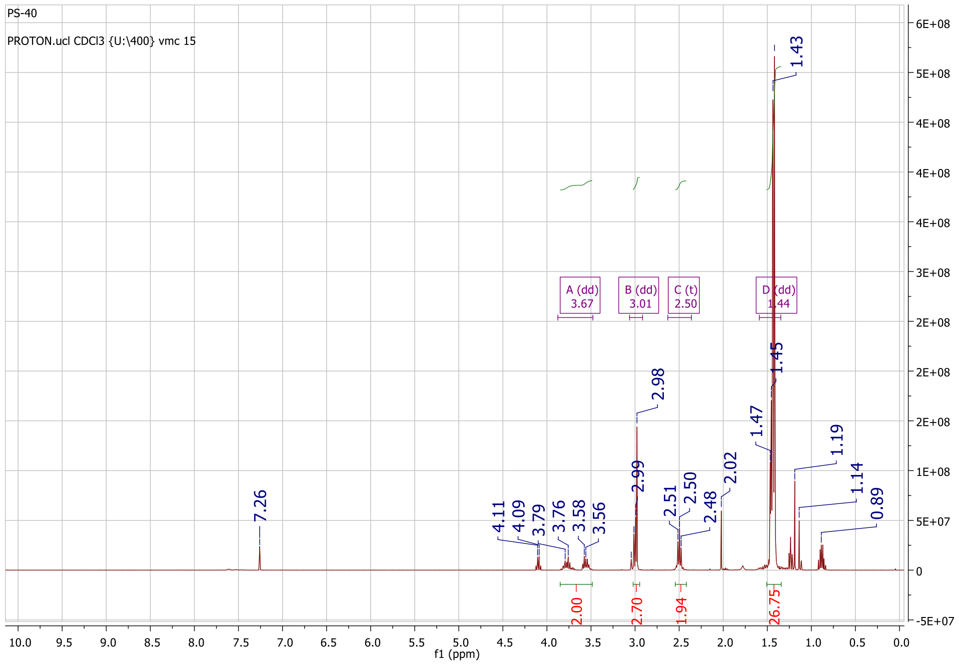

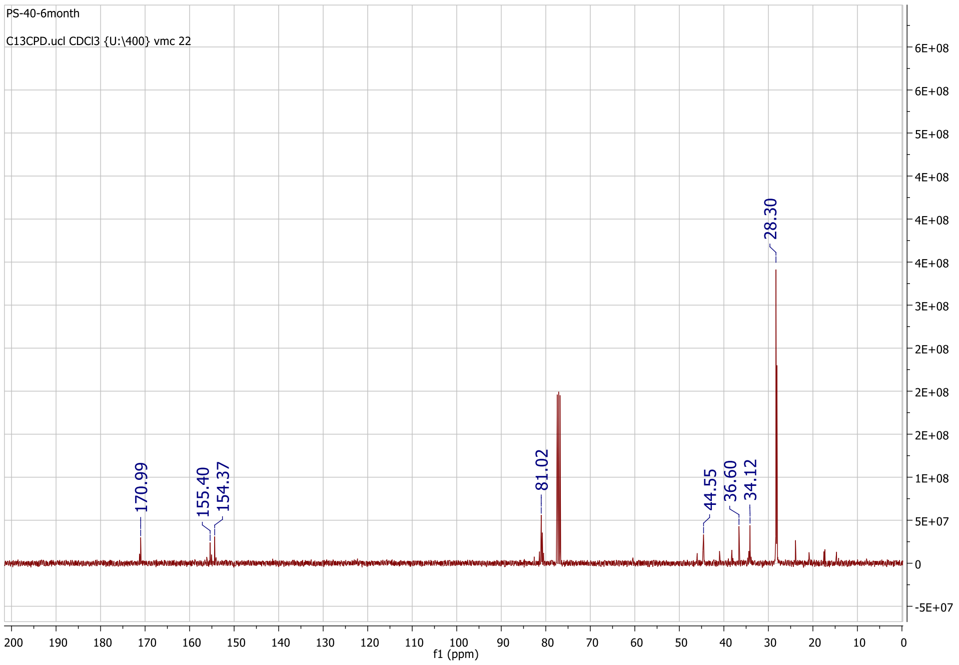

5.8 Compound **S8**, 3-(4,5-dibromo-2-methyl-3,6-dioxo-3,6-dihydropyridazin-1*(2H)-yl*)propanoic acid[7]

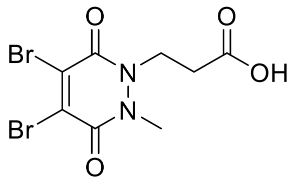

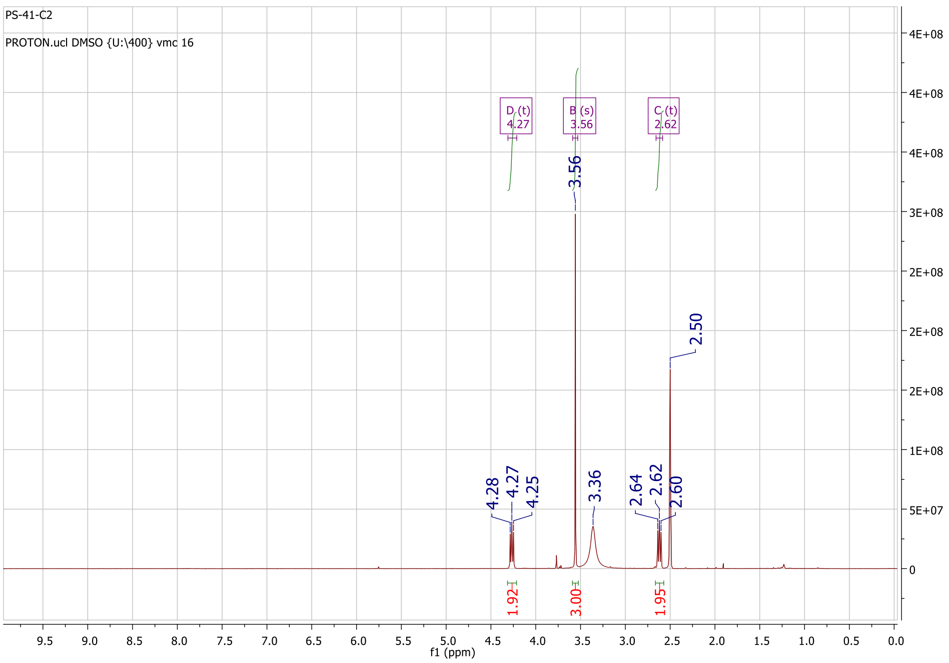

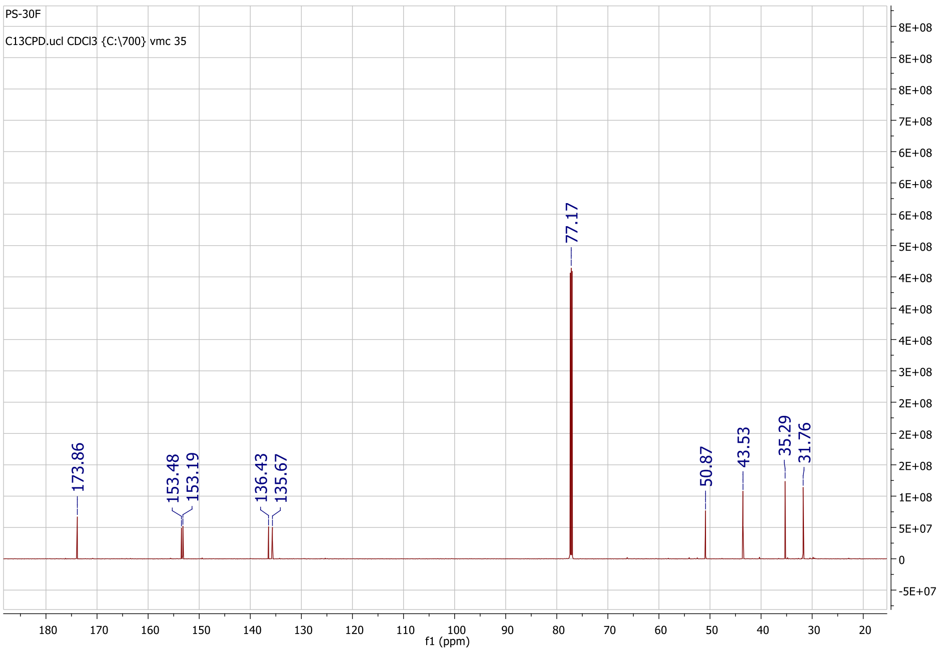

5.9 Compound **S9**, 2,5-dioxopyrrolidin-1-yl 3-(4,5-dibromo-2-methyl-3,6-dioxo-3,6-dihydropyridazin-1*(2H)-yl*)propanoate[7]

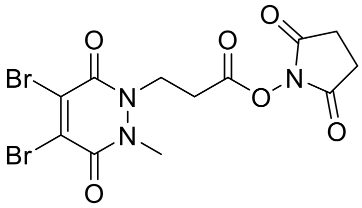

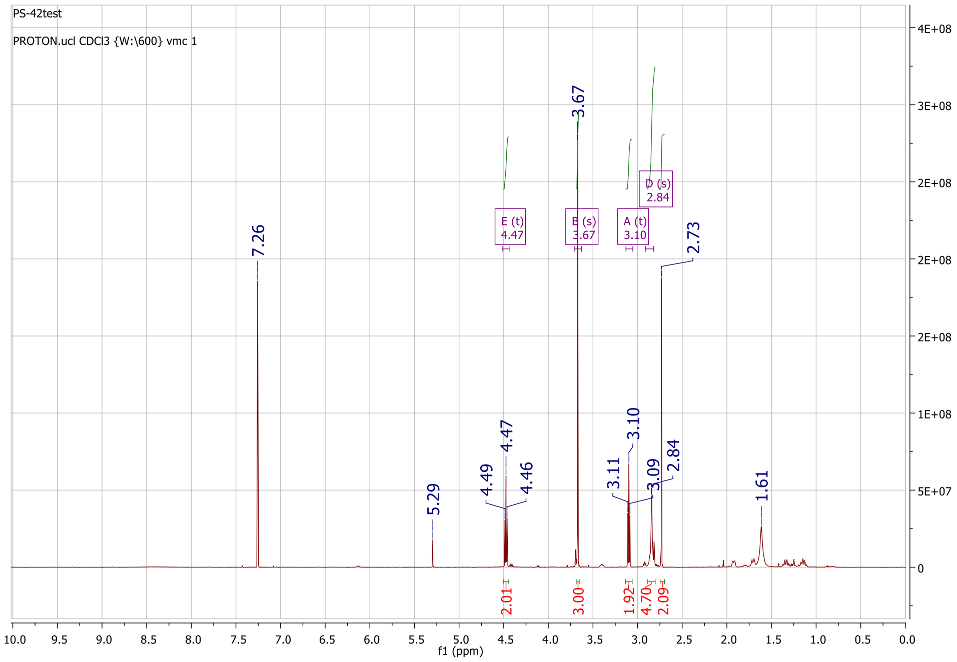

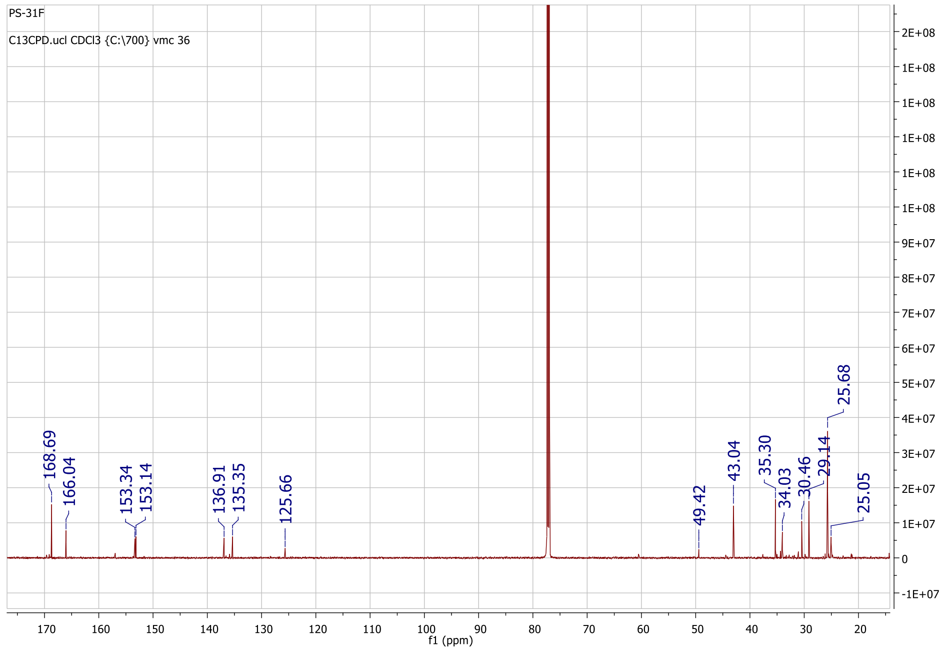

5.10 Compound **3**, N^1-(17-(4,5-dibromo-2-methyl-3,6-dioxo-3,6-dihydropyridazin-1*(2H)-yl*)-15-oxo-4,7,10-trioxa-14-azaheptadecyl)-N^5-(4-(6-methyl-1,2,4,5-tetrazin-3-yl)benzyl)glutaramide[6]

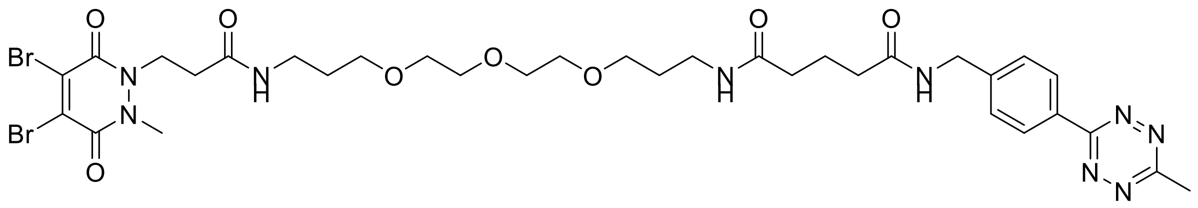

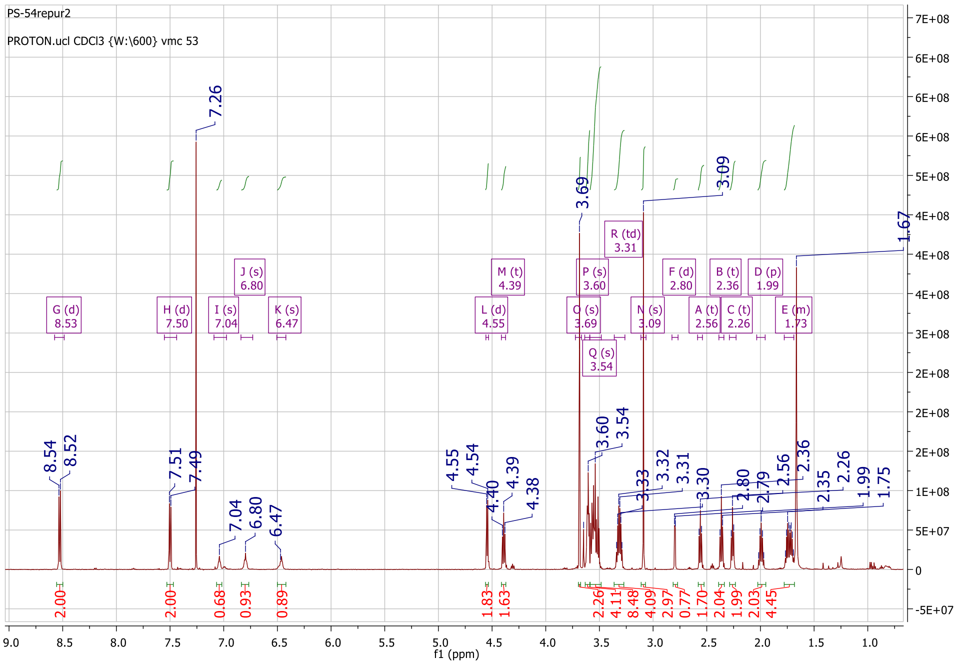

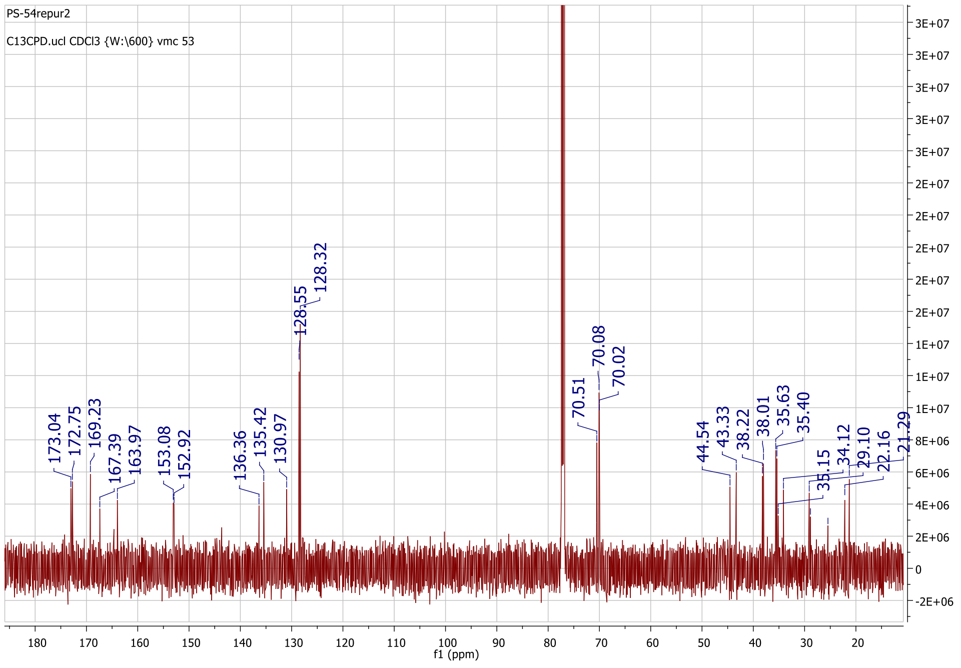

5.11 Compound **4**, ((1R,8S,9s)-bicyclo[6.1.0]non-4-yn-9-yl)methyl (2-(2-(2-(3-(4,5-dibromo-2-methyl-3,6-dioxo-3,6-dihydropyridazin-1*(2H)*-*yl*)propanamido)ethoxy)ethoxy)ethyl)carbamate[7]

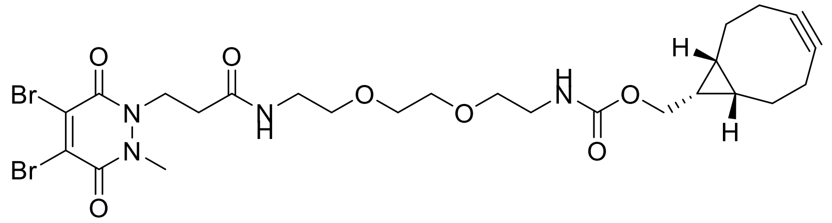

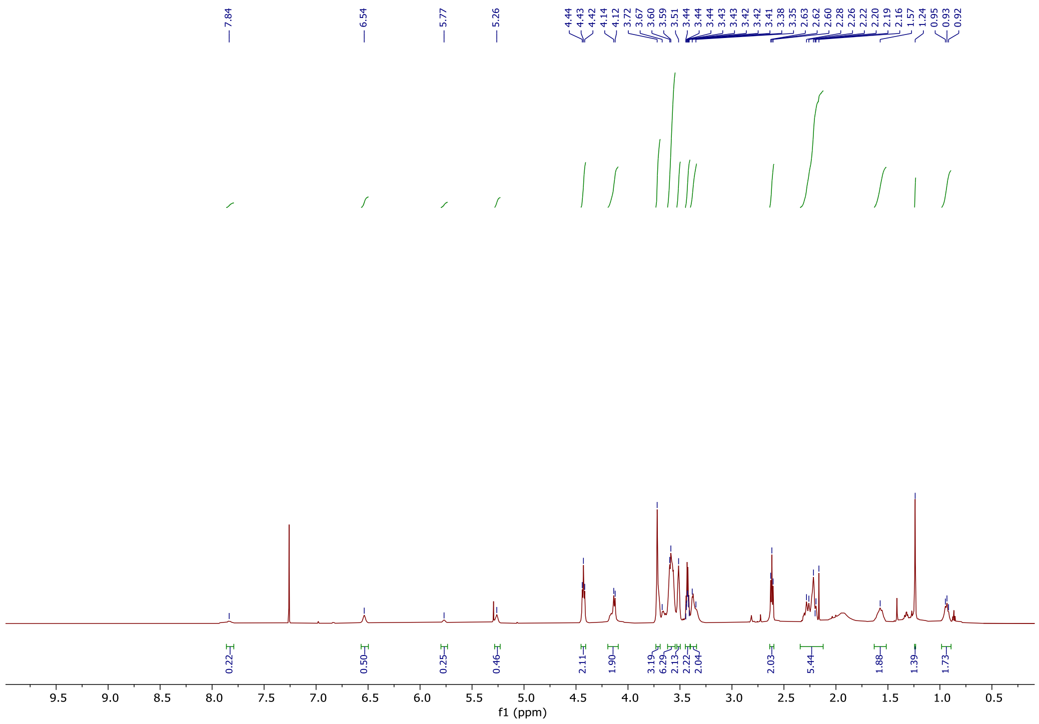

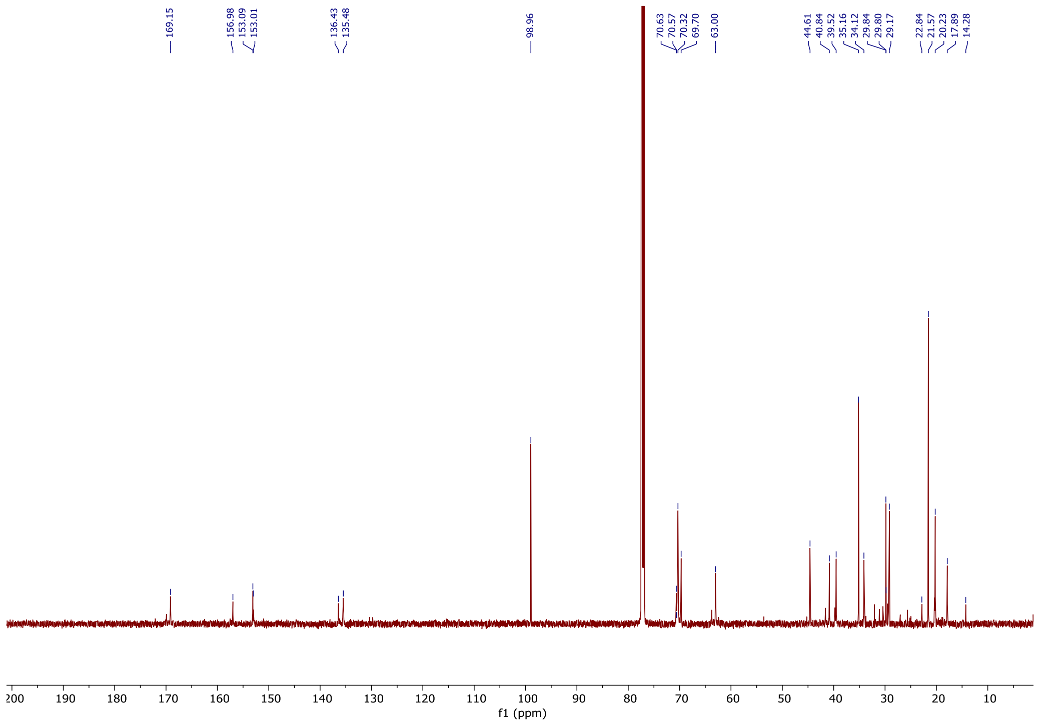

5.12 Compound **S10**, di-*tert*-butyl 1-(prop-2-yn-1-yl)hydrazine-1,2-dicarboxylate[6,8]

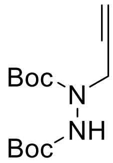

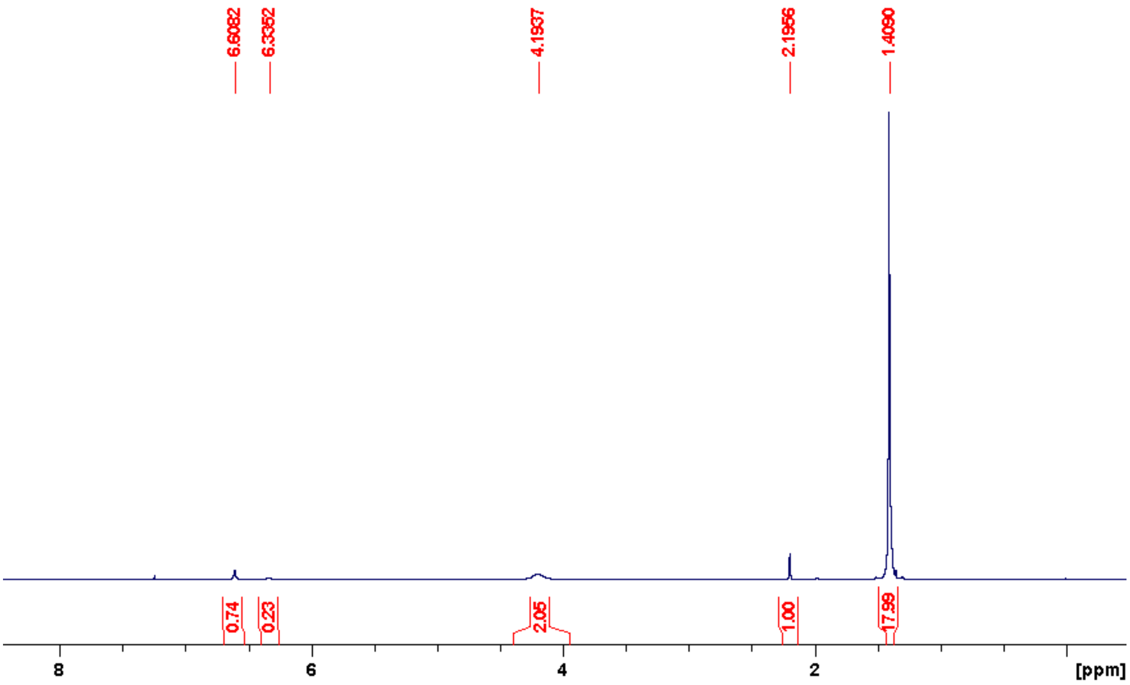

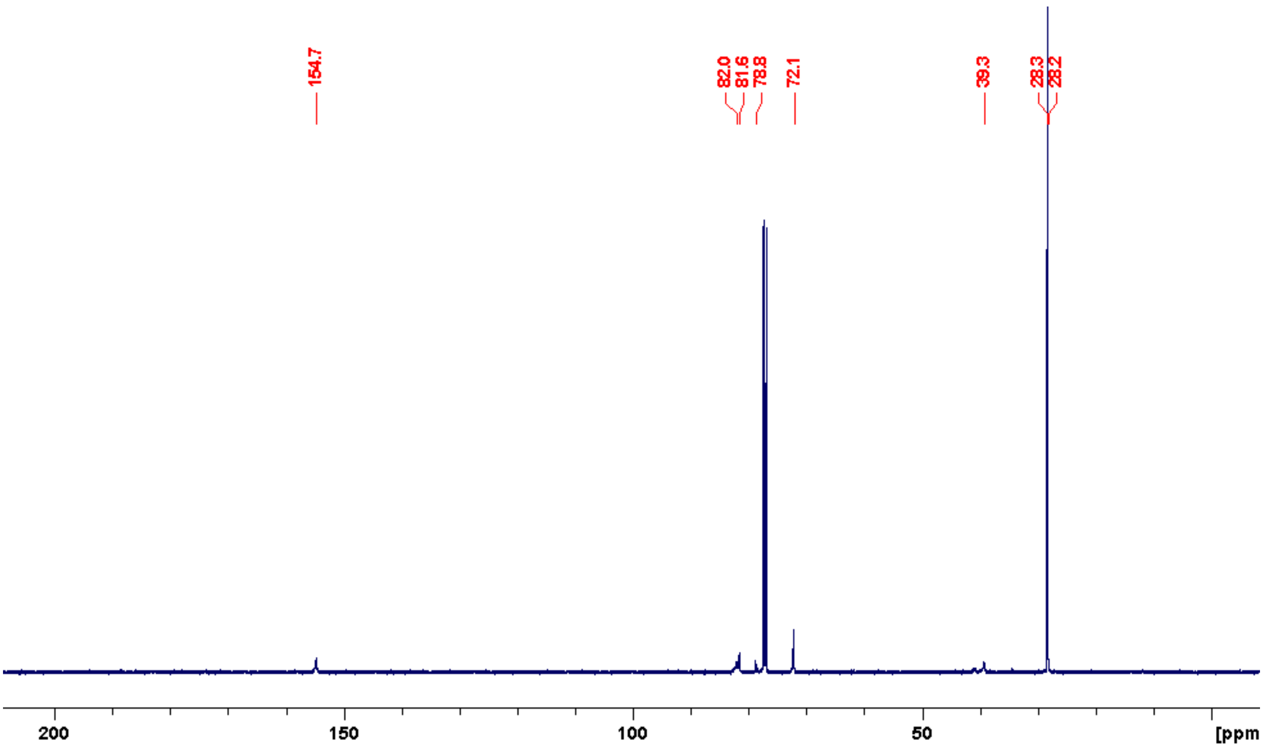

5.13 Compound **S11**, Tetra-*tert*-butyl 2,2'-(butane-1,4-diyl)bis(1-(prop-2-yn-1-yl)hydrazine-1,2-dicarboxylate)

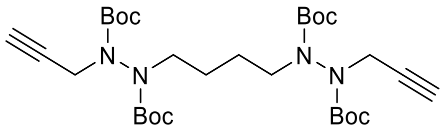

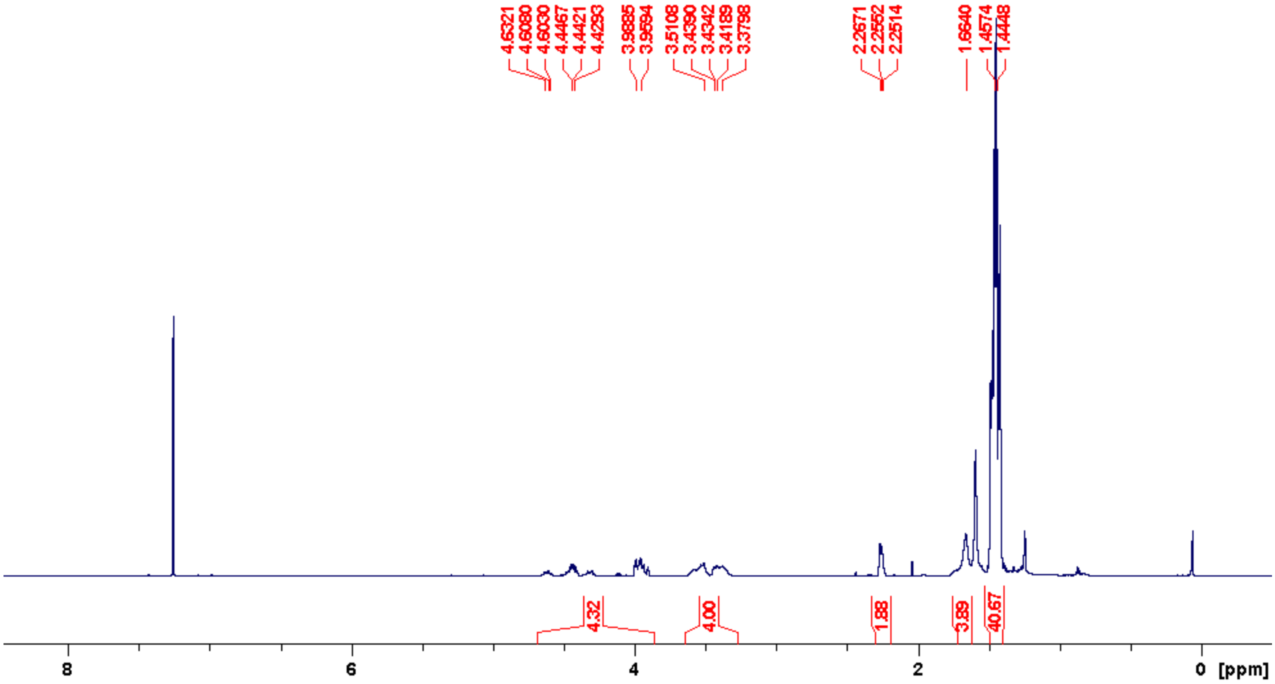

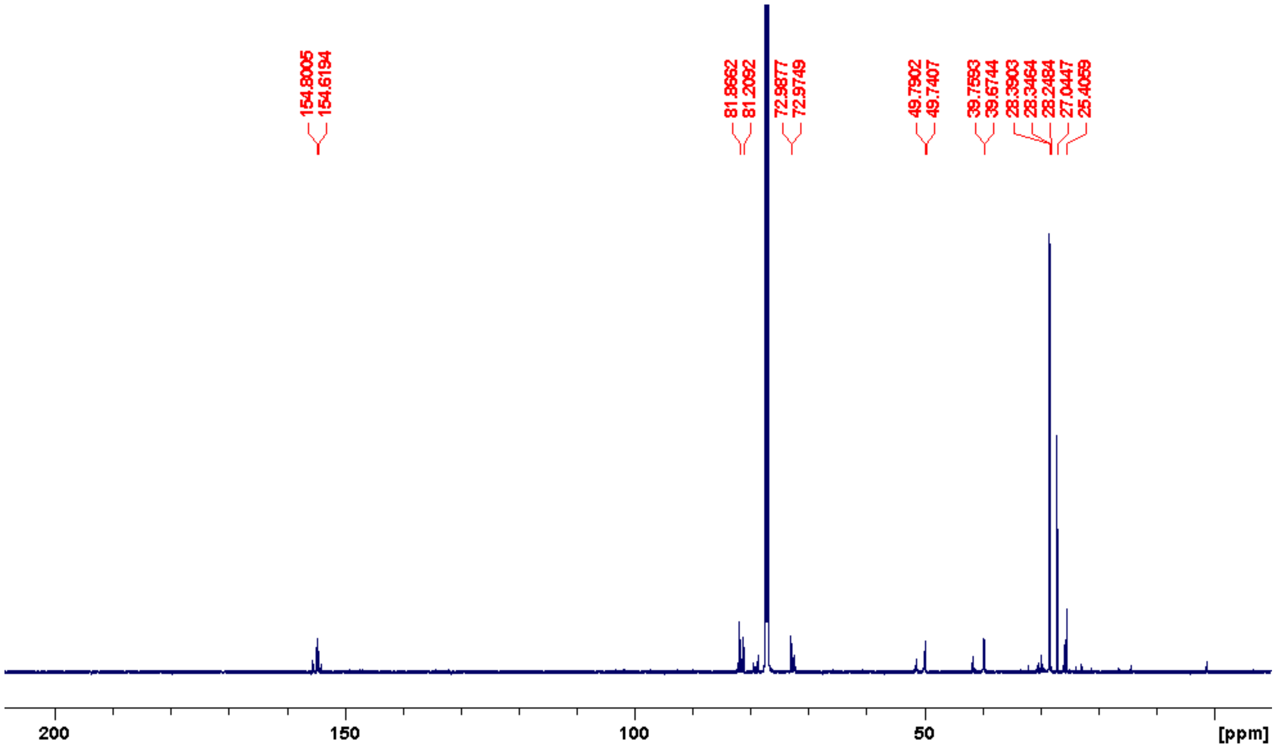

5.14 Compound **S12**, 2,2'-(butane-1,4-diyl)bis(4,5-dibromo-1-(prop-2-yn-1-yl)-1,2-dihydropyridazine-3,6-dione)

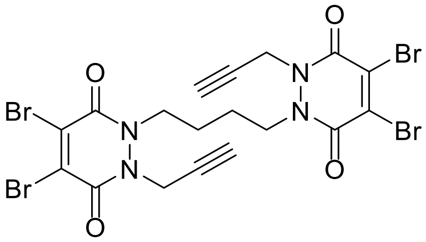

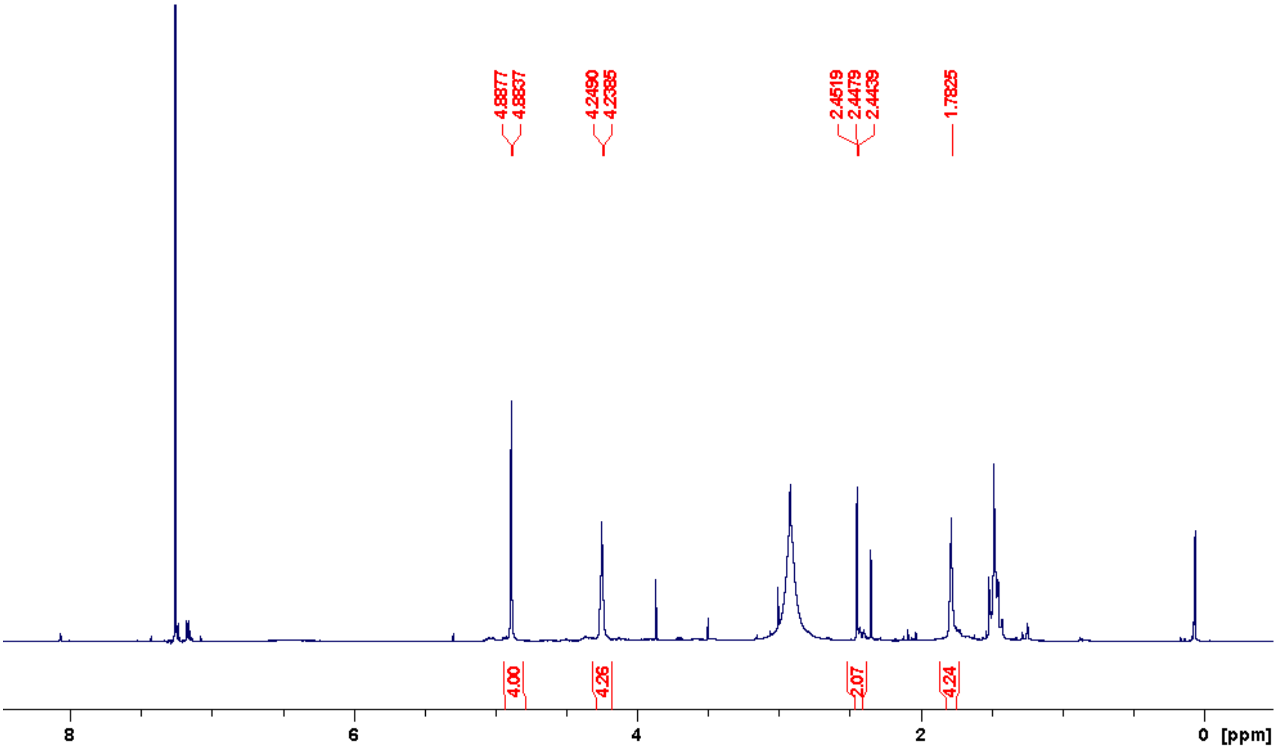

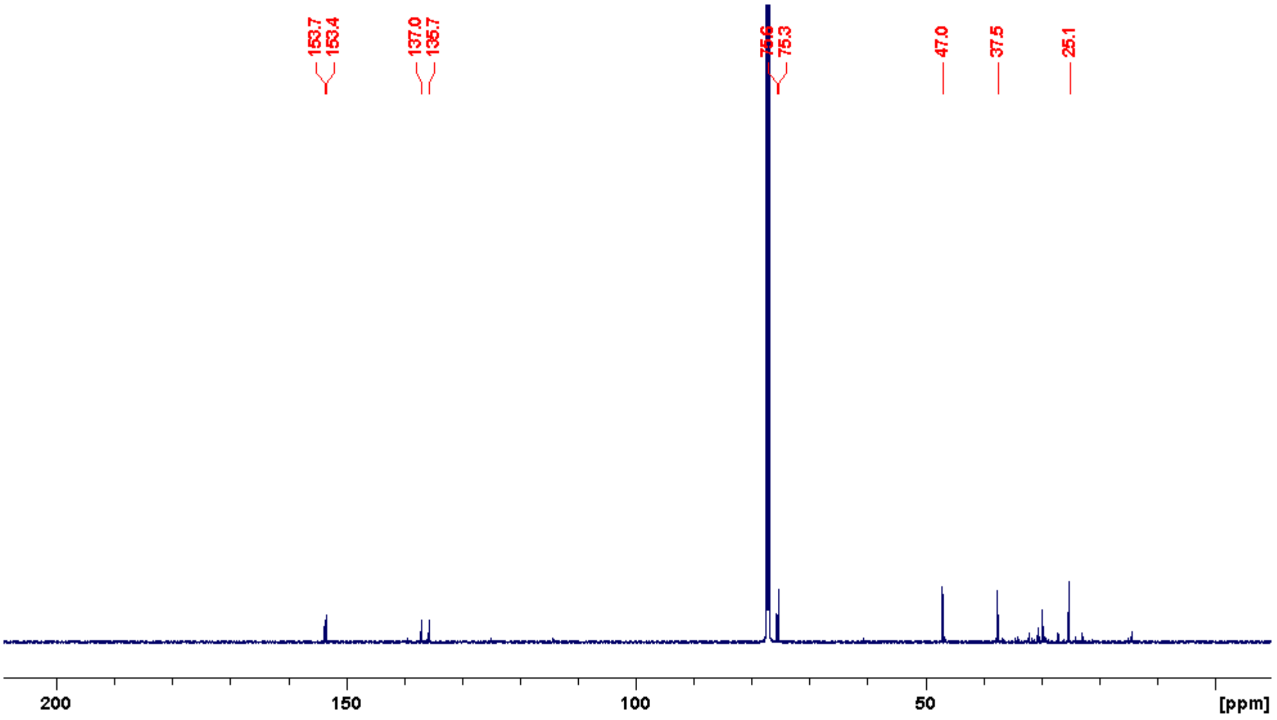

5.15 Compound **9**, N^1,$N^{1'}$-(((((((((butane-1,4-diylbis(4,5-dibromo-3,6-dioxo-3,6-dihydropyridazine-2,1(2*H*)-diyl))bis(methylene))bis(1*H*-1,2,3-triazole-4,1-diyl))bis(ethane-2,1-diyl))bis(oxy))bis(ethane-2,1-diyl))bis(oxy))bis(ethane-2,1-diyl))bis(oxy))bis(ethane-2,1-diyl))bis(N^5-(4-(6-methyl-1,2,4,5-tetrazin-3-yl)benzyl)glutaramide)

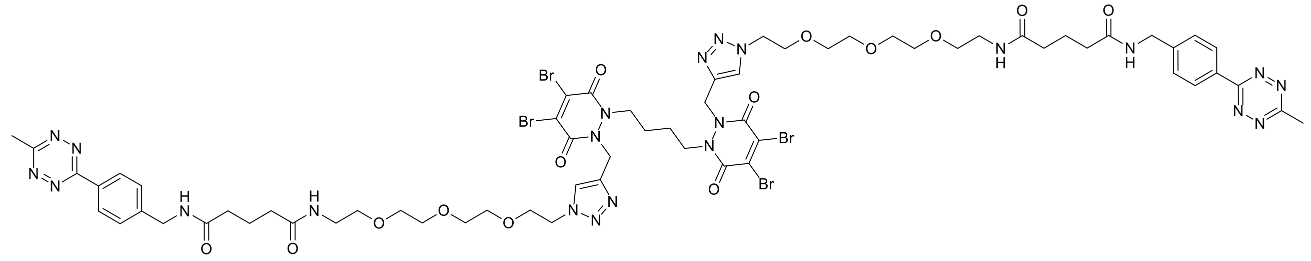

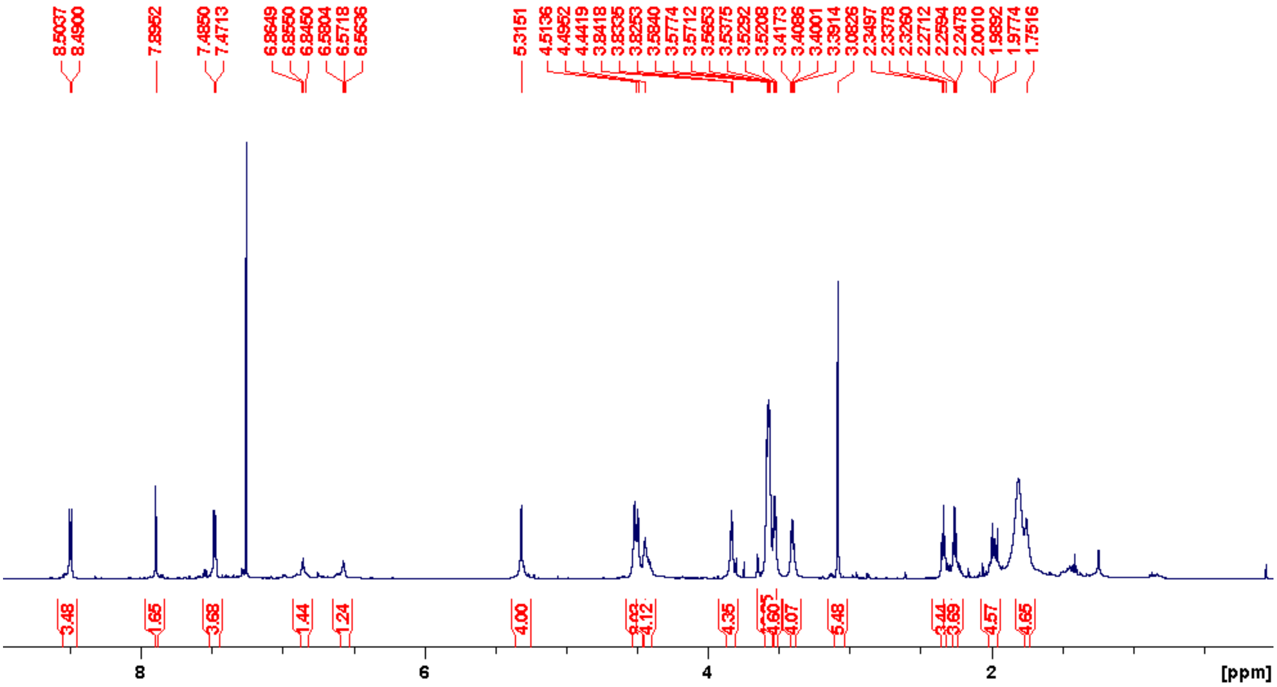

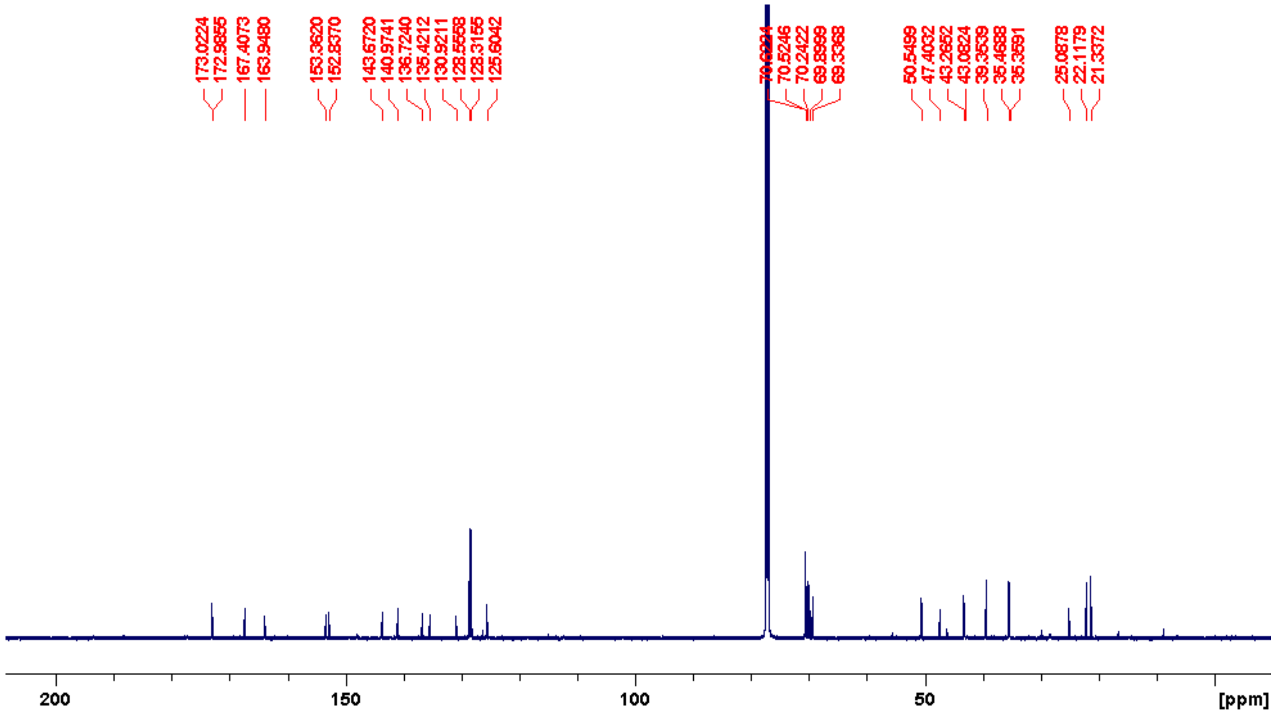

6. LC-MS spectra

6.1 Fc_{CD20}-$(Fab_{HER2})_2$ **1**

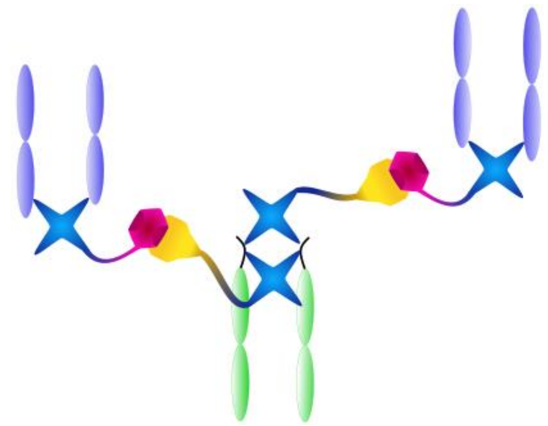

Expected: 147486.0 Da.

Observed: 73744.9 Da (HC_{CD20}-Fab_{HER2}, half-antibody), 147490.9 Da (Fc_{CD20}-$(Fab_{HER2})_2$), 148246.3 Da (Fc_{CD20}-$(Fab_{HER2})_2$ + 753 Da)

34776.5 Da -> PNGase F

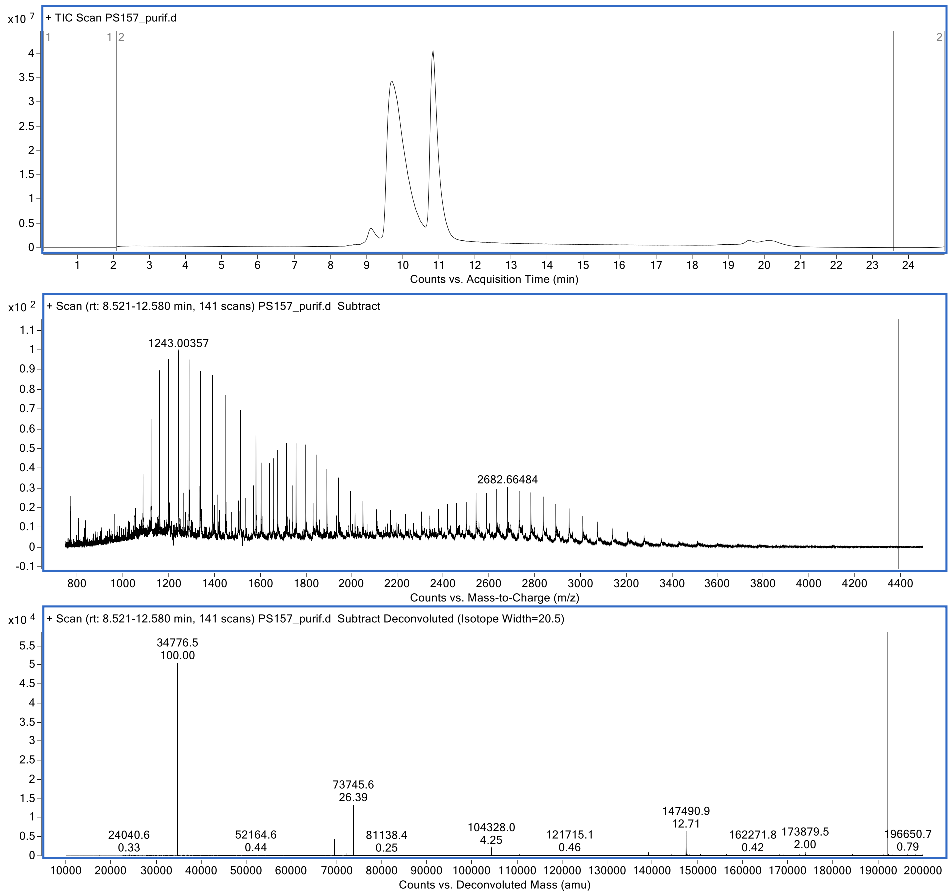

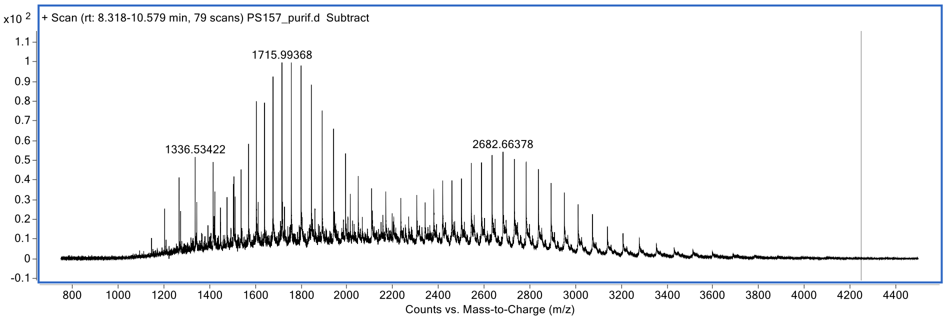
x10 2
+ Scan (rt: 8.318-10.579 min, 79 scans) PS157_purif.d Subtract
1715.99368
1336.53422
2682.66378
Counts vs. Mass-to-Charge (m/z)

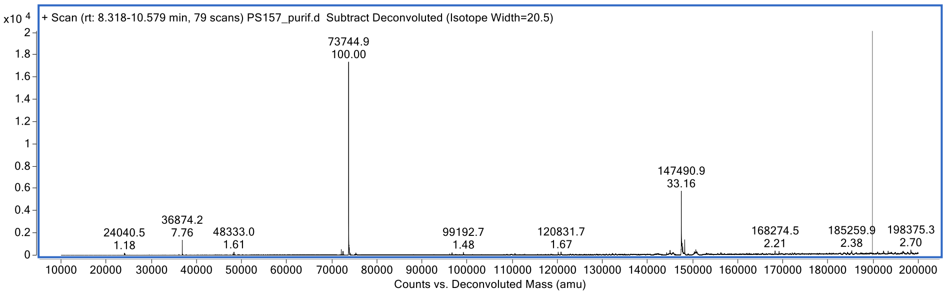
x10 4
+ Scan (rt: 8.318-10.579 min, 79 scans) PS157_purif.d Subtract Deconvoluted (Isotope Width=20.5)
73744.9
100.00
147490.9
33.16
24040.5
1.18
36874.2
7.76
48333.0
1.61
99192.7
1.48
120831.7
1.67
168274.5
2.21
185259.9
2.38
198375.3
2.70
Counts vs. Deconvoluted Mass (amu)

6.2 Fc_{CD20}-$(Fab_{HER2})_2$ **2**

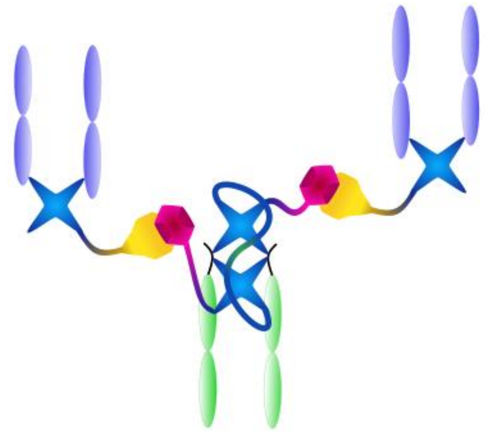

Expected: 147476.0 Da.

Observed: 147487.8 Da, 918.9 Da (A small molecule impurity. Doesn't correspond to either PD).

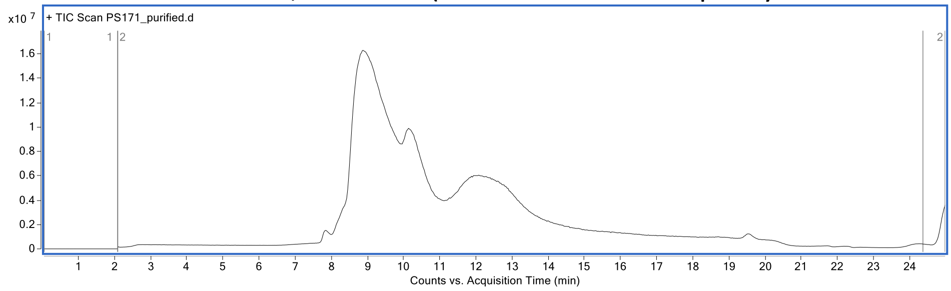

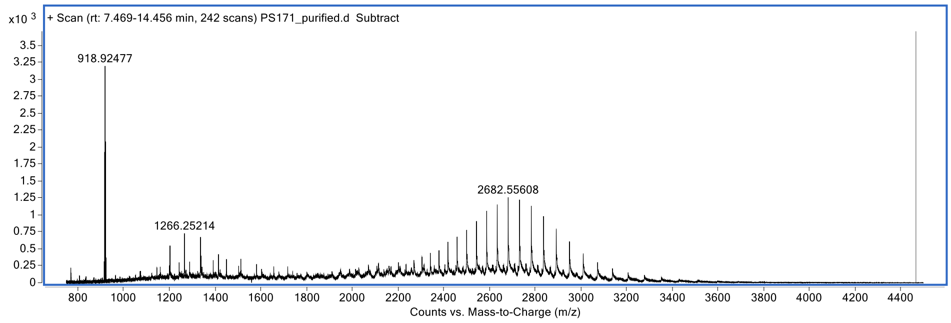

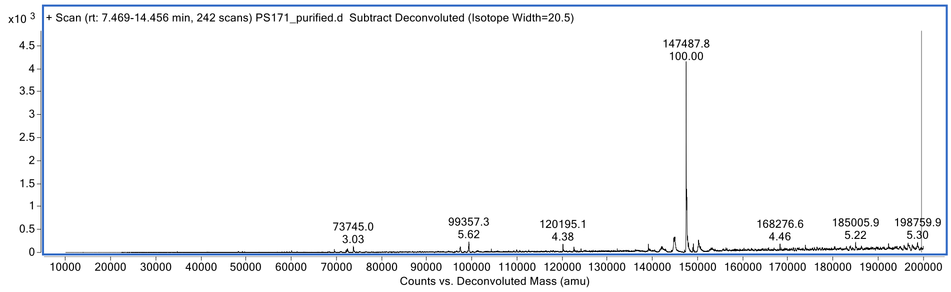

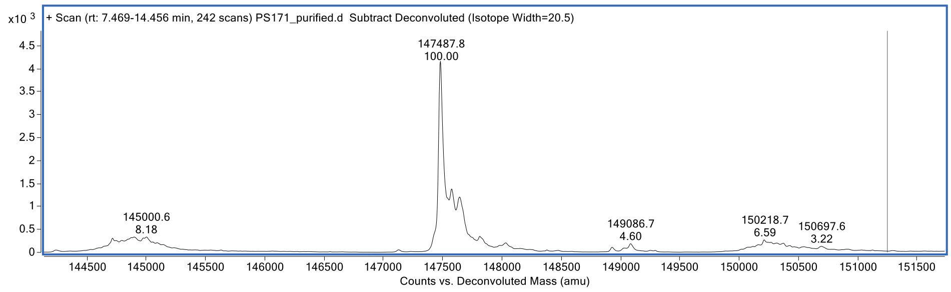

6.3 Fc_{CD20} **5**

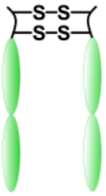

Observed mass: 49868.2 Da.
34776.3 Da -> PNGase F

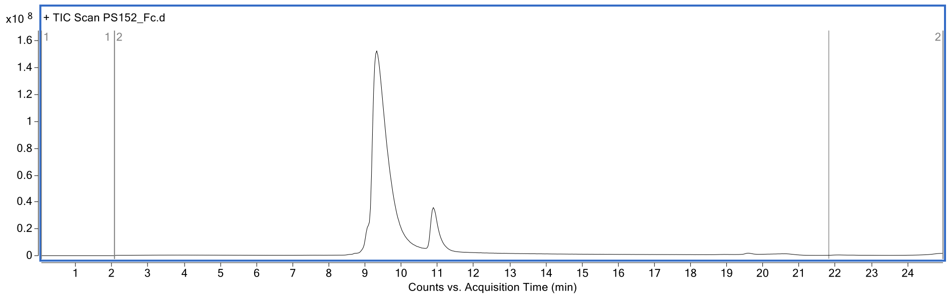

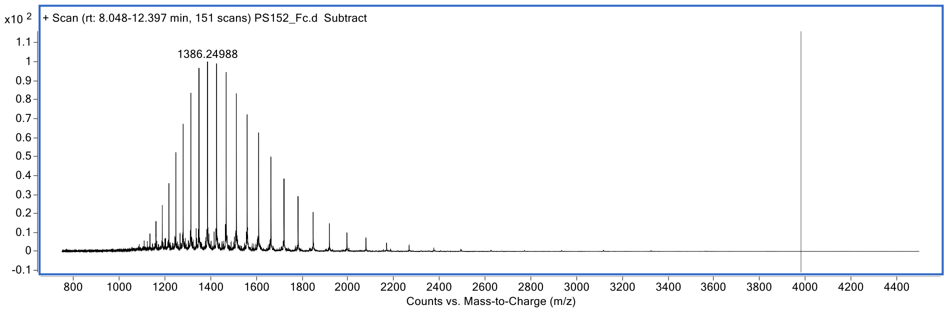

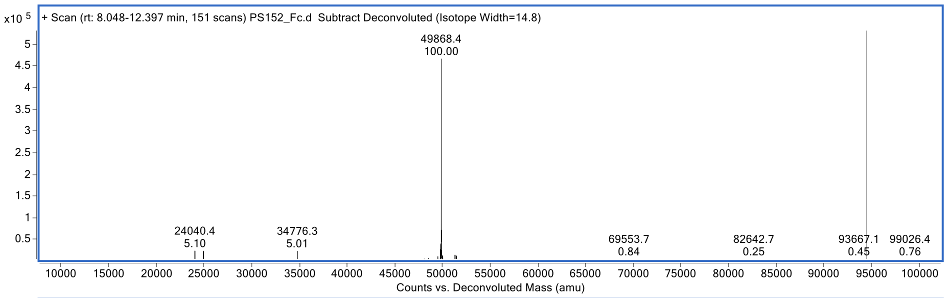

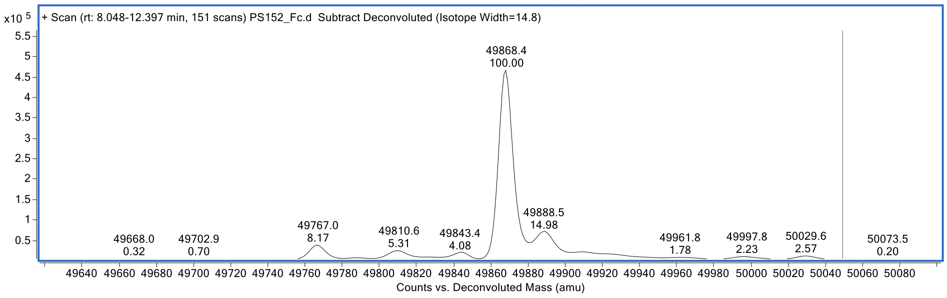

6.4 Fc_{CD20}-BCN_2 **6**

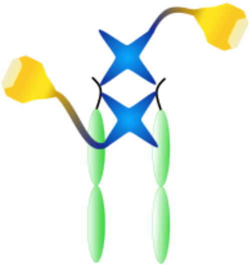

Expected: 50873.3 Da (PDAR 2).

Observed: 24039.7 Da (often present in LC-MS spectra of Fc_{CD20} or mAb_{CD20}. Doesn't correspond to any expected mass of heavy or light chain species, 25436.9 Da (HC + PD), 50873.7 Da (PDAR 2).

34776.6 Da -> PNGase F

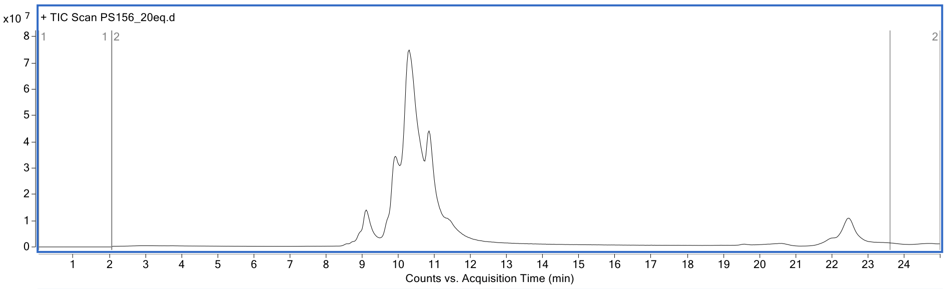

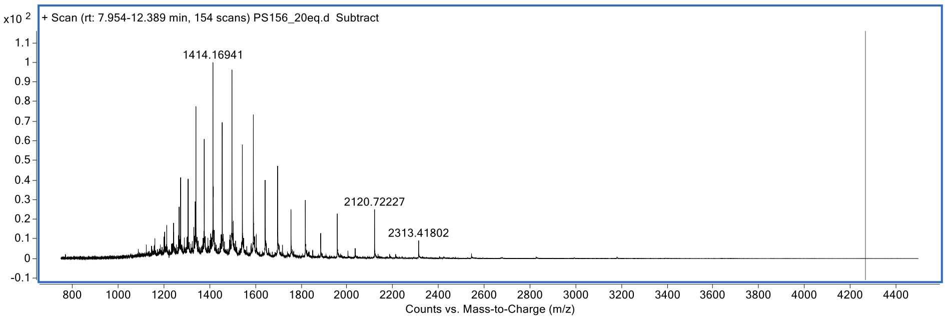

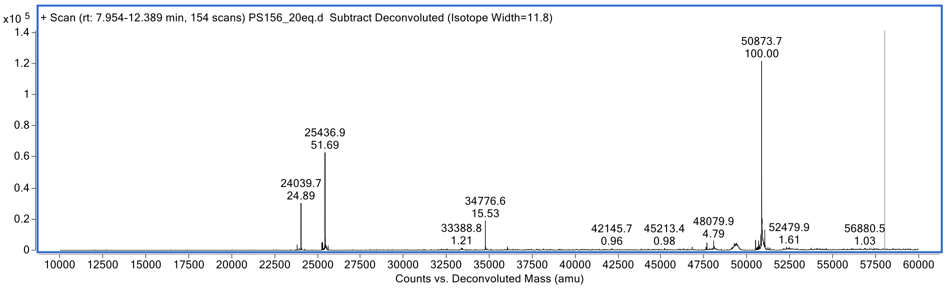

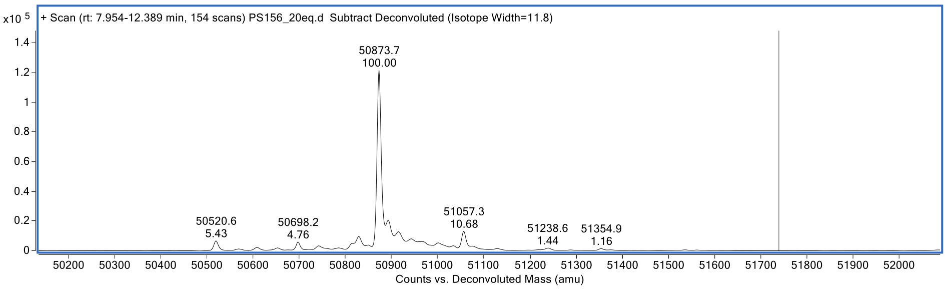

6.5 Fab_{HER2}-Tz **7**

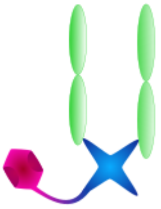

Expected mass: 48334 Da.

Observed mass: 48321 Da (as expected as MS was mis-calibrated by 14 Da, showing native Fab_{HER2} at 47625 Da).

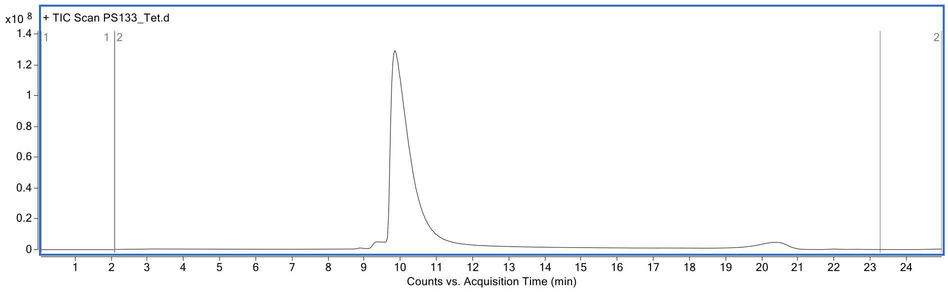

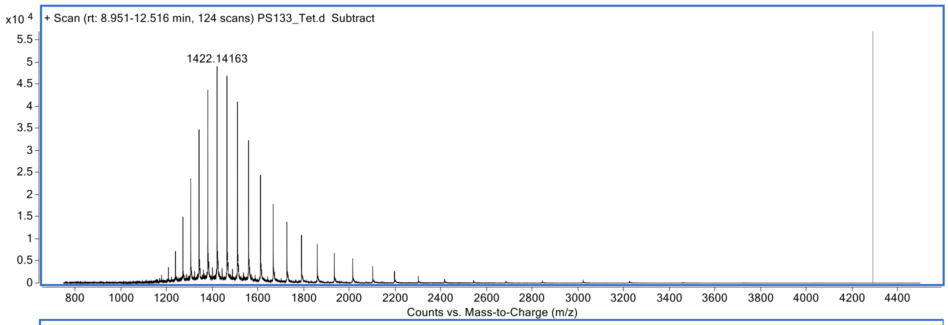

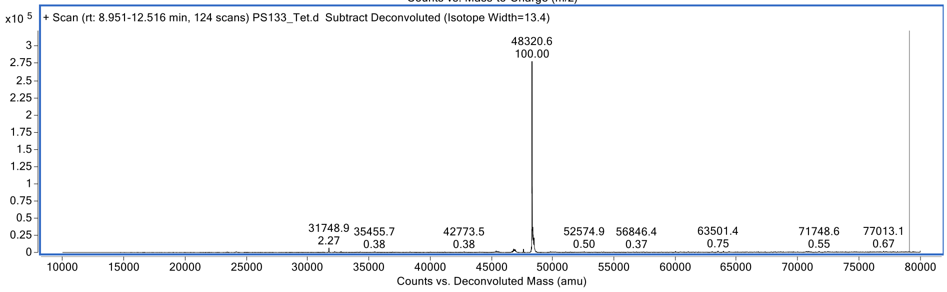

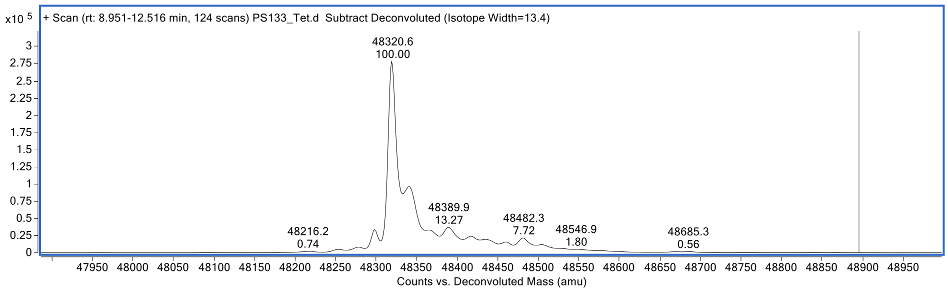

6.6 Fab_{HER2} **8**

Expected mass: 47638 Da. Observed mass: 47639 Da.

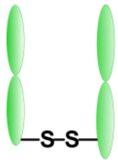

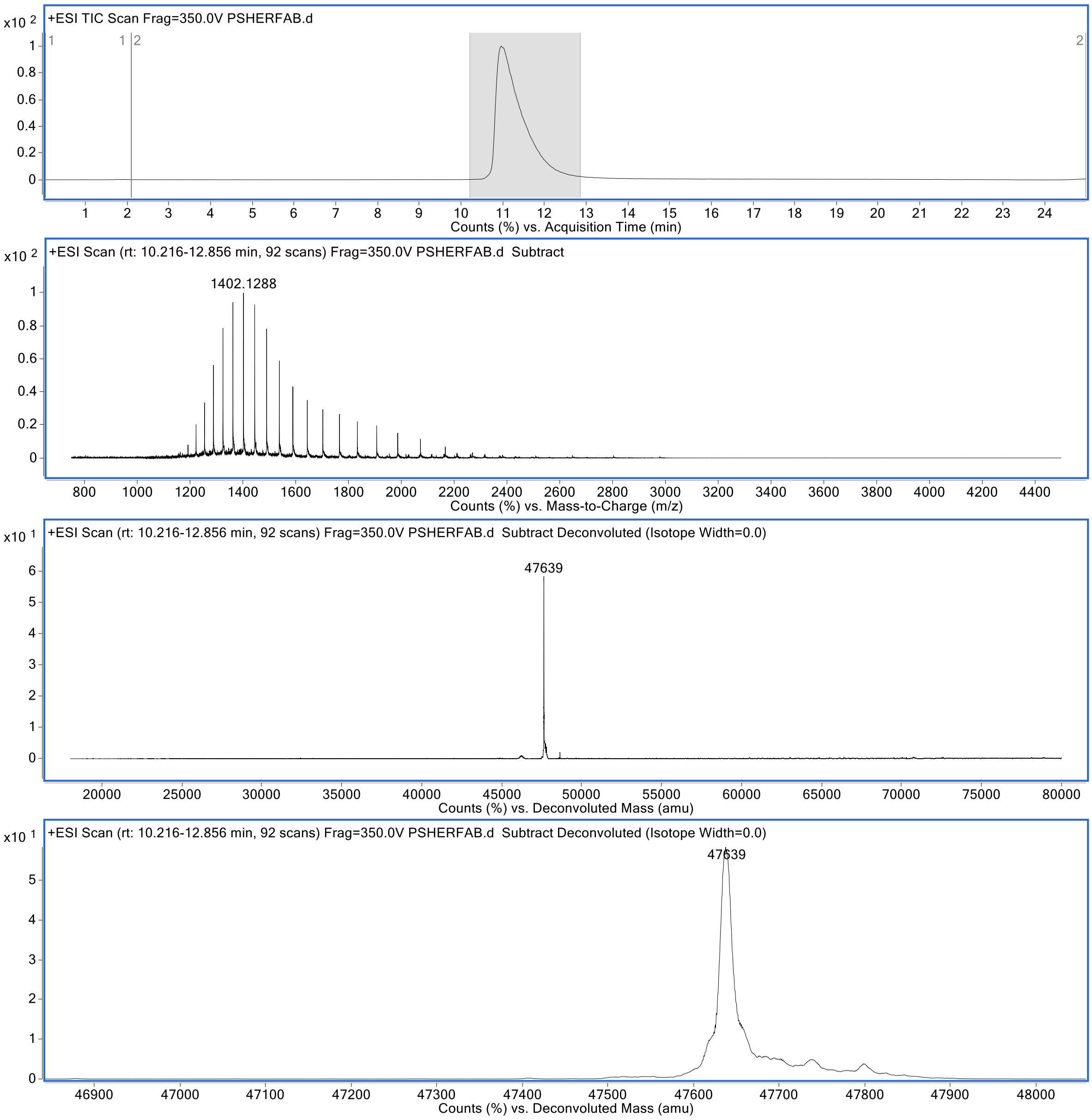

6.7 Fc_{CD20}-Tz_2 **10**

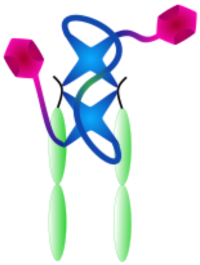

Expected: 51249.7 Da.

Observed: 51249.6 Da, 24039.9 Da, 48079.0 Da (24039.9 × 2).

34776.1 Da -> PNGAse F

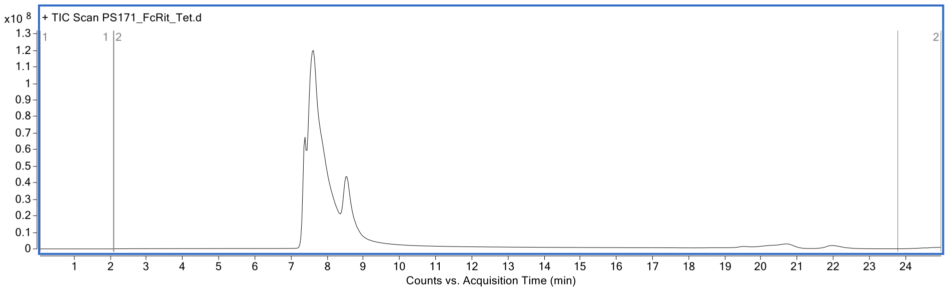

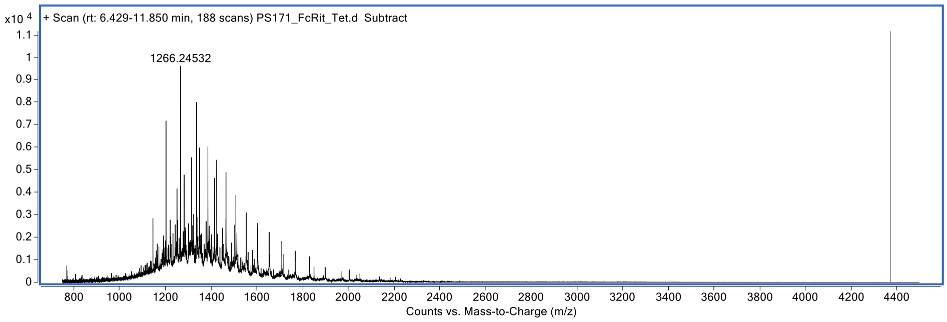

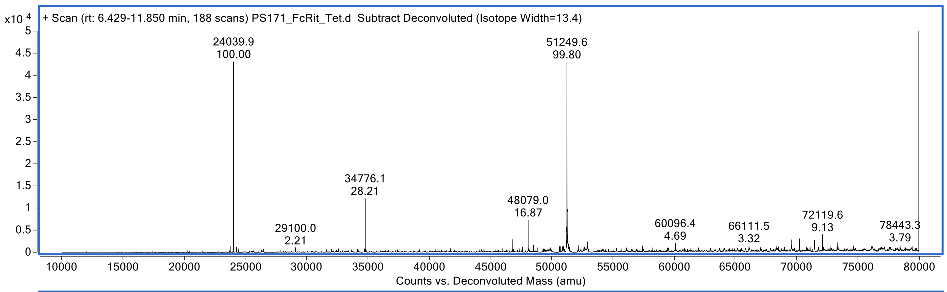

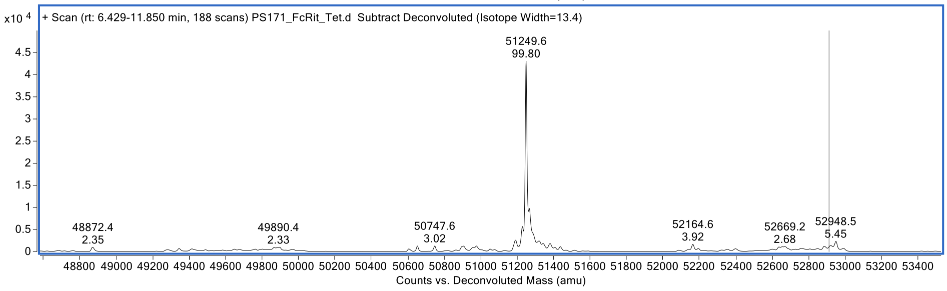

6.8 Fab$_{HER2}$-BCN **11**

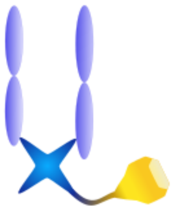

Expected mass: 48141.2 Da. Observed mass: 48140.8 Da.

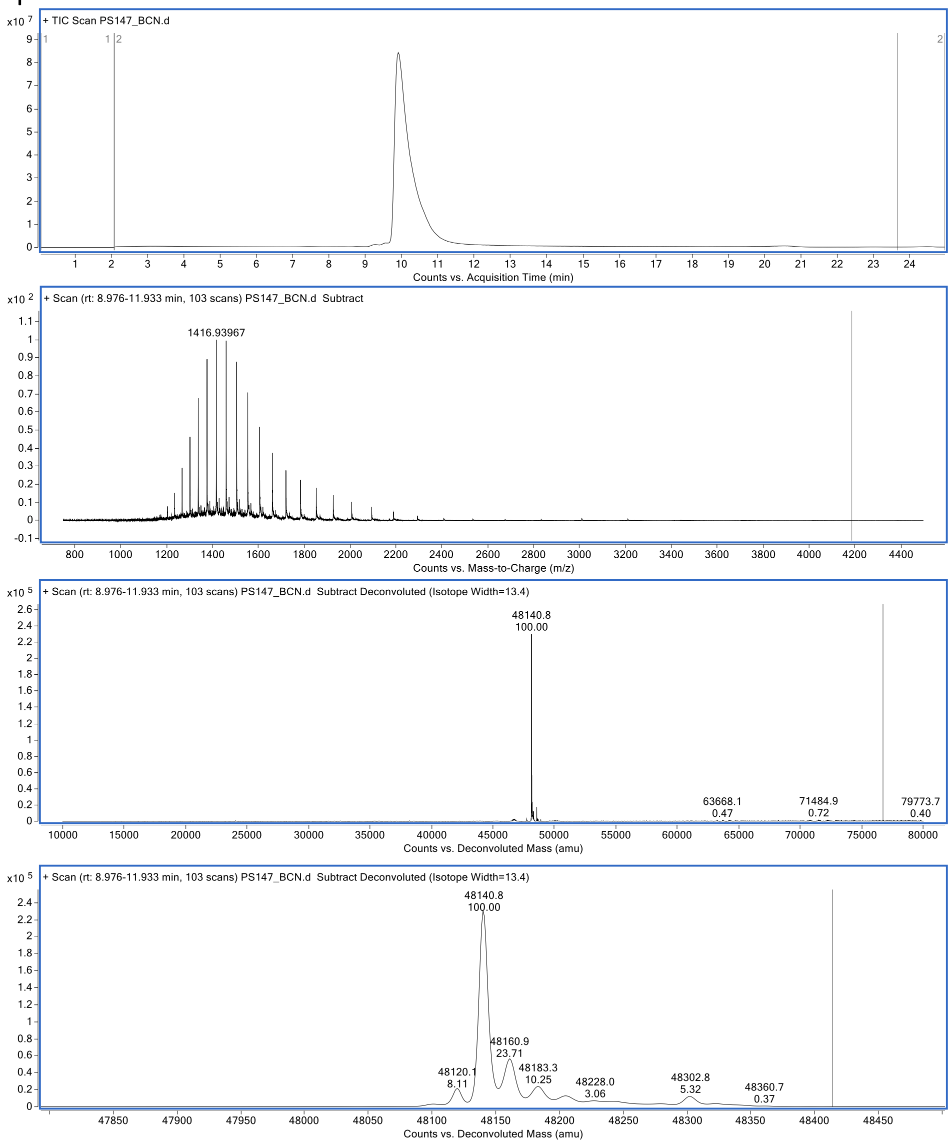

6.9 Fc$_{CD20}$-(Fab$_{HER2}$)-Tz **12**

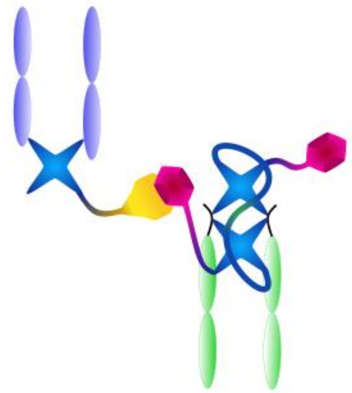

Expected: 99362.8 Da.

Observed: 99367.2 Da.

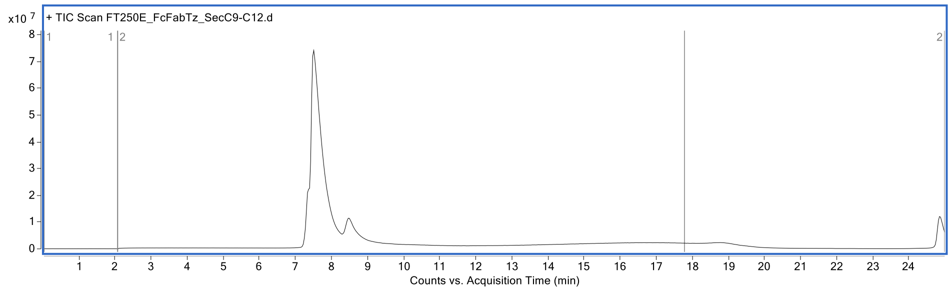

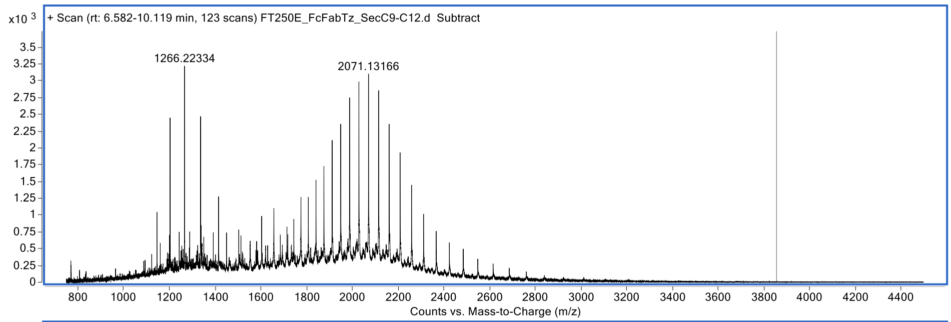

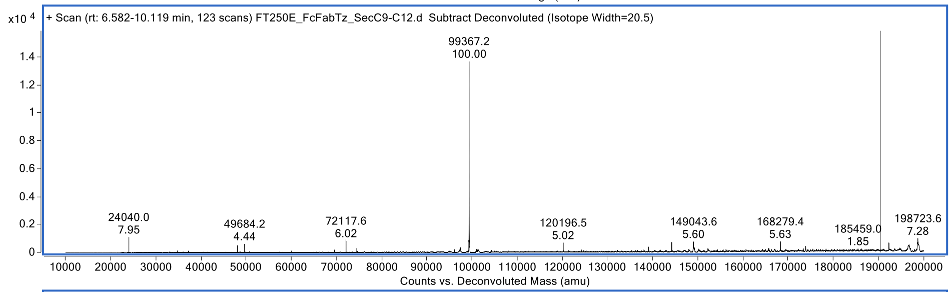

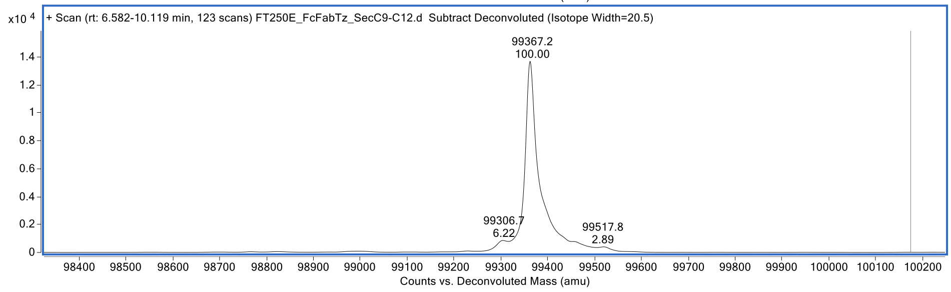

6.10 Fc$_{CD20}$-(Fab$_{HER2}$)-Fab$_{CD3}$ **13**

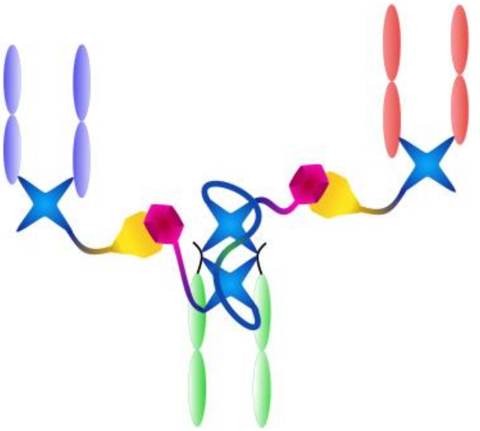

Expected: 147284.4 Da and 147398.4 Da (2nd peak derived from Fab$_{CD3}$ **14** containing two peaks).
Observed: 147291.9 Da, 147396.0 Da, 24040.4 Da (always present for Fc$_{RIT}$), 48079.2 Da (24040.4 × 2).
34776.0 Da (PNGAse F), 69552.4 Da (PNGAse F × 2), 104327.5 Da (PNGAse F × 3), 139103.8 Da (PNGAse F × 4), 173881.3 Da (PNGAse F × 5).

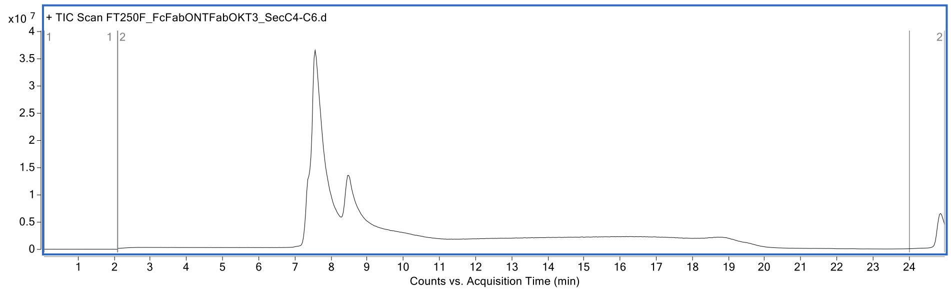

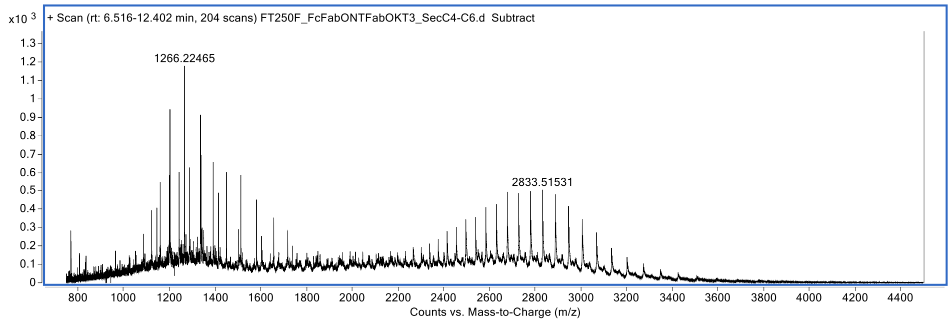

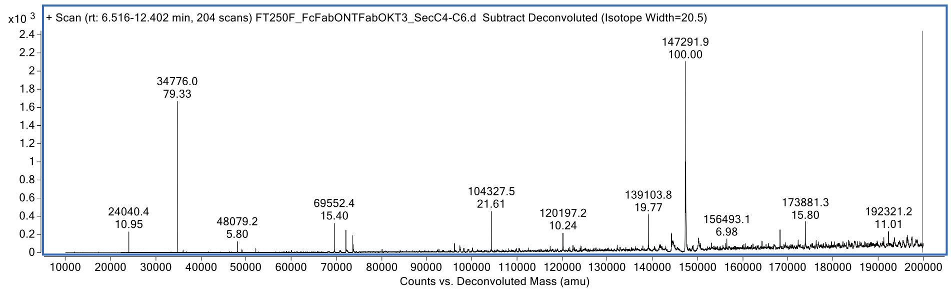

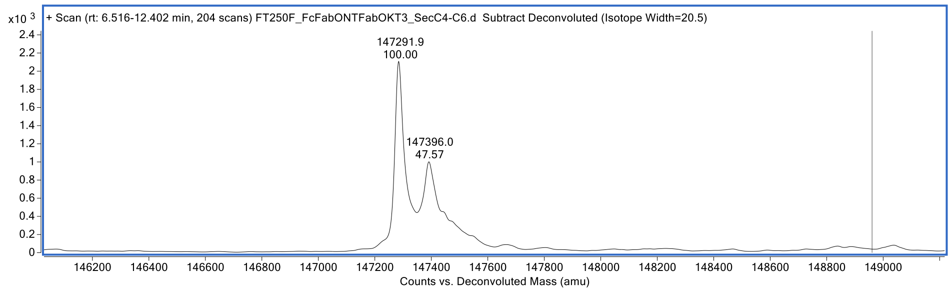

6.11 Fab$_{CD3}$ **14**

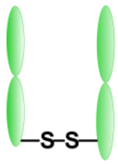

Observed mass: 47447 Da and 47560 Da.

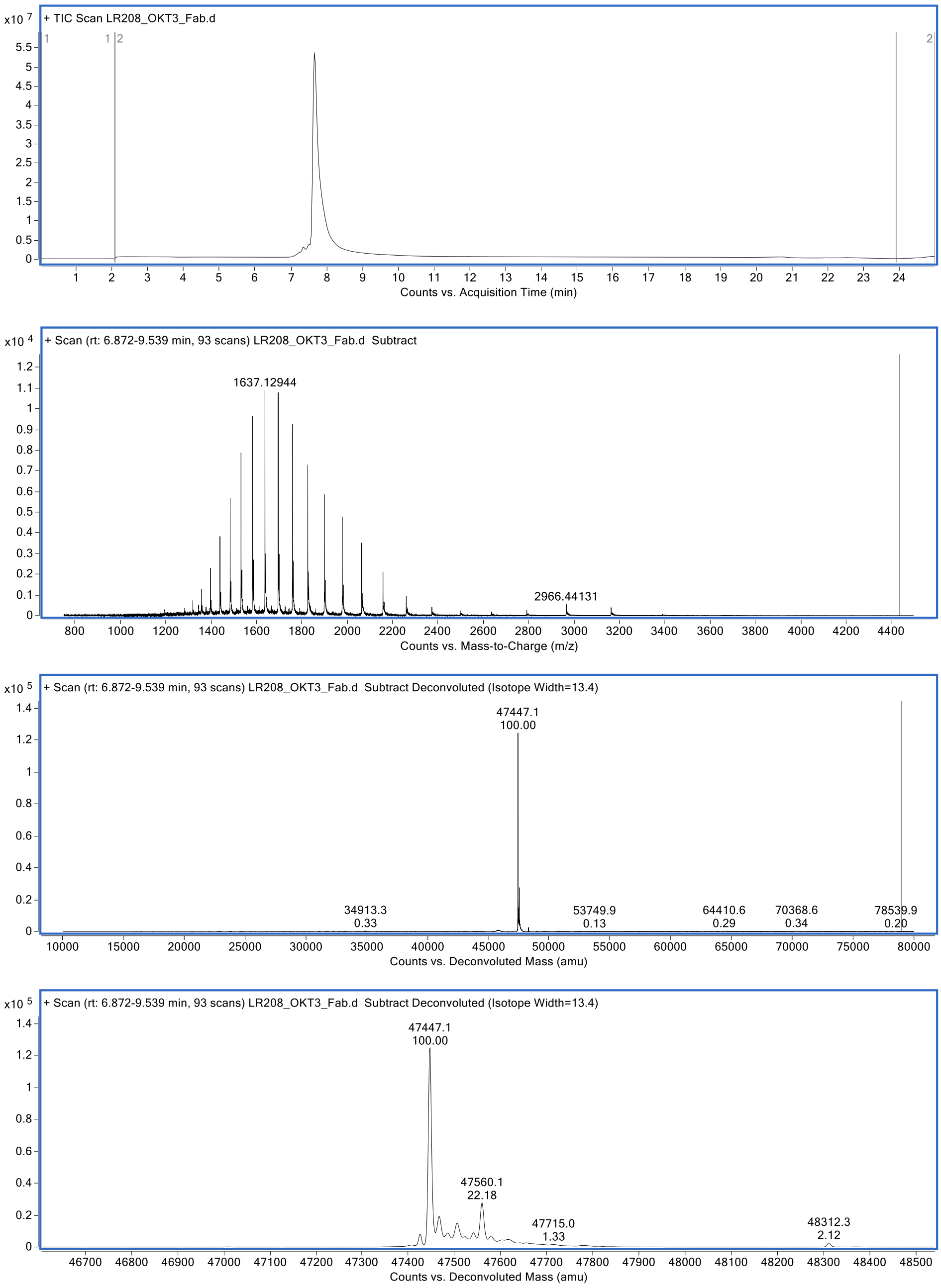

6.12 Fab$_{CD3}$-BCN **15**

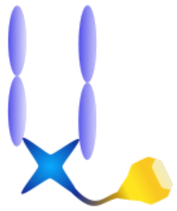

Expected mass: 47948 Da. Observed: 47950 Da.

Peak at 48062 Da of Δ = +112 Da could correspond to Fab$_{CD3}$-BCN with an additional leucine or isoleucine residue (mass: 113 Da).

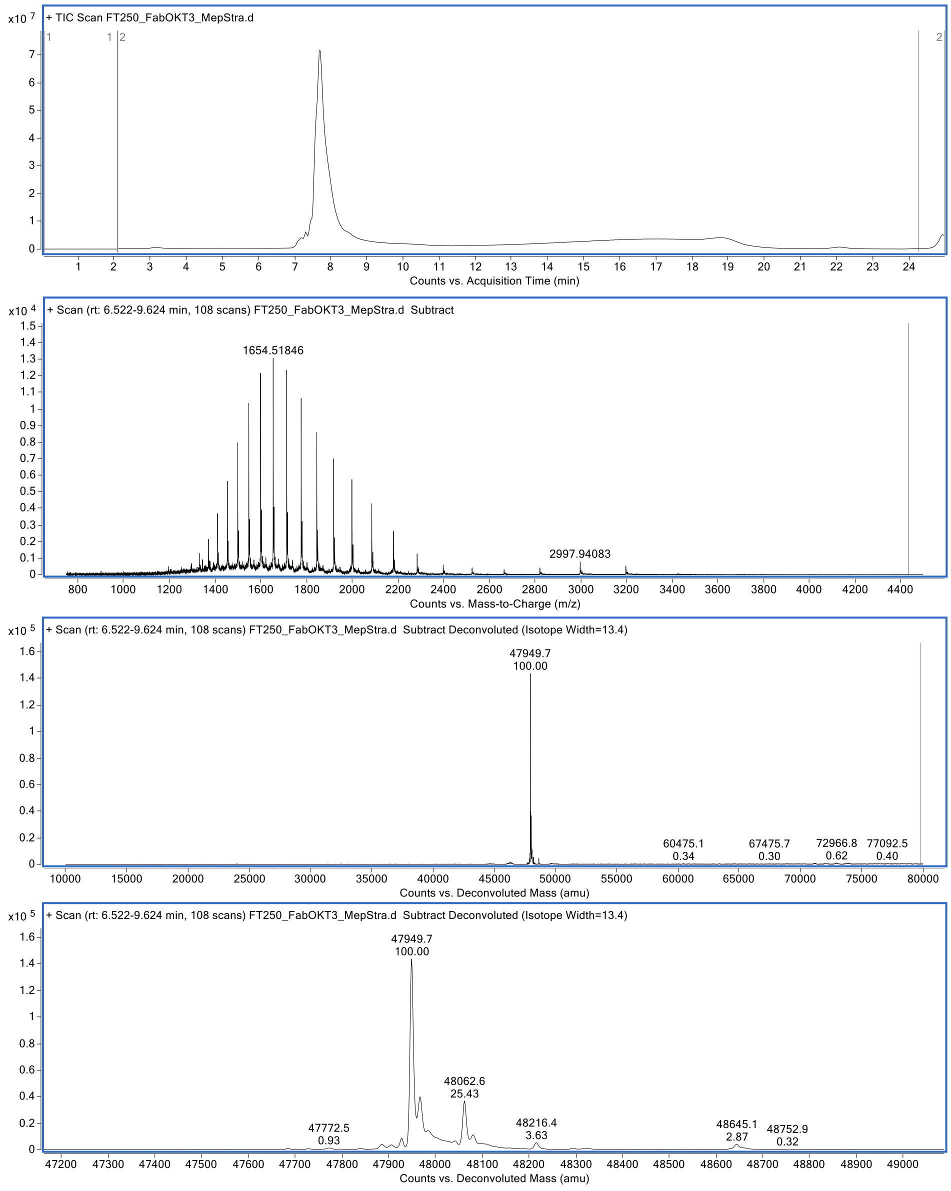

6.13 Fab_{CD20} **S13**

Expected mass: 47181 Da. Observed mass: 47180 Da.

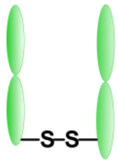

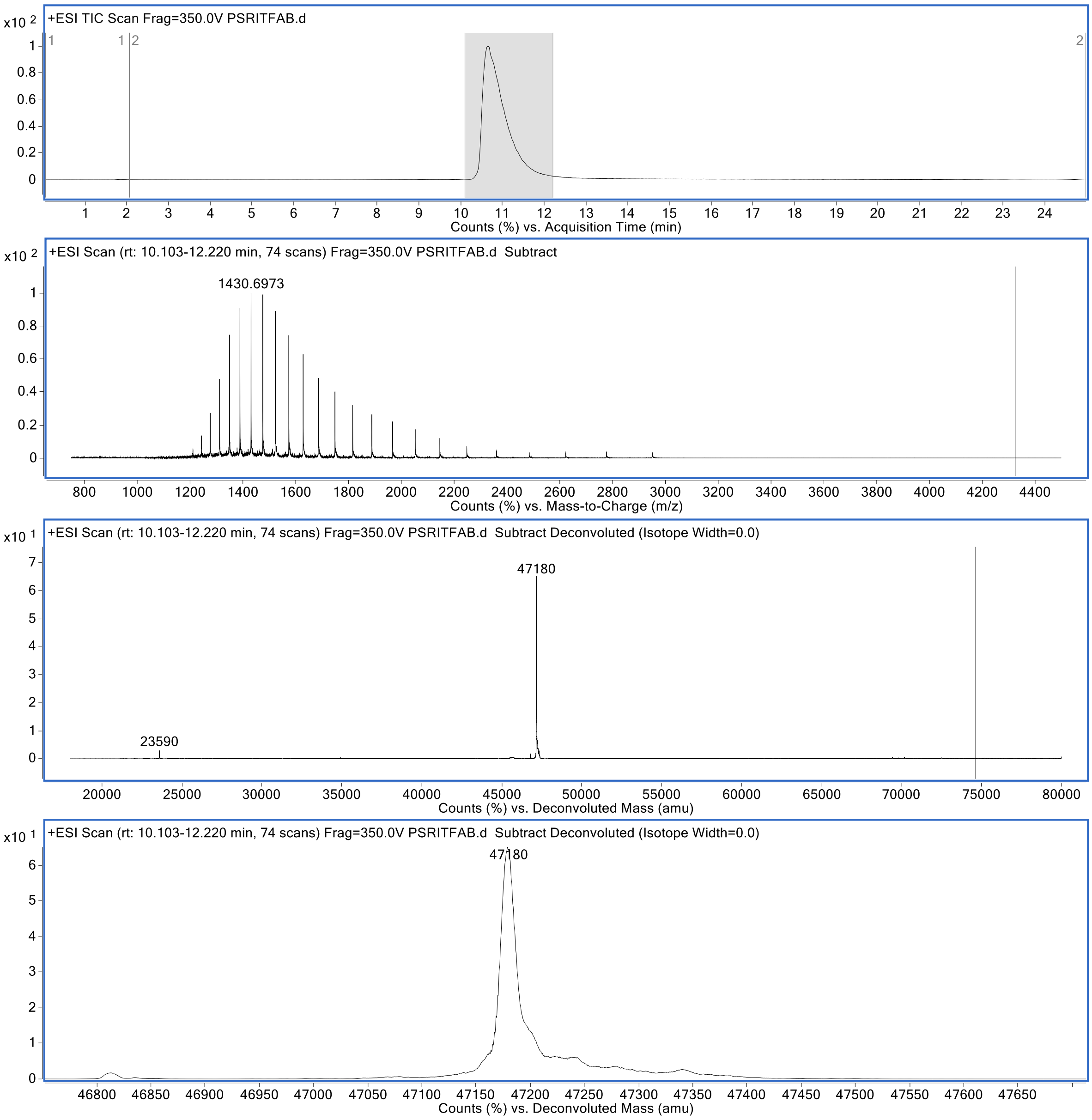

6.14 Fc_{CD20}-(Fab_{HER2})-BCN **S14.**

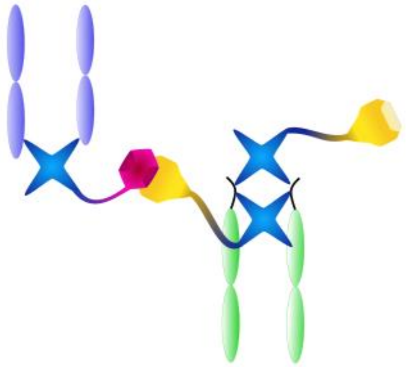

Expected: 99179.7 Da.
Observed: 24040.2 Da, 73744.9 Da (HC_{CD20}-Fab_{HER2}, half-antibody), 99184.5 Da (Fc_{CD20}-Fab_{HER2}), 147489.0 Da (Fc_{CD20}-(Fab_{HER2})$_2$).
34776.5 Da -> PNGase F

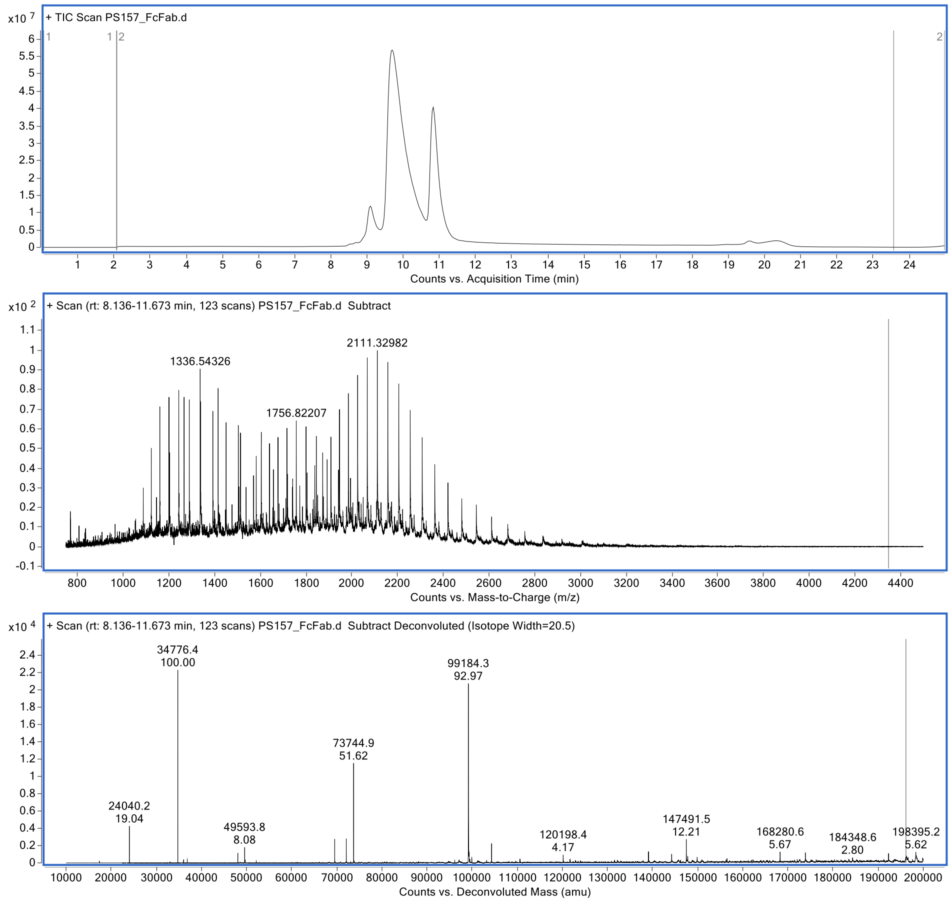

6.15 Fc_{CD20} + Br_2PD-Tz, 40 eq., 2 h

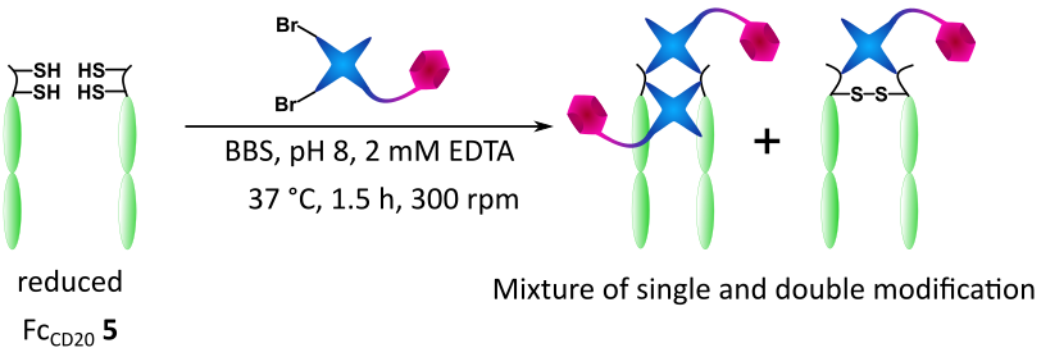

Expected: 50562.0 Da (PDAR 1), 51255.8 Da (PDAR 2).

Observed: 24040.0 Da (always seen in Fc_{RIT} LC-MS), 25629.9 Da (HC + PD), 50564.0 Da (PDAR 1), 51259.8 Da (PDAR 2).

34776.6 Da -> PNGase F

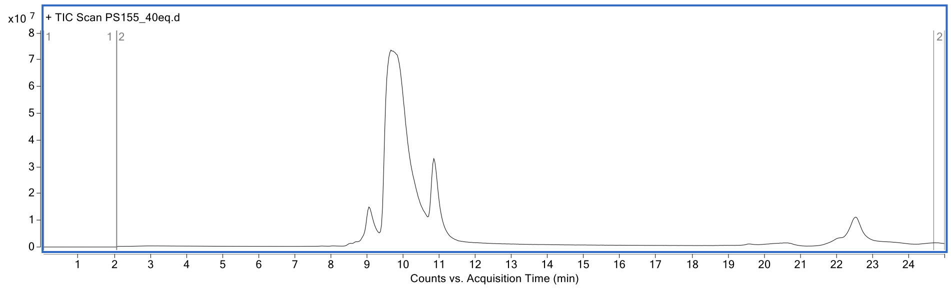

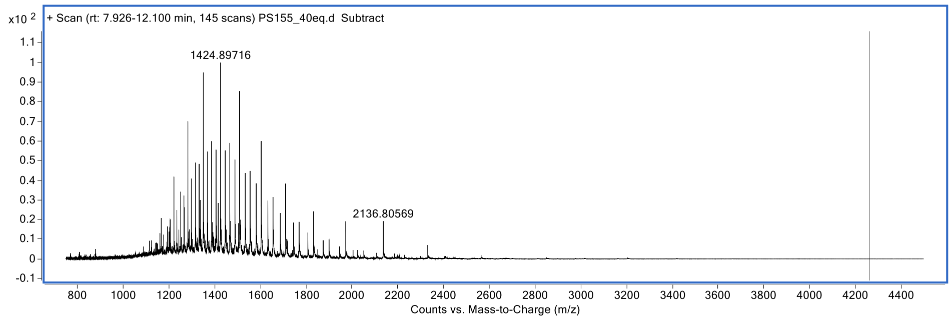

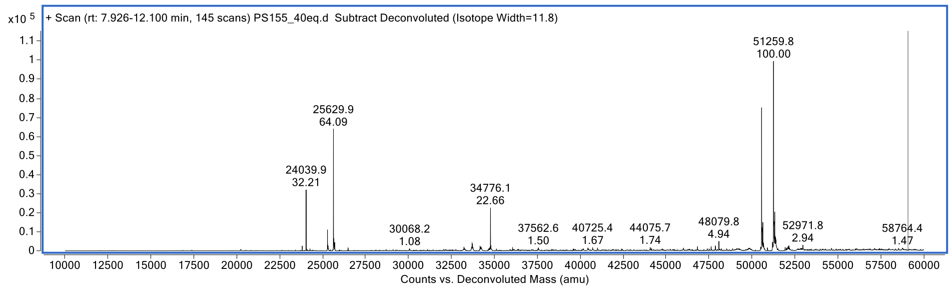

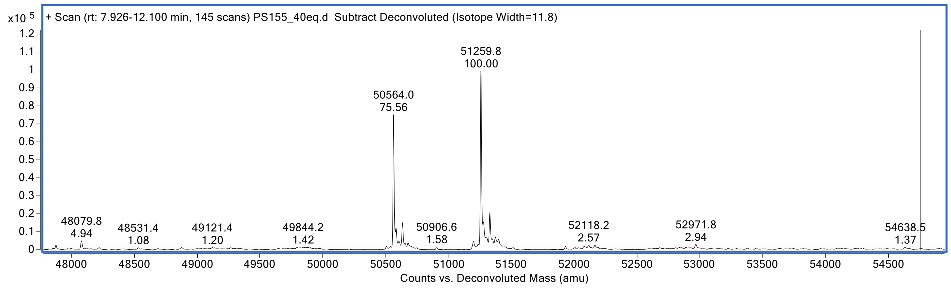

Supplement: Supplementary file 1 — oc2c01437_si_001.pdf [file oc2c01437_si_001.pdf]
